# Evolutionary conserved networks of human height identify multiple Mendelian causes of short stature

**DOI:** 10.1038/s41431-019-0362-0

**Published:** 2019-02-26

**Authors:** Nadine N. Hauer, Bernt Popp, Leila Taher, Carina Vogl, Perundurai S. Dhandapany, Christian Büttner, Steffen Uebe, Heinrich Sticht, Fulvia Ferrazzi, Arif B. Ekici, Alessandro De Luca, Patrizia Klinger, Cornelia Kraus, Christiane Zweier, Antje Wiesener, Rami Abou Jamra, Erdmute Kunstmann, Anita Rauch, Dagmar Wieczorek, Anna-Marie Jung, Tilman R. Rohrer, Martin Zenker, Helmuth-Guenther Doerr, André Reis, Christian T. Thiel

**Affiliations:** 1 Institute of Human Genetics, Friedrich-Alexander-Universität Erlangen-Nürnberg FAU, Erlangen, Germany; 2 Bioinformatics, Department of Biology, Friedrich-Alexander-Universität Erlangen-Nürnberg, Erlangen, Germany; 3 Centre for Cardiovascular Biology and Disease, Institute for Stem Cell Biology and Regenerative Medicine (inStem), Bangalore, India; 4 The Knight Cardiovascular Institute, Departments of Medicine, Molecular and Medical Genetics, Oregon Health and Science University, Portland, OR USA; 5 Institute of Biochemistry, FAU Erlangen-Nürnberg, Erlangen, Germany; 6 Molecular Genetics Unit, Casa Sollievo della Sofferenza Hospital, IRCCS, San Giovanni Rotondo, Italy; 7 Department of Orthopedic Rheumatology, Friedrich-Alexander-Universität Erlangen-Nürnberg (FAU), Erlangen, Germany; 8 Institute of Human Genetics, University of Leipzig, Leipzig, Germany; 9 Institute of Human Genetics, University of Würzburg, Würzburg, Germany; 10 Institute of Medical Genetics, University of Zurich, Zurich, Switzerland; 11 Institute of Human Genetics, University of Duisburg-Essen, Essen, Germany; 12 Institute of Human-Genetics, Medical Faculty of University Düsseldorf, Düsseldorf, Germany; 13 Division of Pediatric Endocrinology, Department of General Pediatrics and Neonatology, Saarland University Medical Center, Homburg/Saar, Germany; 14 Institute of Human Genetics, Otto‐von‐Guericke University Magdeburg, Magdeburg, Germany; 15 Department of Pediatrics and Adolescent Medicine, Friedrich-Alexander-Universität Erlangen-Nürnberg FAU, Erlangen, Germany

**Keywords:** DNA sequencing, Disease genetics, Genetic counselling

## Abstract

Height is a heritable and highly heterogeneous trait. Short stature affects 3% of the population and in most cases is genetic in origin. After excluding known causes, 67% of affected individuals remain without diagnosis. To identify novel candidate genes for short stature, we performed exome sequencing in 254 unrelated families with short stature of unknown cause and identified variants in 63 candidate genes in 92 (36%) independent families. Based on systematic characterization of variants and functional analysis including expression in chondrocytes, we classified 13 genes as strong candidates. Whereas variants in at least two families were detected for all 13 candidates, two genes had variants in 6 (*UBR4*) and 8 (*LAMA5*) families, respectively. To facilitate their characterization, we established a clustered network of 1025 known growth and short stature genes, which yielded 29 significantly enriched clusters, including skeletal system development, appendage development, metabolic processes, and ciliopathy. Eleven of the candidate genes mapped to 21 of these clusters, including *CPZ, EDEM3, FBRS, IFT81, KCND1, PLXNA3, RASA3, SLC7A8, UBR4, USP45*, and *ZFHX3*. Fifty additional growth-related candidates we identified await confirmation in other affected families. Our study identifies Mendelian forms of growth retardation as an important component of idiopathic short stature.

## Introduction

Human height is a heritable and highly heterogeneous trait [1]. Efforts to understand the genetic basis of growth have employed genome-wide association studies (GWAS) to systematically assess the effect on human height variation of common variants with a minor allele frequency > 5% [2]. 697 variants, mainly located in 423 noncoding loci, have been implicated in height variance in the population [2, 3]. Subsequent studies on rare variants, both at the nucleotide and genomic levels, further expanded the number of associated loci [3, 4]. So far, rare and common height-associated variants together explain about 27.4% of height heritability [3]. In addition, it is known from Mendelian forms of growth retardation that rare, large effect-size variants can have extremely large effects on growth development [3].

Short stature, defined auxologically as a height two standard deviations below the mean height in the population, affects about 3% of individuals and is a common medical concern. In a recent study combining systematic phenotyping and exome-based sequencing, we were able to identify a genetic cause in up to 33% of individuals with idiopathic short stature (ISS) [5]. Consequently, 67% of the affected individuals remained undiagnosed. Most forms of short stature have been attributed to Mendelian causes, highlighting defects in a diverse range of functional pathways [6, 7]. The most common monogenic causes include defects of the *SHOX* gene (2.4%) [8], heterozygous variants in *ACAN* (1.4%) [9] and many genes for rare syndromic forms as well as skeletal dysplasias [8, 10–12]. At least 477 genes have been found to affect human growth [13], but as yet there are no reliable estimates of the number of growth-associated genes. For most of these genes, though, no association with short stature has been found in humans. Affected individuals and their families would thus benefit from the identification of further genes associated with growth retardation. In this study in 254 unrelated individuals with ISS and their families, we used exome sequencing to identify and characterize novel candidate genes based on evolutionarily conserved networks.

## Materials and methods

### Individuals

The study was approved by the ethics committee of the Friedrich-Alexander-Universität Erlangen-Nürnberg (FAU). 565 individuals and their families were referred by medical specialists for evaluation of growth retardation after endocrine defects of the growth hormone pathway and other organic causes of growth retardation were excluded. After previous targeted testing including array analysis in some and exome sequencing failed to identify a known cause, we further assessed the exomes of 254 well-characterized families with at least 1 offspring whose growth standard deviation score (SDS) was ≥2 below the mean population height and/or the estimated family (est. height in Table 1) for potential candidate genes (Table 1 and Supplementary Table 2). Participants’ mean age (±standard deviation) was 9.2 ± 0.43 years, and 155/254 (61%) participants were female. Most index individuals (53%) presented with a height between 3 and 2 SDS below the mean, and 32% were born small for gestational age (Supplementary Figure 1). In 68/254 (27%) participants, additional features such as microcephaly, syndactyly, nail dysplasia or any nonspecific facial gestalt resulted in a diagnosis of syndromic short stature.

**Table 1:** Clinical characteristics of 254 individuals with idiopathic short stature after exclusion of known causes

Characteristic	No. (%)
Age group	
<4 yrs	43 (17)
>4 yrs	211 (83)
Small for gestational age	81 (32)
Short stature [SDS]^a^	
−2 to −3	135 (53)
−3 to −4	55 (22)
−4 to −5	12 (5)
<−5	4 (2)
Below est. height^b^	48 (19)
Short stature type	
Isolated	186 (73)
Syndromic	68 (27)
Head circumference [SDS]	
>−2	153 (60)
−2 to −3	38 (15)
−3 to −5	32 (13)
<−5	3 (1)
Not available	28 (11)
IQ	
Normal	203 (80)
70–85	51 (20)
Sex	
Female	155 (61)
Male	99 (39)
Bone age	
Accelerated	11 (4)
Normal	21 (8)
Delayed	66 (26)
Not available	156 (61)

^a^All 254 affected individuals presented with a height below the est. final adult height (est. height)

^b^Affected individuals with a height above −2 SDS, but below the est. height

### Exome sequencing and variant assessment

We performed whole-exome sequencing in 185 affected individuals and both of their respective parents (trio analysis) and in 69 affected individuals (affected-only analysis) after enrichment by SureSelect targeted capturing on HiSeq 2500 (94.3%) or SOLiD 5500xl (5.7%). Exomes were analyzed by semiautomatic selection and data quality inspection of variants, followed by the interpretation in relation to the reported phenotypic spectrum (Supplementary Figures 2 and 3). Variants and familial segregation were confirmed by Sanger sequencing.

**Fig. 1:**
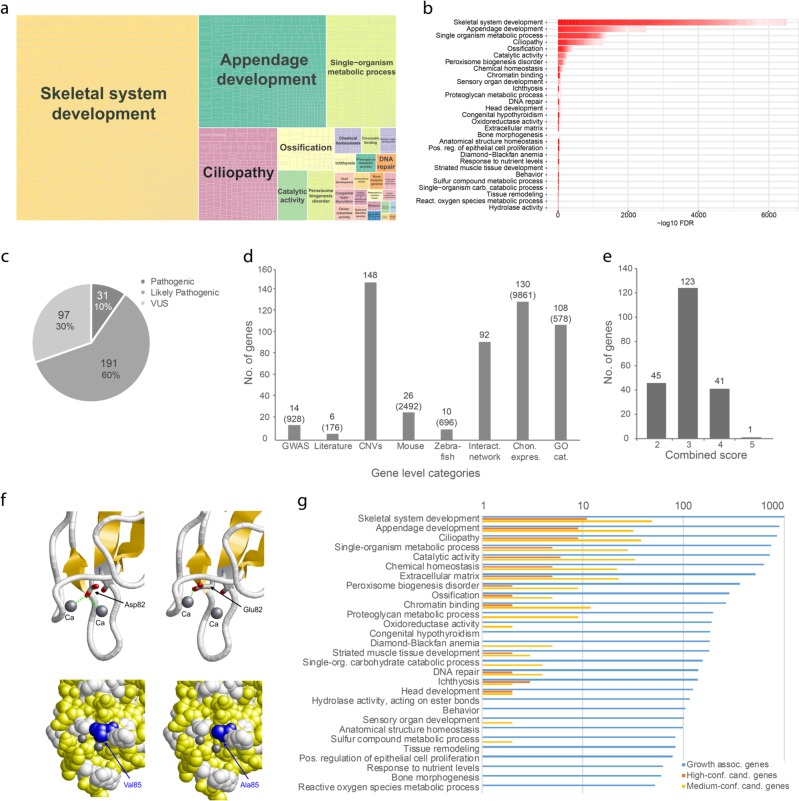
Enrichment analysis and candidate gene characterization. **a** Functional clustering of 1,025 known growth and short stature genes (**b**) into 29 biological clusters for growth. **c** Variant level assessment. Number of variants affecting function or likely affecting function and variants of unknown significance identified (Supplementary Tables 1, 2). **d** Categories of gene level assessment. Numbers represent the genes to which each category applies (see Supplementary Table 4). Numbers in brackets represent the genes among all selected known growth and short-stature genes. **e** The results of the variant and gene level evaluation were merged to a combined score (Shown is the highest score for each gene, Supplementary Table 5). **f** Based on structure analysis of variants of unknown significance (VUS), 5 variants in 4 of the high-confidence candidate genes were reclassified to likely pathogenic. Model of the RASA3 C2-domain showing the site of the Asp82Glu and Val85Ala variants. Both residues are located in a pocket of the C2 domains that contains two Ca^2+^ ions (Ca). Asp82 forms interactions with both Ca^2+^ ions (green dotted lines), whereas the longer glutamate side chain of the Asp82Glu variant can only interact with one of the Ca^2+^ ions, probably leading to a loss of the second Ca^2+^ ion from the binding pocket. Val85 (blue) is located on the lateral wall of this pocket, and the shorter alanine side chain in the Val85Ala variant affects the width of the pocket. **g** Distribution of the 63 high- and medium-confidence candidate genes in the growth-associated clusters

All 185 trios were analyzed for variants with de novo, compound heterozygous, homozygous, and X-linked recessive inheritance. We classified variants according to their population frequency and potential effect on gene function (Supplementary methods, Fig. 1c and Supplementary Tables 1–3). Population data from gnomAD was considered as most likely appropriate controls. After excluding benign and likely benign variants, we evaluated affected genes for their relevance to growth phenotypes. For this gene-level assessment, we included information from association studies, copy number variants, gene ontology (GO) terms, protein–protein interaction data, suitable mouse and zebrafish models, and a previous exome study [14] (Fig. 1d and Supplementary Table 4). Additional data on expression in chondrocytes was obtained by RNA sequencing (RNASeq) of cartilage tissue for all genes studied (Fig. 1d, Supplementary Methods and Figure 4). The combined results of the variant-level and gene-level assessments were then used to identify genes, which were further investigated with respect to the observed mode of inheritance in the 69 affected-only exomes (Fig. 1e, Supplementary Methods, Supplementary Figure 2 and Supplementary Table 5). Genes were finally classified as high-confidence and medium-confidence candidates based on the number of affected individuals and the combined variant-level and gene-level scores (Supplementary Table 6 and Supplementary Methods). Variants were uploaded to ClinVar (https://www.ncbi.nlm.nih.gov/clinvar/, Submission ID SUB5032330).

### Functional clustering analysis

Functional enrichment analysis was performed using the Database for Annotation, Visualization, and Integrated Discovery, which comprises 1025 Genes using the keywords “growth delay” from the Human Phenotype Ontology (HPO) database and “short stature” from Online Mendelian Inheritance in Man (OMIM) and MedGen (Supplementary methods). Proteins were submitted to DAVID using human gene Ensembl identifiers. Significantly overrepresented annotation terms were retrieved with the options GOTERM_MF_ALL, GOTERM_CC_ALL, GOTERM_BP_ALL, KEGG_PATHWAY, UP_KEYWORDS, and OMIM_DISEASE. A false discovery rate (FDR) of 0.05 by the Benjamini and Hochberg approach was used to determine significant enrichment using all human gene Ensembl identifiers in the BioMart database as the background (Supplementary methods). Functional Annotation Clustering was performed using the DAVID functional annotation clustering tool with the following parameters: overlap = 5, initialSeed = 5, finalSeed = 5, linkage = 0.5, kappa = 20. This tool implements a fuzzy clustering algorithm to cluster functional annotation terms based on the degree of the overlap between associated genes. Raw *p*-values were used to compute the initial clusters. Annotation terms with an FDR > 0.05 were subsequently pruned. Given a list of candidate genes and an annotation term, we calculated (i) the odds of a human gene (Ensembl gene identifier) in the list being associated with the annotation term; and (ii) the odds of a human gene (Ensembl gene identifier) not in the list being associated with the term. The odds ratio was calculated by dividing the odds from (i) by the odds from (ii) using the fisher.test () function implemented in the R environment for statistical computing. Results were presented as a treemap based on the functional annotation clustering using R’s treemap package.

### Protein structure analysis

For all variants of unknown significance in high-confidence candidate genes and for missense variants in RASA3, we performed in silico structural analyses and reclassified variants based on predicted functional consequences (Fig. 1f and Supplementary Figure 8). Models for all wild-type protein domains were either obtained from Modbase [15] or modelled using HHpred [16] and Modeller [17]. The variant amino acid was exchanged using Swiss-Model [18] and visualized with RasMol [19]. Detailed information on individual modelling is provided in the Methods section in the supplementary information.

## Results

### Identification of novel candidate genes for ISS

Characterization of involved variants and genes and consideration of independent affected individuals carrying these variants revealed 63 candidate genes in 92 (36%) of the 254 affected individuals included in the analysis. We classified 13 genes as high-confidence genes and the remaining 50 genes as medium-confidence candidates (Tables 2 and 4, Supplementary Tables 7 and 11, and Supplementary Figures 6–18). The mode of inheritance was mainly autosomal dominant (71%), followed by X-linked recessive (17%), and autosomal recessive (11%). The most abundant variants were missense variants (78%), followed by nonsense (15%), splice site (6%), and non-frameshift insertion / deletion variants (1%). Nonsense variants were identified in the high-confidence candidate genes *RASA3* and *USP45*. Based on in silico structural analysis, we reclassified 5 variants in 4 of the high-confidence candidate genes from “variants of unknown significance” (VUS) to “likely pathogenic” (Fig. 1f and Supplementary Table 7).

**Table 2:** Categories, function and phenotype overview of identified high-confidence candidate genes

Gene	No. pats.	Variant level classification^e^			Gene level classification [No. of cat.]^c^	Combined score [1–4]	Main pathway^d^	Phenotype			
		V	IV	III				Heigth [SDS ± s]	OFC [SDS ± s]	Syndromic	Common features (no. of individuals)
CPZ	3^a^		3		1	3		−3.6 ± 1.4	−2.3 ± 1	0/3	Short neck (2/3), hypertelorism (2/3), low set ears (3/3), wide intermammillary distance (2/3)
EDEM3	2		2		3	3	Protein processing	−2 ± 0.1	−1.7 ± 0.7	0/3	High arched eye brows (2/2), thin upper lip (2/2)
FBRS	3^a^		2	1	3	3		−2.2 ± −0.2	−2 ± 0.4	0/3	Thin hair (2/3), prominent ears (2/3), thin lips (2/3), brachydactyly (2/3)
IFT81	2		2		2	3	Cilium Assembly	−2.9 ± 0	−3.1 ± 1	0/3	wide nasal bridge (2/2), high arched eye brows (2/2)
KCND1	2		2		1	3	Cardiac conduction	−2.9 ± 0.4	−2.4 ± 1.1	0/2	Fair hair (2/2), sparse eye brows (2/2), thin lips (2/2), brachydactyly (2/2)
LAMA5	8^b^		4	4	4	3	Human papillomavirus infection	−2.9 ± 0.6	−1.4 ± 1.1	2/8	Thin lips (4/8), barrel chest (5/8), sandal gap (6/8)
MED24	2		1	1	5	4	Thyroid hormone signaling	−2.5 ± 0.4	−1.5 ± 0.3	0/2	Thin lips (2/2), brachydactyly (2/2)
PLXNA3	2			2	5	3	Axon guidance	−2 ± 0.3	−0.8 ± 1	0/2	Lateral hypoplasia of brows (2/2), broad thumbs (2/2)
RASA3	2	1	1		4	3–4	Ras signaling	−2.7 ± 0.5	−2 ± 0	0/2	Broad nasal tip (2/2), thin lips (2/2), barrel chest (2/2)
SLC7A8	2		2		3	3	Protein degradation	−2.9 ± 0.7	−2 ± 0.5	0/2	Brachydactyly (2/2)
UBR4	6^b^		5	1	4	2–3	Human papillomavirus infection	−2.9 ± 0.5	−1 ± 1.2	3/6	Lateral sparse brows (4/6), barrel chest (4/6), brachydactyly (5/6)
USP45	4	1	2	1	2	2–4	DNA Repair	−2.9 ± 0.7	−1.6 ± 1.1	0/4	Brachydactyly (4/4)
ZFHX3	2		1	1	5	3–4	Regulat.pluripotency of stem cells	−2.5 ± 0.7	−2 ± 0	0/2	Brachydactyly (2/2), thin lips (2/2), barrel chest (2/2)

^a^Segregation not available in 1 individual

^b^Segregation not available in 4 individuals

^c^maximum of 8 gene level categories

^d^Pathway from KEGG/Reactome with the highest frequency of selected genes with growth phenotype (see also Supplementary Table 10

^e^Variant level classification (Supplementary Tables 1-4): III—Variant of unknown significance, IV—likely pathogenic, V—pathogenic

### Previously reported candidate genes

Of the 63 candidate genes we identified, 6 (9.5%) were previously found to be associated with short stature or syndromes featuring short stature (Table 3 and Supplementary Table 12). A de novo loss of the start codon in *ZBED4* was found in another, smaller exome study in individuals with ISS [14]. A missense variant in *BRD4* was reported to segregate in one family with short stature [20]. Variants in *FZD2* and *LZTR1* were described in individuals with Robinow syndrome-like phenotype (FZD2) [21, 22] and Noonan syndrome (LZTR1), respectively [23, 24]. Recently, variants in *AMMECR1* were observed in individuals with midface hypoplasia, hearing impairment, elliptocytosis, and nephrocalcinosis (OMIM 300990) [25, 26]. Here, short stature is a constant feature. Biallelic variants in *IFT81* underlie a severe form of short stature (short-rib thoracic dysplasia 19; OMIM 617895). Interestingly, a heterozygous missense variant segregates with the growth deficit in one family, suggesting autosomal dominant inheritance.

**Table 3:** Previously reported short stature associated candidate genes

Gene	Candidate Gene confidence level	No. pats.	Variant level classification^a^			Height	Phenotype		
							Propoportionate	Syndromic	Main features
			V	IV	III				
IFT81	high	2		2		−2.6 & −2.9	2/2	0/2	Wide nasel bridge, high arched eye brows
AMMECR1	medium	1	1			−3.2	1/1	0/1	Lacrimal duct aplasia
BRD4	medium	1		1		−2.9	1/1	1/1	Short neck, low set reas, sparse eyebrows, frontal bossing
FZD2	medium	2		1	1	−2.9 & −4.2	2/2	2/2	Posteriorly rotated ears, abnormalities of the eye brows
LZTR1	medium	2	1	1		−3.2 & −3.0	1/2	1/2	none
ZBED4	medium	1	1			−2.6	1/1	0/1	Brachydactyly, broad philtrum, low set ears

^a^Variant level classification (Supplementary Tables 1-4): III—Variant of unknown significance, IV—likely pathogenic, V—pathogenic

### Enrichment analysis and clustering of genes known to be related to growth retardation

1025 genes potentially involved in growth delay or growth regulation were selected using the keywords “growth delay” from the Human Phenotype Ontology (HPO) database and “short stature” from OMIM and MedGen (Supplementary methods). Based on their significantly enriched functions, pathways and other biological features, we identified 29 clusters, including skeletal system development (GO:0001501), appendage development (GO:0048736), and ciliopathy and metabolic processes (GO:0044710) (Fig. 1b). Moreover, clusters were often related to central processes like chromatin binding (GO:0003682) or extracellular matrix (GO:0310122).

Enrichment analysis revealed that 95% of the candidate genes were mapped to 26 of the 29 clusters previously implicated in growth or short stature (Fig. 1g and Table 4). *LAMA5* and *MED24* were not mapped to any of these 29 clusters (Table 4 and Supplementary Tables 7, 10, and 11). The main involved KEGG pathways for the 13 high-confidence candidate genes were: *metabolic pathways* (hsa:01100), *PI3K-Akt signaling pathway* (hsa:04151), *signaling pathways regulating pluripotency of stem cells* (hsa:04550), *thyroid hormone signaling pathway* (hsa:04919), and *pathways in cancer* (hsa:05200) (Table 2 and Supplementary Table 10).

**Table 4:** Results of gene cluster analysis and functional distribution growth associated genes and candidate genes

Cluster name	Growth associated genes		High-confidence candidate genes			Medium-confidence candidate genes	
	No.	Mean fold-enrichment	No.	Mean fold-enrichment	Name	No.	Mean fold-enrichment
Skeletal system development	996	3.2	11	0.7	*CPZ, EDEM3, FBRS, IFT81, KCND1, PLXNA3, RASA3, SLC7A8, UBR4, USP45, ZFHX3*	49	0.9
Appendage development	894	2.8	9	0.5	*CPZ, EDEM3, FBRS, IFT81, KCND1, PLXNA3, RASA3, SLC7A8, ZFHX3*	32	1.4
Ciliopathy	845	3.6	9	1.5	*EDEM3, IFT81, KCND1, PLXNA3, RASA3, SLC7A8, UBR4, USP45, ZFHX3*	38	1.2
Single-organism metabolic process	740	3.7	5	0.1	*CPZ, EDEM3, KCND1, RASA3, SLC7A8*	28	0.6
Catalytic activity	721	1.7	6	0.2	*CPZ, EDEM3, RASA3, UBR4, USP45, ZFHX3*	33	1.8
Chemical homeostasis	629	1.9	5	1.0	*EDEM3, IFT81, KCND1, RASA3, SLC7A8*	22	0.8
Extracellular matrix	521	1.4	5	1.1	*CPZ, EDEM3, FBRS, PLXNA3, SLC7A8*	23	0.9
Peroxisome biogenesis disorder	361	6.0	2	0.1	*IFT81, SLC7A8*	9	0.3
Ossification	284	5.6	2	0.4	*CPZ, EDEM3*	5	1.6
Chromatin binding	265	2.9	2	0.2	*PLXNA3, ZFHX3*	12	0.8
Proteoglycan metabolic process	196	3.0	1	0.5	*EDEM3*	9	0.8
Oxidoreductase activity	190	2.7				2	0.1
Congenital hypothyroidism	184	4.2					
Diamond-Blackfan anemia	182	3.5				5	0.4
Striated muscle tissue development	180	2.5	2	0.9	*KCND1, RASA3*	3	0.4
Single-organism carbohydrate catabolic process	155	2.4				4	1.8
DNA repair	141	4.4	2	0.6		4	2.5
Ichthyosis	139	3.9	3	0.5	*EDEM3*	2	0.6
Head development	125	2.8	2	2.8	*PLXNA3, ZFHX3*	2	0.3
Hydrolase activity. acting on ester bonds	116	1.9	1	1.6	*EDEM3*	1	0.1
Behavior	105	2.3	1	0.3	*ZFHX3*	1	0.2
Sensory organ development	101	4.0				2	4.3
Anatomical structure homeostasis	99	3.1					
Sulfur compound metabolic process	83	3.5				2	0.3
Tissue remodeling	83	2.4	1	0.7	*RASA3*		
Positive regulation of epithelial cell proliferation	78	3.2	1	1.5	*PLXNA3*	1	0.4
Response to nutrient levels	63	3.4				1	0.3
Bone morphogenesis	60	6.3				1	2.4
Reactive oxygen species metabolic process	52	2.6					

### Functional overview of 13 high-confidence candidate genes

Intensive review of the known function of the 13 high-confidence genes led to the localization in 7 functional groups: Wnt signaling (*CPZ*, *IFT81*, *LAMA5*), cellular growth regulation (*EDEM3*, *KCND1*, *SLC7A8*, *UBR4*), thyroid hormone signaling (*CPZ*, *KCND1*, *MED24*), zebrafish phenotype (*PLXNA3*), Ras MAPK signaling (*RASA3*), growth hormone signaling interaction (*ZFHX3*), and ubiquitination (*UBR4*, *USP45*) (Supplementary Table 9). 3 genes are known to be involved in Wnt regulation. *CPZ* is induced by thyroid hormones and modulates Wnt signaling pathways by modification of the activity of Wnt-4 and thereby regulates the terminal differentiation of growth plate chondrocytes. *IFT81* encodes a member of the IFT complex B core. Together with IFT74, IFT81 is required for ciliogenesis. *LAMA5* encodes the laminin subunit alpha 5 and regulates Wnt- and PI3K signaling. Besides CPZ, KCND1 and MED24 have a reported function in thyroid hormone signaling. *KCND1* is expressed in the thyroid gland which might imply a function in the hormonal regulation of growth. *MED24* encodes one of the thyroid hormone receptor-associated proteins, that forms a complex with the thyroid receptor via TRAP220 [27]. 5 high-confidence genes, *EDEM3*, *KCND1*, *SLC7A8*, *UBR4*, *USP45*, are involved in the regulation of cellular growth, either by protein degradation (*EDEM3*), thyroid hormone regulation (*KCND1)*, the mTOR pathway (*SLC7A8*) or ubiquitination (*UBR4*, *USP45*). Furthermore, ZFHX3 interacts with POU1F1, a member of the growth hormone pathway. *RASA3* encodes a Ras-GTPase activating protein and is thus part of the RAS MAPK pathway.

## Discussion

Growth-related disorders constitute a very heterogeneous group of disorders. Based on the results from large GWAS and gene-expression studies, we have estimated that at least 1000 genes are involved [5, 28]. These studies highlighted the observation that rare, large effect-size variants of growth retardation are inherited according to the three classical patterns of Mendelian inheritance [28]. Nevertheless, 67% of individuals with ISS remain without a diagnosis [5]. Using exome sequencing, we therefore aimed to identify novel genes associated with short stature in 254 affected families in whom known causes of short stature had previously been excluded (Table 2, Supplementary Tables 7 and 11, and Supplementary Figure 1) [5]. We identified variants in 63 candidate genes in a total of 92 independent families. Based on a classification scheme using variant and gene information as well as the number of independent affected individuals, we classified 13 genes as high-confidence genes and 50 other genes as medium-confidence candidates (Fig. 1c–e and Supplementary Figure 2).

To facilitate their characterization, we compiled a list of 1025 genes from HPO, OMIM, and MedGen based on the keywords “growth delay” and “short stature” and clustered their significantly enriched functions, pathways and other biological features. This resulted in 29 clusters, the largest of which were skeletal system development, appendage development, metabolic processes, and ciliopathy. These clusters reflect not only processes of skeletal development but also genes involved in basic processes like cellular growth (Fig. 1a, b, Table 4, and Supplementary Figures 5–7). Interestingly, the clusters with the highest mean enrichment were bone morphogenesis (6.3-fold), peroxisome biogenesis disorder (6.0-fold), and ossification (5.6-fold). These results confirmed a broad functional range of potentially growth-related genes. In addition, we generated a functional map of growth-associated genes that provides general information on functions, pathways and other biological features overrepresented in this gene set. We were able to map 95% of the 63 candidate genes onto at least one of the 29 clusters, supporting their relevance to growth (Fig. 1g and Table 4). These include the aforementioned clusters with a high mean enrichment, but also more specific clusters such as extracellular matrix, ossification, bone morphogenesis, and several catalytic processes.

The sensitivity of our approach to identify candidate genes for short stature was further supported by the identification of six genes (*IFT81*, *AMMECR1*, *BRD4*, *FZD2*, *LZTR1*, *ZBED4*) recently found to be associated with this phenotype (Table 3, Supplementary Table 12). Both the previously reported mode of inheritance and the type of variant were observed for *AMMECR1* (X-linked recessive loss of function variants) [25, 26], *BRD4* (autosomal dominant missense variants) [20] and *ZBED4* (de novo nonsense variants) [14]. In addition, the clinical phenotype of the affected individuals was part of the phenotypic spectrum reported, providing additional evidence for their implication (Table 3 and Supplementary Table 12). Interestingly, while nonsense variants in *FZD2* cause severe skeletal dysplasia phenotypes [21, 22], we identified missense variants in this gene in individuals with ISS, suggesting that missense variants are associated with a milder phenotype. *IFT81* has previously been shown to be associated with an autosomal recessive syndromic form of short stature [24, 29, 30]. We propose that heterozygous variants affecting only one allele cause only short stature, a mechanism similar to that recently demonstrated for variants in *ACAN* [9]. For *LZTR1*, which is involved in the RAS-Map kinase pathway, the situation is more complex, since variant type-dependent recessive and dominant inheritance modes were observed to cause a Noonan-spectrum disorder with short stature as a consistent phenotype [23, 24]. We hypothesize that specific missense variants may be associated with isolated short stature.

*RASA3* and *FGF18* are two novel candidates implicated in known signaling pathways. In *RASA3*, we identified a de novo frameshift variant leading to a pre-terminal stop codon in one individual, and a de novo missense variants located in the C2 domain in a second individual, suggesting a loss-of-function effect. Through collaboration, we identified one additional individual with ISS and a de novo missense variant in this domain. Molecular modeling revealed that these missense variants potentially interfere with proper function of the C2 domain, which is reported to be relevant in targeting the protein to the cell membrane (Fig. 1f) [31]. Both the disruption of GTPase, mediated by a pre-terminal stop codon, and mislocation in the cell may potentially interfere with its proper function and thus lead to reduced inactivation of Ras-signaling and further hyperactivation of the pathway [32]. Likewise, FGF18 plays an important role in chondrogenesis and osteogenesis by binding to FGFR2 and FGFR3 [33]. Gain-of-function variants in both receptors were reported to cause mainly syndromic forms of short stature [34–36]. We propose that, by analogy with other members of this group of growth factors, a disease causing variant in the interacting protein leads to constitutional activation of the receptors in the complex [37].

Our classification, in addition to variant and gene information, is based on the number of independent affected individuals. All 13 high-confidence candidate genes were identified in at least 2 families, and 5 candidates even exhibited variants in up to 8 families. The most promising candidates were *LAMA5* with 4 variants likely affecting the function and 4 variants of unknown significance in 8 families, and *UBR4* with 5 variants likely affecting the function and 1 variant of unknown significance in 6 families (Table 2). All affected individuals carrying variants in either of these genes presented with proportionate short stature and a mean height that was 2.9 SDS below average. *LAMA5* encodes the laminin subunit alpha 5, which plays a crucial role in development by regulating Wnt and PI3K signaling during osteoblast differentiation [38–40]. Recent reports demonstrated an involvement in syndromes including kidney disease, osteoarthritis and hypothyroidism, and an association with reduced height in elderly individuals from Southern Italy [41–44]. Studies in Lama5 mice with a hypomorphic allele indicated an essential role for *LAMA5* in growth development [45]. Correspondingly, UBR4, the ubiquitin protein ligase E3 component n-recognin 4, is involved in cancer cell growth [46]. In *Ubr4* knockout mice, growth retardation was reported in fetuses [47]. A 3.46 Mb deletion encompassing *UBR4* was reported in an affected individual with short stature [48]. Intolerance to missense variants as indicated by a z-score of 5.98 (ExAC) suggested that the variants identified in the 6 affected individuals are highly likely to be associated with the phenotype [48, 49]. For another 50 genes, we found strong functional evidence for their involvement, albeit some were identified only in a single family. Thus, we cannot exclude that some genes may be unrelated to short stature (Supplementary Table 8-10).

If we were to consider only the 13 high-confidence candidates, this would yield an exome sequencing detection rate of 15% in highly selected families in whom known causes were previously excluded. This probably constitutes an underestimation as we assume that some of the other 50 candidate genes may be confirmed in subsequent studies. We may have missed smaller structural variants, variants in noncoding or insufficiently covered regions, or epigenetic changes, which have also been reported in connection with short stature [4, 50]. Furthermore, polygenic inheritance, mating selection and familial height variability may have hampered the clinical characterization of, and hence the identification of, the underlying variant.

In conclusion, we identified and characterized 13 high-confidence candidate genes with variants in two or more independent families and another 50 medium-confidence candidate genes. Of these candidate genes, 95% are annotated with functions, pathways and other biological features that are significantly enriched among 1,025 growth-associated genes. These results illustrate that in entities with extremely high heterogeneity and complexity, such as growth defects, clinical characterization and variant-level and gene-level information need to be combined to identify candidate genes. Our study also suggests that single gene defects are an important contributor to the extreme lower end of the growth distribution.

## Supplementary information

Supplemental Material

Supplemental Tables

## Data Availability

The datasets generated during and/or analyzed during the current study are available from the corresponding author on reasonable request.
