# Supplementary material for: Evolutionary conserved networks of human height identify multiple Mendelian causes of short stature
Source: Eur J Hum Genet. 2019 Feb 26;27(7):1061–71. doi: 10.1038/s41431-019-0362-0 (PMC6777496; doi:10.1038/s41431-019-0362-0)
Supplement: Supplementary file 1 — Supplemental Material [file 41431_2019_362_MOESM1_ESM.docx]

Supplementary Information

**Evolutionarily conserved networks of human growth identify multiple Mendelian causes of short stature**

N.N. Hauer^1^, B. Popp^1^, L. Taher^2^, C. Vogl^1^, P.S. Dhandapany^3,4^, C. Büttner^1^, S. Uebe^1^, H. Sticht^5^, F. Ferrazzi^1^, A.B. Ekici^1^, A. De Luca^6^, P. Klinger^7^, C. Kraus^1^, C. Zweier^1^, A. Wiesener^1^, R. Abou Jamra^8^, E. Kunstmann^9^, A. Rauch^10^, D. Wieczorek^11, 12^, A.-M. Jung^13^, T.R. Rohrer^13^, M. Zenker^14^, H.-G. Doerr^15^, A. Reis^1^, C.T. Thiel^1^*

^1^Institute of Human Genetics, Friedrich-Alexander-Universität Erlangen-Nürnberg FAU, Erlangen, Germany

^2^Bioinformatics, Department of Biology, Friedrich-Alexander-Universität Erlangen-Nürnberg, Erlangen, Germany.

^3^Centre for Cardiovascular Biology and Disease, Institute for Stem Cell Biology and Regenerative Medicine (inStem), Bangalore, India.

^4^The Knight Cardiovascular Institute, Departments of Medicine, Molecular and Medical Genetics, Oregon Health and Science University, Portland, Oregon, USA.

^5^Institute of Biochemistry, FAU Erlangen-Nürnberg, Erlangen, Germany

^6^ Molecular Genetics Unit, Casa Sollievo della Sofferenza Hospital, IRCCS, San Giovanni Rotondo, Italy

^7^Department of Orthopedic Rheumatology, Friedrich-Alexander-Universität Erlangen-Nürnberg (FAU), Erlangen, Germany

^8^Institute of Human Genetics, University of Leipzig, Leipzig, Germany

^9^Institute of Human Genetics, University of Würzburg, Würzburg, Germany

^10^Institute of Medical Genetics, University of Zurich, Zurich, Switzerland

^11^Institute of Human Genetics, University of Duisburg-Essen, Essen, Germany

^12^Institute of Human-Genetics, Medical Faculty of University Düsseldorf, Düsseldorf, Germany

^13^Division of Pediatric Endocrinology, Department of General Pediatrics and Neonatology, Saarland University Medical Center, Homburg/Saar, Germany.

^14^Institute of Human Genetics, Otto‐von‐Guericke University Magdeburg, Magdeburg, Germany

^15^Department of Pediatrics and Adolescent Medicine, Friedrich-Alexander-Universität Erlangen-Nürnberg FAU, Erlangen, Germany

**Table of Content**

[Supplementary Methods 4](#_Toc520124724)

[Affected individual recruitment and systematic phenotyping 4](#_Toc520124725)

[Exome Sequencing 5](#_Toc520124726)

[Library preparation & Sequencing 5](#_Toc520124727)

[Mapping & Variant calling 5](#_Toc520124728)

[Variant priorization strategy & bioinformatics pipeline 5](#_Toc520124729)

[Variant validation 5](#_Toc520124730)

[Candidate gene identification 6](#_Toc520124731)

[Variant level classification 6](#_Toc520124732)

[Gene level classification 7](#_Toc520124733)

[Combined score 8](#_Toc520124734)

[Candidate gene ranking 8](#_Toc520124735)

[Supplementary Results 10](#_Toc520124736)

[Protein structure analysis 10](#_Toc520124737)

[Supplementary Figures 11](#_Toc520124738)

[Supplementary Fig. 1. Height distribution of included individuals. 11](#_Toc520124739)

[Supplementary Fig. 2. Exome sequencing and variant interpretation workflow. 12](#_Toc520124740)

[Supplementary Fig. 3. Minimum Coverage Distribution of the exome samples. 13](#_Toc520124741)

[Supplementary Fig. 4. Chondrocyte RNA expression cut-off value calculation. 13](#_Toc520124742)

[Supplementary Fig. 5. Results of enrichment analysis and gene cluster of GO-BP terms. 14](#_Toc520124743)

[Supplementary Fig. 6. Results of enrichment analysis and gene cluster of GO-CC terms. 15](#_Toc520124744)

[Supplementary Fig. 7. Results of enrichment analysis and gene cluster of GO-MF terms. 16](#_Toc520124745)

[Supplementary Fig. 8. Structural effect of protein variants in LAMA5 and PLXNA3. 17](#_Toc520124746)

[Supplementary Fig. 6. High-confidence candidate gene *CPZ*. 18](#_Toc520124747)

[Supplementary Fig. 7. High-confidence candidate gene *EDEM3*. 19](#_Toc520124748)

[Supplementary Fig. 8. High-confidence candidate gene *FBRS*. 20](#_Toc520124749)

[Supplementary Fig. 9. High-confidence candidate gene *IFT81*. 21](#_Toc520124750)

[Supplementary Fig. 10. High-confidence candidate gene *KCND1*. 22](#_Toc520124751)

[Supplementary Fig. 11. High-confidence candidate gene *LAMA5*. 24](#_Toc520124752)

[Supplementary Fig. 12. High-confidence candidate gene *MED24*. 25](#_Toc520124753)

[Supplementary Fig. 13. High-confidence candidate gene *PLEXNA3*. 26](#_Toc520124754)

[Supplementary Fig. 14. High-confidence candidate gene *RASA3*. 27](#_Toc520124755)

[Supplementary Fig. 15. High-confidence candidate gene *SLC7A8*. 28](#_Toc520124756)

[Supplementary Fig. 16. High-confidence candidate gene *UBR4*. 30](#_Toc520124757)

[Supplementary Fig. 18. High-confidence candidate gene *ZFHX3*. 32](#_Toc520124758)

[Supplementary Tables 33](#_Toc520124759)

[Supplementary Table 1. Criteria for candidate gene variant scoring of dominant inherited variants 33](#_Toc520124760)

[Supplementary Table 2. Criteria for candidate gene variant scoring of recessive inherited variants 33](#_Toc520124761)

[Supplementary Table 3. Criteria for candidate gene variant scoring of splice site variants 33](#_Toc520124762)

[Supplementary Table 4. Gene level evaluation of candidate genes 34](#_Toc520124763)

[Supplementary Table 5. Candidate gene combined score calculation 38](#_Toc520124764)

[Supplementary Table 6. Candidate gene ranking approach 38](#_Toc520124765)

[Supplementary Table 7. High-confidence candidate genes 39](#_Toc520124766)

[Supplementary Table 8. Overview of mutations in high-confidence candidate genes and affected individuals' phenotypes 41](#_Toc520124767)

[Supplementary Table 9. Brief description of high-confidence candidate genes with respect to short stature 47](#_Toc520124768)

[Supplementary Table 10. KEGG & REACTOME pathways of high-confidence candidate genes 49](#_Toc520124769)

[Supplementary Table 11. Medium-confidence candidate genes 53](#_Toc520124770)

[Supplementary Table 12. Description of previously reported high-confidence & medium-confidence candidate genes 56](#_Toc520124771)

[Supplementary References 58](#_Toc520124772)

Supplementary Methods

Affected individual recruitment and systematic phenotyping

The study was approved by the ethics committee of the Friedrich-Alexander-Universität Erlangen-Nürnberg (FAU). The parents designated legal guardian or the affected individual provided written informed consent. Here, the risks and benefits of a research-based whole-exome analysis were discussed and an option for disclosure of medically actionable incidental findings provided.

We aimed to enroll affected individuals and at least the parents (Trios) in our study. All affected individuals were examined by local medical specialists prior to enrollment. In a standardized approach all were seen by a pediatric endocrinologist and further specialists (ophthalmologist, ENT, gastroenterologist) where applicable. The standard serology included IGF1, IGFBP3, DHEAS, TSH, fT4, and fT3 parameters and growth hormone stimulation tests when prior tests indicated a growth hormone deficiency. Antibody screening for celiac disease was followed by gastroscopy when elevated antibody parameters were detected. Bone age was measured and documented for affected individuals where no catch up growth until age of 4 years and no disproportionate short stature was observed. As part of the standardized approach radiographic evaluation including knee, pelvic, and spine was performed for affected individuals where a skeletal dysplasia was suspected.

For all affected individuals enrolled in this study growth hormone deficiency, thyroid disease, celiac disease, and other potential underlying organic defects of growth retardation have been excluded prior to genetic evaluation. Systematic phenotyping^1^ was performed according to a standardized questionnaire by the same clinician. The standardized questionnaire included an exhaustive examination and documentation of the growth pattern of the affected individual’s history, a family history of at least 3 generations for evaluation of a potential mode of inheritance, all medical issues and assessment of the developmental status. For all affected individuals the ethnic differences in height were attributed and the age correlated standard deviation score (SDS) for comparison calculated. In addition, we included WHO scores for international comparison. The development of the 565 affected individuals in this study were either classified as normal (450 affected individuals) or learning disability (IQ 70-85, 115 affected individuals). Affected individuals with a disproportionate form of short stature received a radiographic evaluation of the spine, pelvis and knee for metaphyseal-, epiphyseal- or spondylo-dysplasia. X-rays were evaluated by the same clinician and discussed in a panel of experts.

The received phenotype information was included in a database in accordance with the human phenotype ontology.^2^ The integrated information was then discussed with a panel of experts in clinical genetics and dysmorphology. The following diagnostic workup includes the common causes of short stature. Further targeted analysis was based on recognizable phenotypic patterns, followed by exome sequencing and analysis of 1025 growth associated genes from HPO, MedGen and OMIM.

Exome Sequencing

Library preparation & Sequencing

We collected blood samples for DNA extraction from 254 affected individuals, and, where available their parents and further family members. All DNA’s passed our quality control and were then enriched using the SureSelect Human All Exon Kits v5 and v6 (Agilent, Santa Clara, CA) and sequenced on a SOLiD 5500 XL (12 affected individuals) (Thermo Fisher Scientific, Waltham, MA, USA) and Illumina HiSeq 2500 instrument (242 affected individuals) (Illumina, San Diego, CA). Image analysis and base calling was performed using the SOLiD and HiSeq instrument control software with default parameters. Where DNA quantitiy was limited whole genome amplification using REPLI-g (Qiagen, Venlo, Netherlands) prior to enrichment and exome sequencing was performed.

Mapping & Variant calling

Mapping and variant calling was performed as previously described in Hauer et al.^3^ We achieved a median minimum coverage of 64x. On average, 90.98 % of the target sequence was covered at least 20x (Supplementary Fig. 3).

Variant priorization strategy & bioinformatics pipeline

Bioinformatics and priorization of variants was performed similar as described in Hauer et al. (bwa 0.7.8-r455, picard 1.111, GATK 3.1-1-g07a4bf8, SNVer 0.5.3, freeBayes 0.9.15-1-g076a2a2, Platypus 0.7.8, Annovar 2015-03-22 15:29:59, samtools 0.9.15-1-g076a2a2).^3^

Variants were assessed for all 254 individuals and family members based on different modes of inheritance (autosomal dominant de novo, autosomal recessive, and X-linked recessive) facilitated by using our in-house Next Generation Sequencing Variant Analyzer tool.

Only Variants called with GATKHap and GATKUG^4; 5^ were analyzed. We included variants which were covered by at least 10 % of the average coverage of the affected individual’s exome and for which at least 5 novel alleles were detected. As our primary goal was the identification of rare variants accounting for Mendelian inherited disorders, we included only variants with a frequency of 10^-3^ or below from the 1000 Genomes Project^6^, the Exome Variant Server (http://evs.gs.washington.edu/EVS/), the Exome Aggregation Consortium (ExAC)^7^ server or 0.12 % in our in-house variant database (1 out of 840 samples without growth retardation) and none called homozygous.

Different cut-off values for the ratio of novel allele to reference allele reads were used. For analyses of homozygous variants, we included variants with at least 80 % novel allele reads. Heterozygous variants were included only when called in at least 20 % and at most 80 % of all reads. For analyses of hemizygous variants, we included only variants on the X-chromosome in males for which the novel allele was called in at least 60 % of all reads. As we consider a stronger impact of protein altering variants, we restricted our analyses to exonic variants and variants in intronic canonical splice sites ranging from -12 to +5. Furthermore, we excluded synonymous variants from our analyses. Considering missense variants, we only included variants with a CADD-value of at least 10 (other variants are considered benign). The remaining variants were inspected with the Integrative Genomics Viewer to exclude sequencing errors in repetitive regions.

We then evaluated all variants within the affected individuals 1-185 according to “Candidate gene identification” to identify novel short stature candidate genes. The acquired genes harboring variants with combined score 2-5 were then analyzed with respect to the observed mode of inheritance in the affected individuals 186-254 (of which exomes of only the affected individuals were available) and further in affected individuals 150-175 and 184-185 for dominant maternal inherited variants as well as in affected individuals 176-185 for dominant paternal inherited variants.

Variant validation

The remaining variants and their segregation were validated by Sanger sequencing as far as possible. Splice site variants have been evaluated using in silico prediction and for high-confidence and medium-confidence candidate genes by rtPCR where RNA available and expression in lymphocytes (Supplementary Table 3).

Candidate gene identification

Variant level evaluation

To identify novel candidate genes for short stature, we first applied a categorization concerning the variant level in the classes pathogenic, likely pathogenic, variants of unknown significance, likely benign and benign based on information from the ExAC database^8^ and CADD values^9^ according to the following criteria (Supplementary Table 1 and 2):

- Pathogenic variants are nonsense variants, short insertions or deletions (indels) resulting in frameshifts.
- Likely pathogenic variants are inframe indels affecting more than one amino acid with a frequency lower than 10^-4^ in ExAC for dominant inheritance and a frequency lower than 10^-3^ in ExAC for recessive inheritance. Furthermore, missense variants with equal frequencies and CADD-scores of at least 15 are categorized as likely pathogenic.
- Variants of unknown significance (VUS) are inframe indels affecting more than one amino acid with a frequency between 10^-3^ and 10^-4^ in ExAC for dominant inheritance as well as inframe indels affecting one amino acid with a frequency lower than 10^-4^ in ExAC for dominant inheritance and a frequency lower than 10^-3^ in ExAC for recessive inheritance. Moreover, variants of unknown significance are missense mutations fulfilling the same frequency criteria as likely pathogenic variants, but showing a CADD score ranging between 10 and 15 as well as dominant missense variants with an ExAC frequency ranging between 10^-3^ and 10^-4^ and a CADD score of at least 15.
- Likely benign variants are inframe indels affecting one amino acid with a frequency between 10^-3^ and 10^-4^ in ExAC for dominant inheritance or missense variants with a frequency between 10^-4^ and 10^-3^ and a CADD score ranging between 10 and 15 or a frequency of at most 10^-4^ and a CADD score lower than 10 for dominant inheritance as well as missense variants with a frequency lower than 10^-3^ and a CADD score lower than 10 for recessive variants.
- Benign variants are variants with ExAC frequencies higher than 10^-3^ or dominant inherited missense variants with a CADD score lower than 10 and an ExAC frequency between 10^-4^ and 10^-3^.

Thereby, variants with a CADD score lower than 10 and an ExAC frequency higher than 10^-3^ have already been excluded by the variant selection criteria. ExAC frequency cutoff values were selected based on the observation that in this database only affected individuals with severe pediatric phenotypes were excluded. No information about the individuals’ height is available. Thus, we applied cutoff values for rare dominant and recessive phenotypes potentially not presenting full penetrance.

For variants in the splice site region (-5 to -1 and +1 to +8), we performed a separate classification based on rtPCR experiments and splice site prediction^10-12^ according to Supplementary Table 3:

- Pathogenic variants are variants affecting the positions -2, -1, +1 or +2 and variants in the surrounding regions (-12 to -5, -3 or +3 to +5) with an effect on splicing confirmed by rtPCR
- Likely pathogenic variants affect the positions -12 to -5, -3 or +3 to +5 and present with a Spidex score higher than 2 or lower than -2.
- Variants of unknown significance (VUS) affect the positions -12 to -5, -3 or +3 to +5 and either have no available Spidex score or present with a Spidex score between -2 and 2 and a dbscSNV_ada or a dbscSNV_rf score higher than 0.6. Moreover, indels in the defined splice site region were classified as variants of unknown significance.
- Likely benign variants affect the positions -12 to -5, -3 or +3 to +5 and present with a Spidex score between -2 and 2 and a dbscSNV_ada and a dbscSNV_rf score lower than 0.6.

Splice site variants in high-confidence or medium-confidence candidate genes which are predicted to be likely pathogenic or variants of unknown significance were validated by rtPCR experiments if RNA was available and the gene was expressed in blood. If no aberrant splicing was observed, the variant was reclassified as “likely benign” and thus excluded from further classification.

Gene level evaluation

In order to further categorize variants in novel genes according to their functional relevance for growth phenotypes, we established a gene level classification considering the following eight categories (Supplementary Table 11). The detailed gene lists can be found in the appendix “Gene level evaluation information”.

*Association studies.* Genes with identified variants were compared to a list of genes located in linkage disequilibrium (LD) to a height associated single nucleotide polymorphism with genome-wide significance observed in genome wide association studies (GWAS).^13-33^

*Data mining/ Literature.* Genes with identified variants were compared to a list of genes that have previously been reported by an exome sequencing study in order to identify candidate genes for short stature.^34^

*Copy number Variants.* Genes with identified variants were compared to genes with a CNV previously reported in an affected individual with short stature (data from Decipher considering search term “short stature”).^35-39^

*Mouse model.* Genes with identified variants were compared to a list of genes of which mouse models result in growth associated phenotypes. This gene list was compiled from entries in the Mouse Genome Database (MGD)^40^ which includes at least one of the following MGI phenotype terms: MP:0009674: decreased birth weight; MP:0009703: decreased birth body size; MP:0005459: decreased percent body fat; MP:0012322: decreased total tissue mass; MP:0001255: decreased body height; MP:0001258: decreased body length; MP:0001262: decreased body weight; MP:0008974: proportional dwarf; MP:0013138: thin body; MP:0003961: decreased lean body mass; MP:0001732: postnatal growth retardation; MP:0001698: decreased embryo size; MP:0001730: embryonic growth arrest; MP:0003984: embryonic growth retardation; MP:0004200: decreased fetal size; MP:0004201: fetal growth retardation; MP:0010865: prenatal growth retardation.

*Zebrafish model.* Genes with identified variants were compared to a list of genes of which zebrafish models result in growth associated phenotypes. This gene list was compiled from entries in The Zebrafish Model Organism Database (ZFIN)^41^ which include the term “growth”.

*Interaction network.* Genes with identified variants were compared to a list of genes that interact with at least one gene associated with growth. To identify these interactions, we searched for all genes interacting with the gene list using STRING DB^42^ (v10.0, high confidence 0.7; all evidence, combined score).

*Expression in chondrocytes & RNA-Seq.* Genes with identified variants were compared to a list of genes, which are expressed in chondrocytes. To compile this list, an RNASeq in chondrocytes was performed. RNA extraction from three chondrocyte samples was performed using the High Pure RNA Isolation Kit (Roche, Basel, CH) according to the manufacturer‘s protocols. Before and during the library preparation the quality of RNA was analyzed using a 2100 Bioanalyzer system (Agilent, Santa Clara, CA, USA). Barcoded RNA sequencing libraries were prepared from 100 ng total RNA using Nugen Ovation Human FFPE RNA-Seq System for small amounts of RNA according to the manufacturer‘s instructions (Nugen, San Carlos, CA, USA). For selective depletion of rRNAs, the insert-dependent adapter cleavage method from Nugen was used prior to cDNA synthesis (InDA-C). To control sources of variability during sample and data processing, a common set of external spike-in RNA controls, developed by the external RNA controls consortium (ERCC)^43; 44^, was used (Ambion, Foster City, CA, USA).

Libraries were subjected to single-end sequencing (101 bp) on a HiSeq-2500 platform (Illumina, San Diego, CA, USA). Reads mapping to, e.g., rRNAs, tRNAs, snRNAs and interspersed repeats were first filtered out by performing alignment against a manually curated filter reference file using bwa-mem v. 0.7.8-r455^45^ and keeping only unmapped reads. Subsequently, these reads were mapped against the hg19 reference genome using the STAR aligner v. 2.4.0j^46^ and a STAR genome directory created by supplying an Ensembl gtf annotation file (version 2013-09) for hg19.

Absolute read counts per gene were produced using Subread’s featureCounts program v. 1.4.6-p2^47^ and the Ensembl gtf annotation file. Following analyses were performed using R version 3.2.1^48^. First the 23,840 targets assayed by exome analyses were extracted. Then, RPKM (read counts normalized per kilobase of feature length per million mapped fragments) were calculated using gene lengths provided by featureCounts and a robust estimate of library size *(Anders and Huber ^49^,* as implemented in DESeq2 package v.1.8.1).

In order to decide on a cutoff value for the expression values of this RNA-Seq, we looked at their density distribution. As the expression values are composed of those from expressed as well as unexpressed genes, the density will always be composed of a more or less symmetric distribution for the expressed genes and a large “shoulder” on the left side of the graph, representing the unexpressed genes. By maximum likelihood estimation, we determined that a logistic distribution with scale = 0.3615 and location = 0.6264 would most closely fit the right part of our distribution, which represents the expressed genes (Supplementary Fig. 4). We placed our cutoff threshold at the first quartile of this distribution (0.22929), which corresponds to an expression value of 1.69547.

*Gene ontology category.* Genes with identified variants were compared to a list of genes to which general GO-terms for growth phenotypes apply. In order to generate this list, we generated a list of significant enriched (p value 0.05) GO terms and the corresponding genes for the genes associated with growth by usage of DAVID.^50-53^

Details of all parameters are listed in the file “Additional Data Tables.xlsx”.

Combined score

The variant and the gene level classification for each variant was then joined in the combined score according to the scheme in Supplementary Table 5.

Genes harboring only variants with a combined score of 0 or 1 were not considered as candidate genes. For compound heterozygous inherited variants, both variants were excluded from the list of candidate variants if one of them was scored with a combined score of 0 or 1.

Candidate gene ranking

For our final candidate gene ranking, we utilized our combined scores for each variant as well as information about whether variants within one gene could be detected in more than one individual including the information whether segregation of the variant could be verified by sanger sequencing. An overview of this ranking procedure is shown in Supplementary Table 17.

High-confidence candidate genes are genes for which we could identify variants with at least a combined score 3 in at least 2 individuals with verified segregation. In case of compound heterozygous variants, both variants have to be scored with at least a combined score 3.

Medium-confidence candidate genes are genes for which we could identify variants with a combined score lower than 3 in at least 2 individuals with verified segregation, variants with at least a combined score 3 in at least 2 individuals whereby segregation could only be verified in one of them or with a combined score of at least 4 in a single individual.

Likely candidate genes are genes for which we could identify variants with a combined score lower than 3 in at least 2 individuals whereby segregation could only be verified in one of them or for which we could identify variants with a combined score lower than 4 in only one affected individual.

For all variants of unknown significance affecting high-confidence genes, we performed protein modeling or structural analyses if applicable. If an effect on the protein function was indicated, we adjusted the variant’s scoring (from “unknown significance” to “likely pathogenic”).

**Pathway analyses**

The main functional category for each high-confidence gene was assessed on data from KEGG pathways and where no entry in KEGG was available from REACTOME. Functional enrichment analysis was performed using the Database for Annotation, Visualization, and Integrated Discovery (DAVID, Version 6.8,^50; 51^ API services^54^). We analysed whether known growth retardation genes participate in these pathways and identified the pathway with the highest percentage of growth retardation genes as the main pathway for the respective candidate gene (Supplementary Table 10). A false discovery rate (FDR) of 0.05 by the Benjamini and Hochberg approach^55^ was used to determine significant enrichment using all human gene Ensembl identifiers in the BioMart database^56^ as the background. The odds ratio was calculated by dividing the odds from (i) by the odds from (ii) using the fisher.test() function implemented in the R environment for statistical computing.^48^

**Protein modeling and structural analyses**

The Asp82Glu and Val85Ala variants of RASA3 were modeled using the crystal structure of the C2-domain from Protein Kinase C beta II as a template (PDB: 3PFQ).^57^ The effect of the Asn417Tyr variant in LAMA5 was inspected based on a crystal structure that is available for the murine ortholog of this domain (PDB: 2Y38).^58^ The Ala2076Ser variant of LAMA5 was modeled based on the EGF-domain from Human Notch-1 (PDB: 2VJ3).^59^ Thr3441Met and Ser3478Asp are both located in the same domain of LAMA5 and were modeled based on the structure of the mouse laminin alpha chain (PDB: 2JD4).^60^ The PLXNA3 variant Gln451His was modeled using the structure of mouse plexin A2 as a template (PDB: 3OKT).^61^ In the first step, models for all wildtype protein domains were either obtained from Modbase^62^ or modelled using HHpred^63^ and Modeller^64^. In the second step, the variant amino acid was exchanged using Swiss-Model.^65^ Finally, RasMol was used for structure analysis and visualization.^66^ The analysis of signal peptides and phosphorylation sites was performed with SignalP^67^ and PhosphoSitePlus^68^, respectively.

Supplementary Results

Protein structure analysis

There are four VUS in *LAMA5*, which are either located in EGF-domains (residues 359-428, residues 2070-2116), or in the Laminin G-like domain 4 (residues 3340-3513). Three of the variants (Asn417Tyr, Ala2076Ser, Ser3478Asn) are located in solvent-exposed loops of the respective domains. Modeling of the variants did not result in significant steric problems or loss of favorable interactions. Thus, modeling suggests that these variants have only a minor impact on protein structure and thus remain with their classification as VUS. In contrast, the methyl group of Thr3441 forms tight sidechain interactions with Gln3418 that are expected to stabilize the fold of the Laminin G-like domain 4 (Supplementary Fig. 8A). This interaction can only be formed by branched amino acids sidechains and is consequently absent in the The3441Met variant, which exhibits a long unbranched sidechain (Supplementary Fig. 8B). Thus, this variant is expected to cause domain destabilization and is thus further classified as likely pathogenic.

One of the two VUS in PLXNA3 (Gln541His) is located in the globular part of the protein. In the wildtype, Gln541 interacts with Arg543, which is located in the same β-sheet (Supplementary Fig. 8C). A replacement by histidine results in electrostatic repulsion between His541 and Arg543 (Supplementary Fig. 8D), which is expected to destabilize the secondary structure of the domain. Thus, PLXNA3 (Gln541His) is reclassified as a likely pathogenic variant. The second VUS in PLXNA3 (Gly19Asp) is situated in the nonglobular N-terminus of PLXNA3. Gly19 is predicted to be placed exactly at the cleavage site of the signal peptide, which comprises residues 1-19. The Gly19Asp variant is predicted to represent a poorer substrate for cleavage; in addition, the preferential cleavage site is shifted by two residues towards the N-terminus (i.e. between residues 17 and 18). Thus, the variant is expected to affect proper processing of PLXNA3 and is thus reclassified as a likely pathogenic variant.

The remaining VUS, which are predicted to be located in nonglobular parts of the protein structure, were analyzed for the presence of sequence motifs mediating protein interaction or posttranslational modification. The Ser523Gly variant of USP45 is located in a serine-rich sequence stretch, which becomes phsophorylated in the murine ortholog at position Ser525. Consequently, this variant is reclassified as likely pathogenic. The Arg763Gln variant of MED24 is located in proximity to an LXXLL motif (residues 788-792) characteristic for coactivators of nuclear receptors.^69^ Consequently, also Arg763Gln in MED24 is reclassified as likely pathogenic. For the remaining VUS (Pro345Leu in FBRS, Pro4746Leu in UBR4, Glu2850Lys in ZFHX3) structural analysis did not reveal any hints on the effects of those variants so that they remain classified as VUS.

Supplementary Figures

Supplementary Fig. 1. Height distribution of included individuals. The height of each individual is presented as standard deviation (SD) for age and sex based on the last measurement of each individual. No significant difference in height SDS is observed for samples with mutations in candidate genes compared to high-confidence or medium-confidence candidate gene.


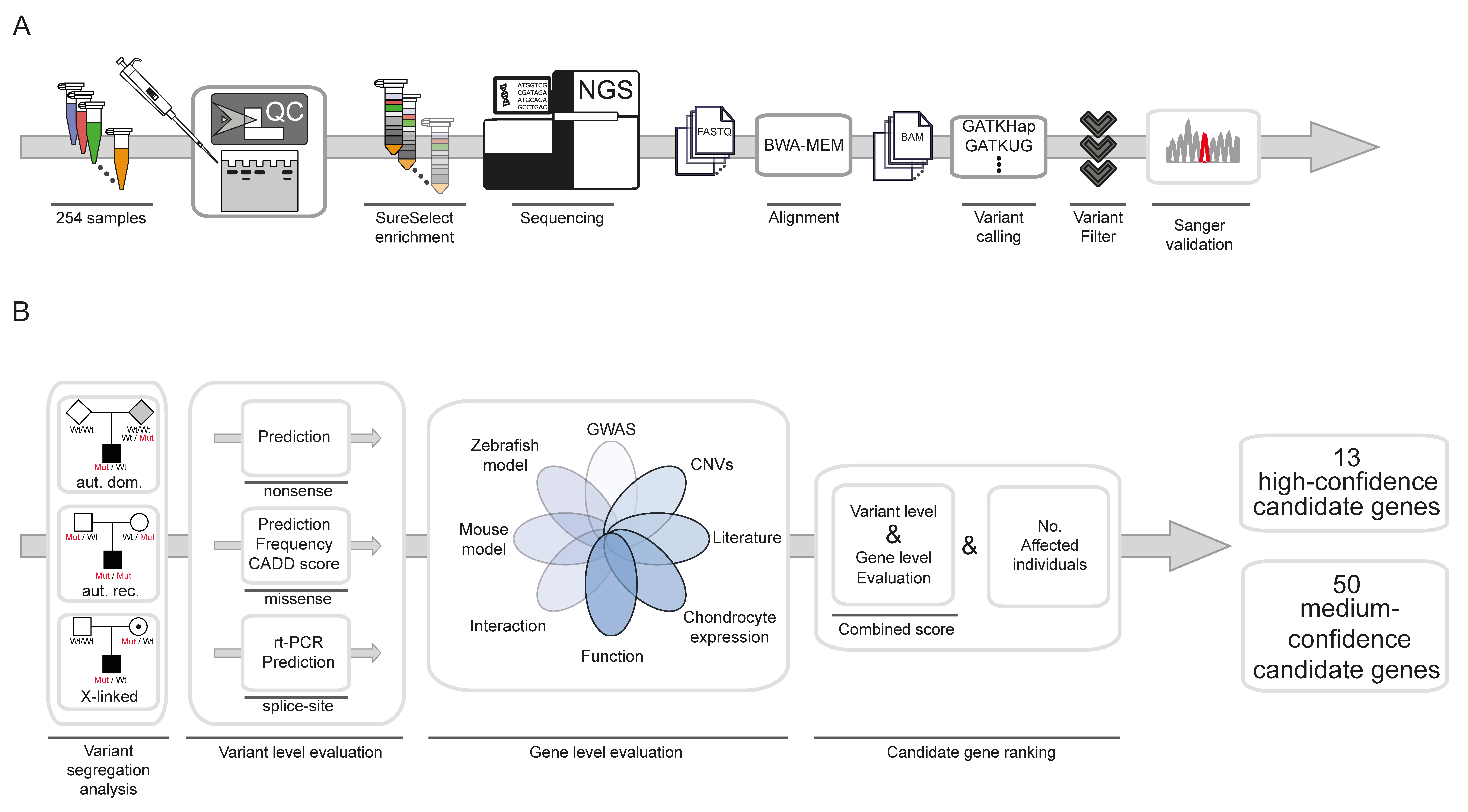


Supplementary Fig. 2. Exome sequencing and variant interpretation workflow. (A) Schematic representation of the exome sequencing procedure. (B) Variant interpretation and candidate gene identification.


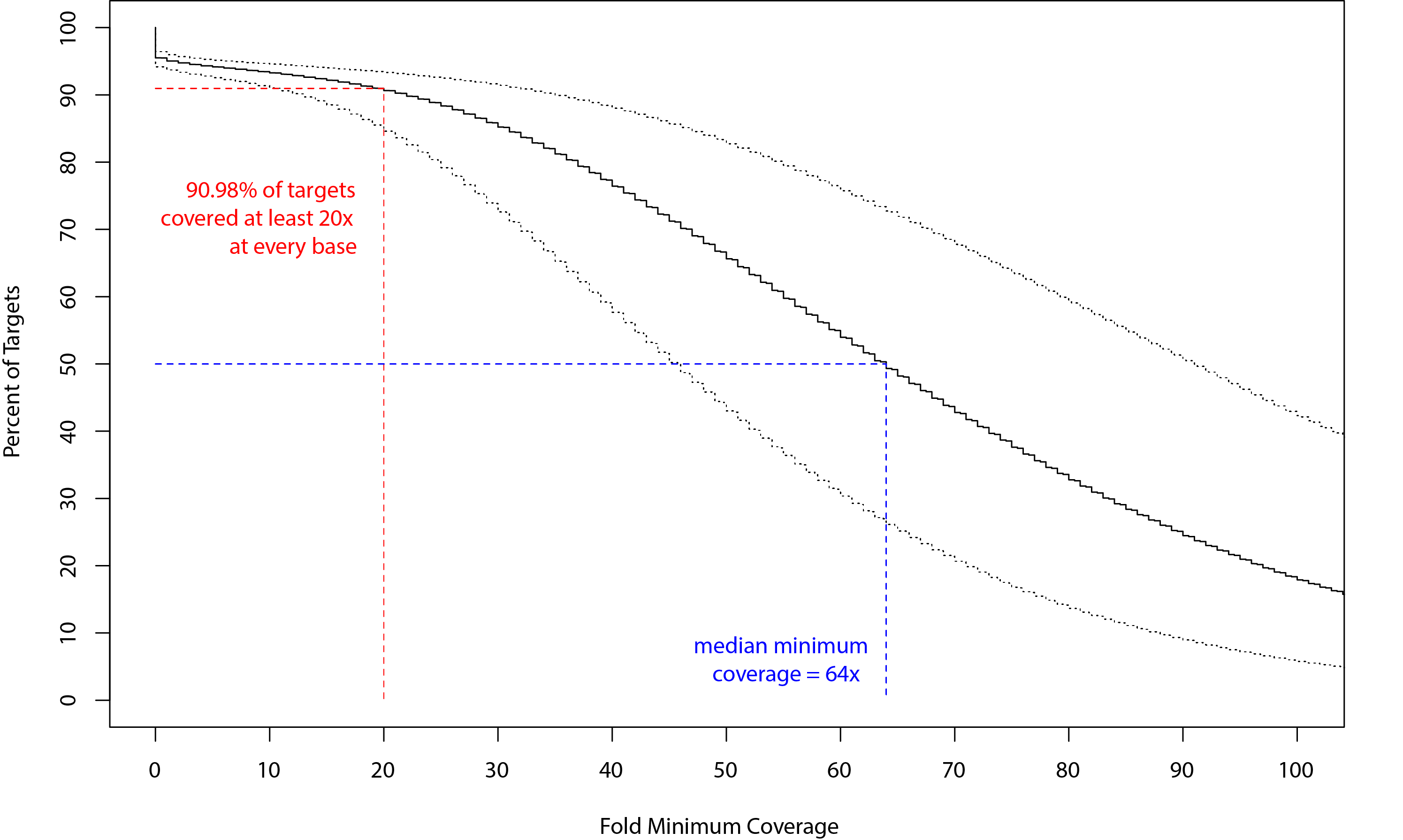


Supplementary Fig. 3. Minimum Coverage Distribution of the exome samples. For every CDS feature according to GRCh37 RefSeq, extended by 5 bp in either direction, minimum coverage, i.e. coverage of the least covered base within the feature, was determined in each of the 624 exomes. Median minimum coverage over all exomes is plotted for every CDS feature; features are sorted by minimum coverage and the percentage of all features with at least this minimum coverage indicated on the y axis. Dotted lines show the 25th and 75th percentile over all exomes for each feature.

**
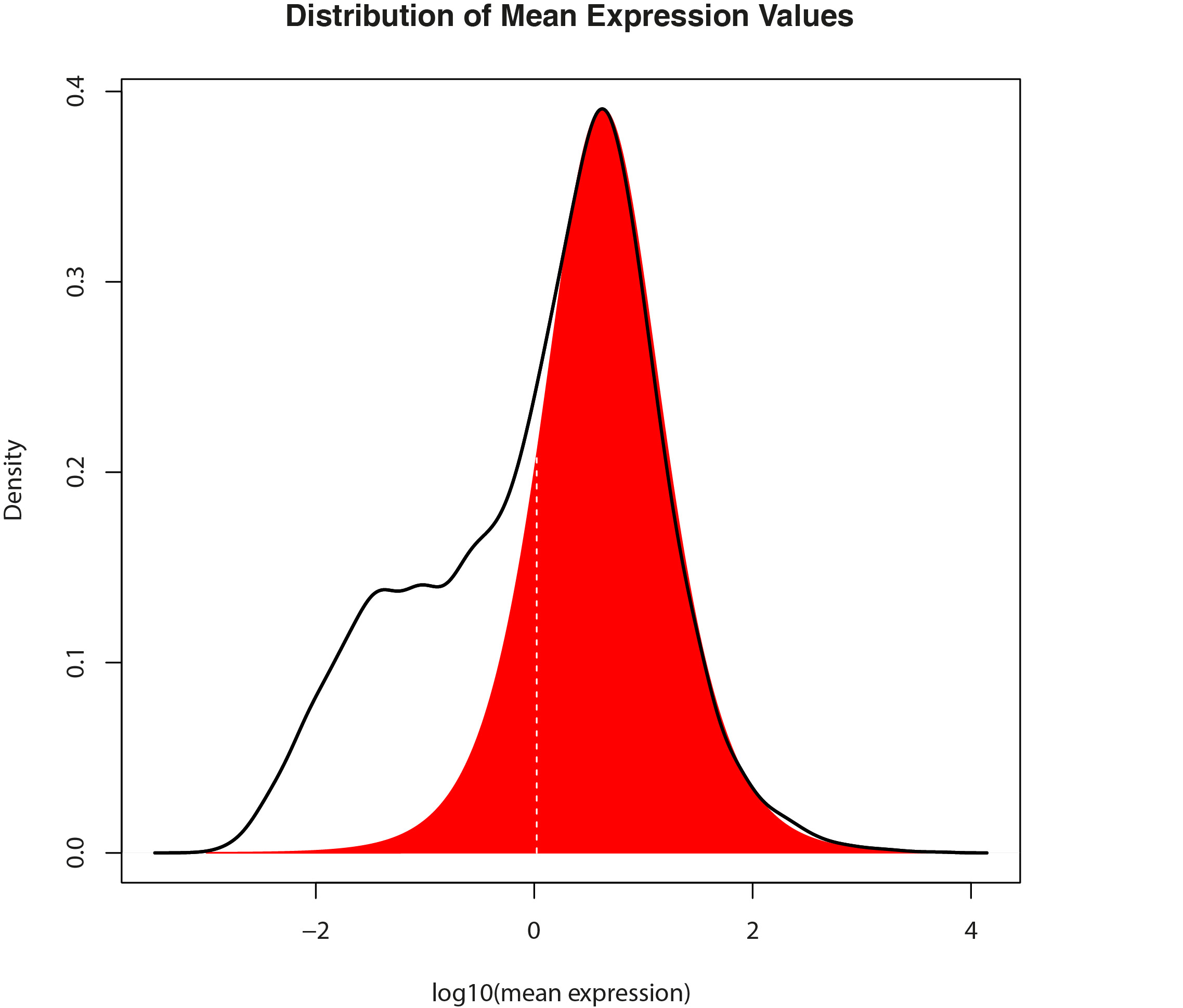
**

Supplementary Fig. 4. Chondrocyte RNA expression cut-off value calculation. Density plot of the decadic logarithm of mean expression values (black line). A logistic distribution with scale = 0.3615 and location = 0.6264 (red area) closely fits the right part of the expression value distribution.








**Supplementary Fig. 5. Results of e**nrichment analysis and gene cluster of GO-BP terms.








**Supplementary Fig. 6. Results of e**nrichment analysis and gene cluster of GO-CC terms.








**Supplementary Fig. 7. Results of e**nrichment analysis and gene cluster of GO-MF terms.


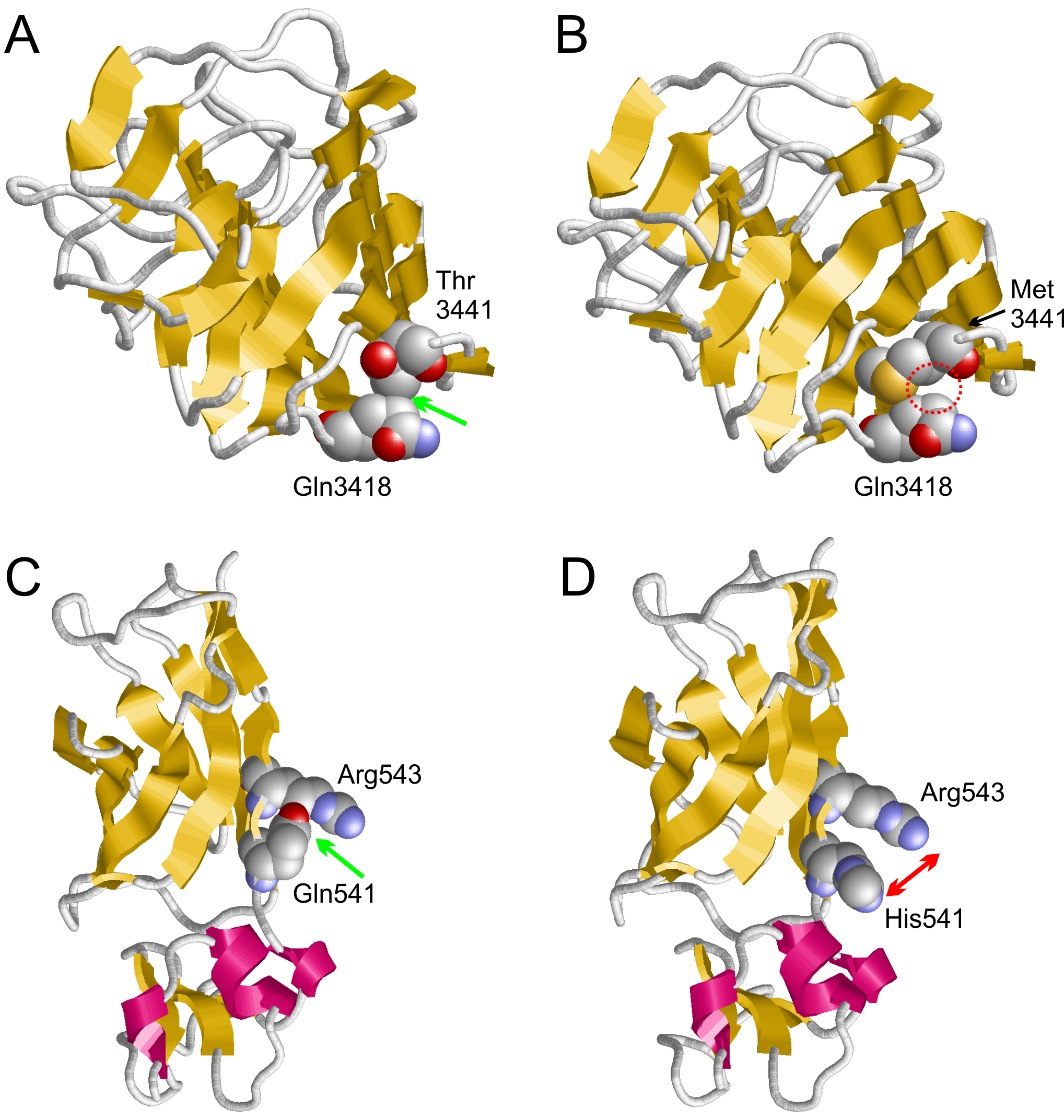


Supplementary Fig. 8. Structural effect of protein variants in LAMA5 and PLXNA3. (A,B) Model of the LAMA5 Laminin G-like domain 4 showing the site of the Thr3441Met variant. (A) The methyl group of Thr3441 forms tight sidechain interactions with Gln3418 (green arrow). (B) These interactions cannot be formed by Met3441 in the variant, because the methionine sidechain lacks a methyl group at the respective position (red dotted circle). (C,D) Model of the PLXNA3 domain harboring the Gln541His mutation. (C) Gln541 form polar interactions with the adjacent Arg543 (green arrow). (D) The presence of a histidine in the Gln541His variant causes electrostatic repulsion between the positively charged sidechains of His541 and Arg543 (red arrow).


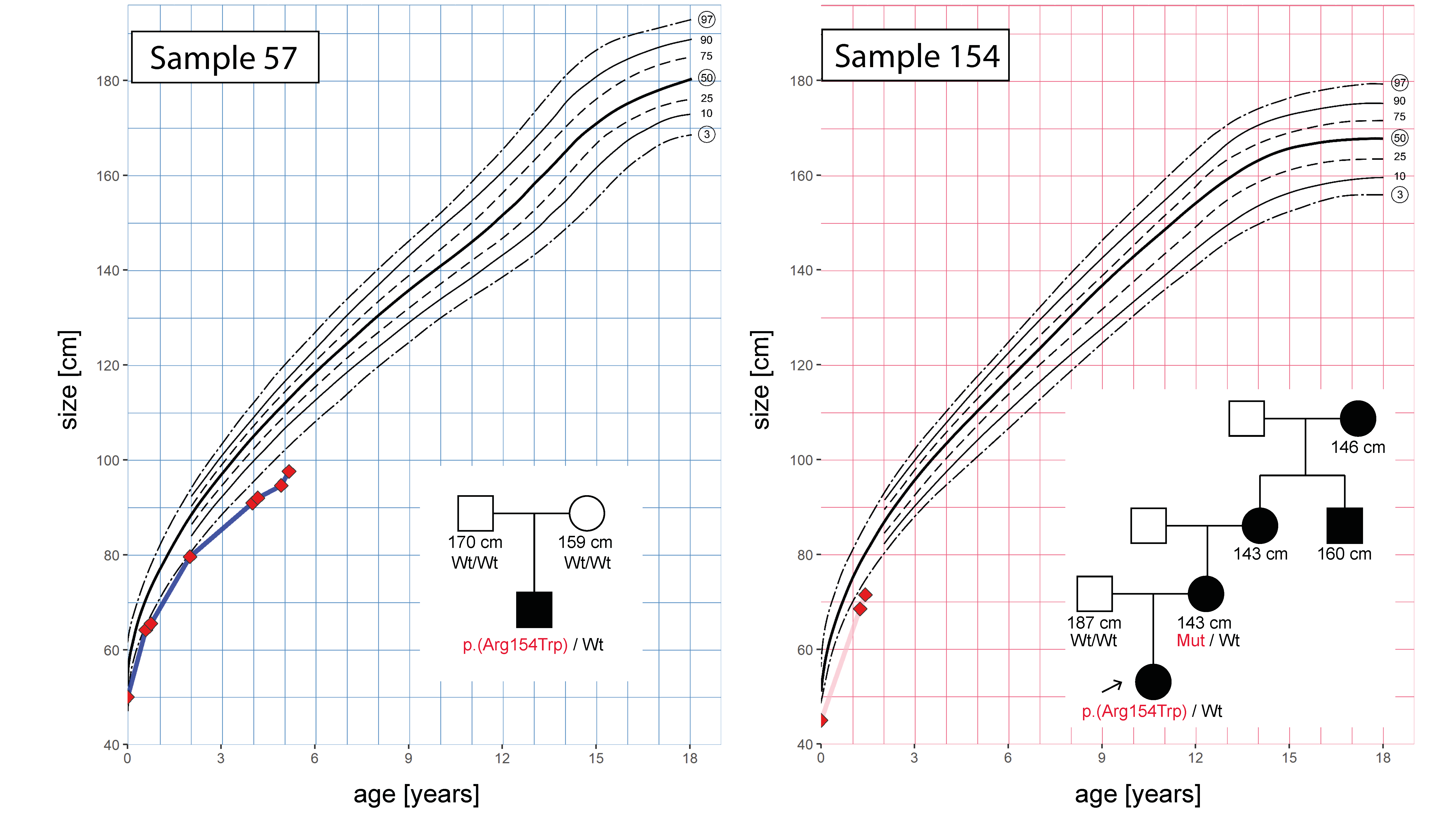


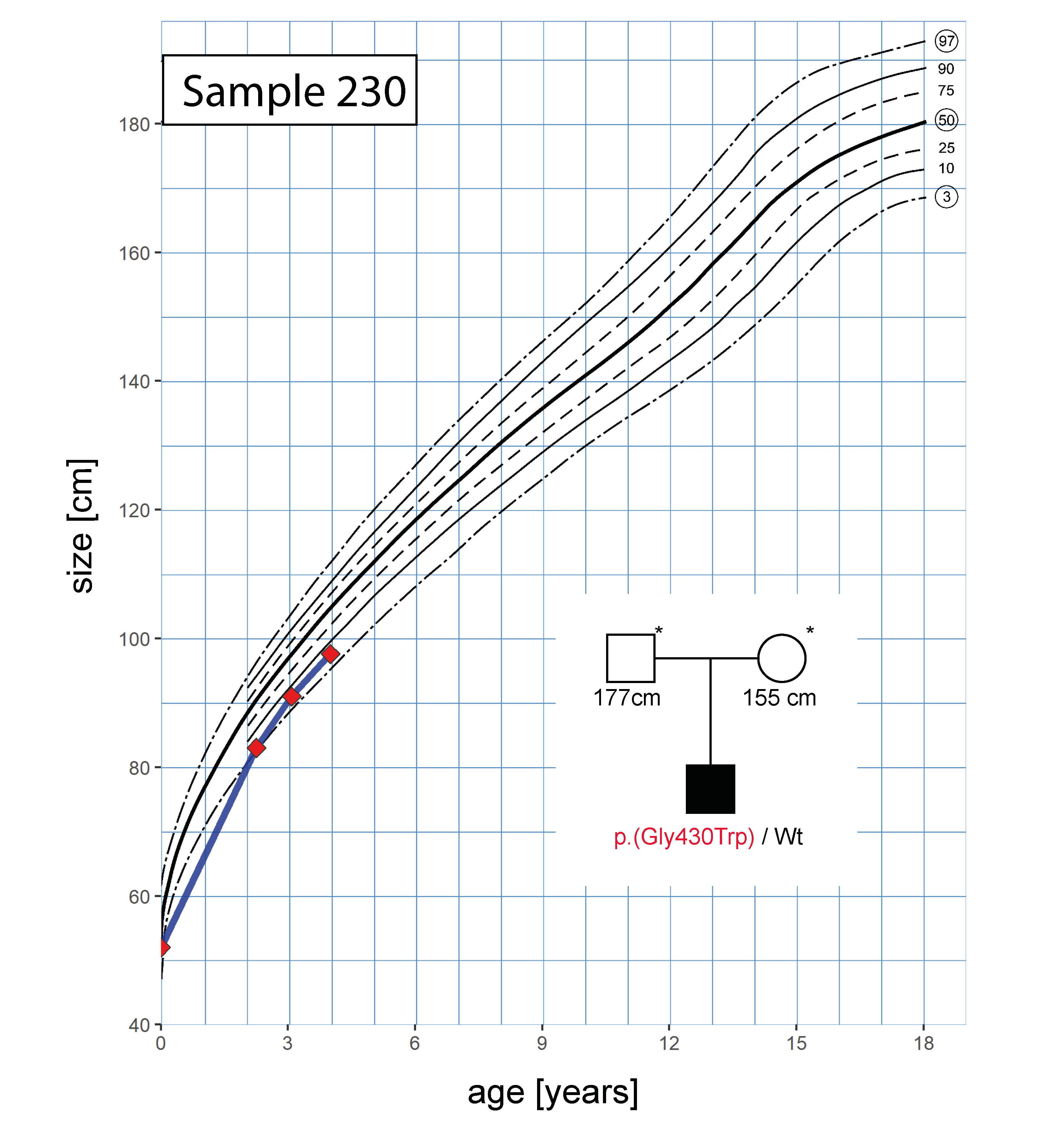


Supplementary Fig. 6. High-confidence candidate gene *CPZ*. Growth charts and pedigrees of affected individuals. Standard growth curves were derived from Reinken et al.^70^ Embedded pedigrees depict the observed mode of inheritance (* parental DNA not available).


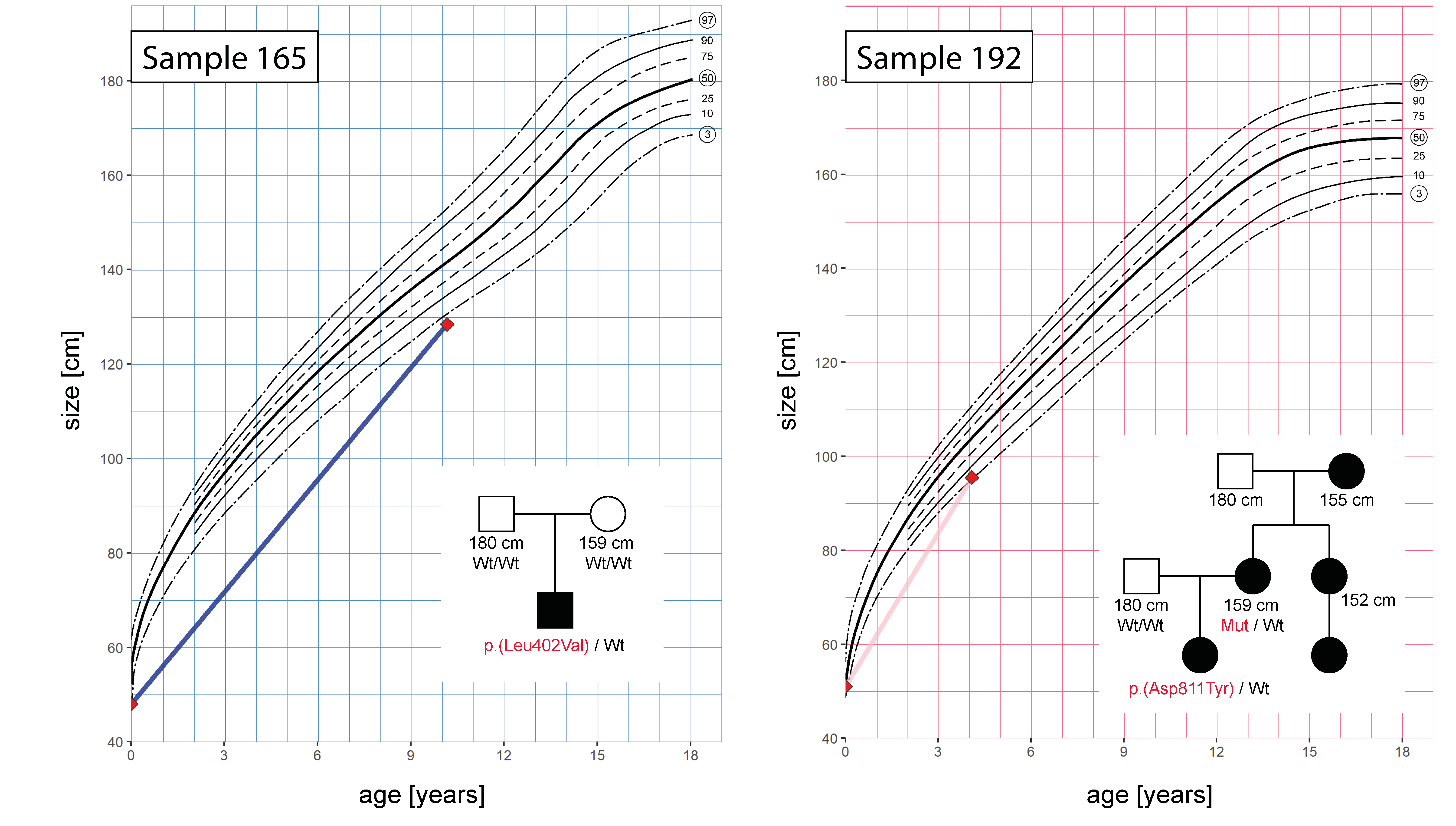


Supplementary Fig. 7. High-confidence candidate gene *EDEM3*. Growth charts and pedigrees of affected individuals. Standard growth curves were derived from Reinken et al.^70^ Embedded pedigrees depict the observed mode of inheritance.


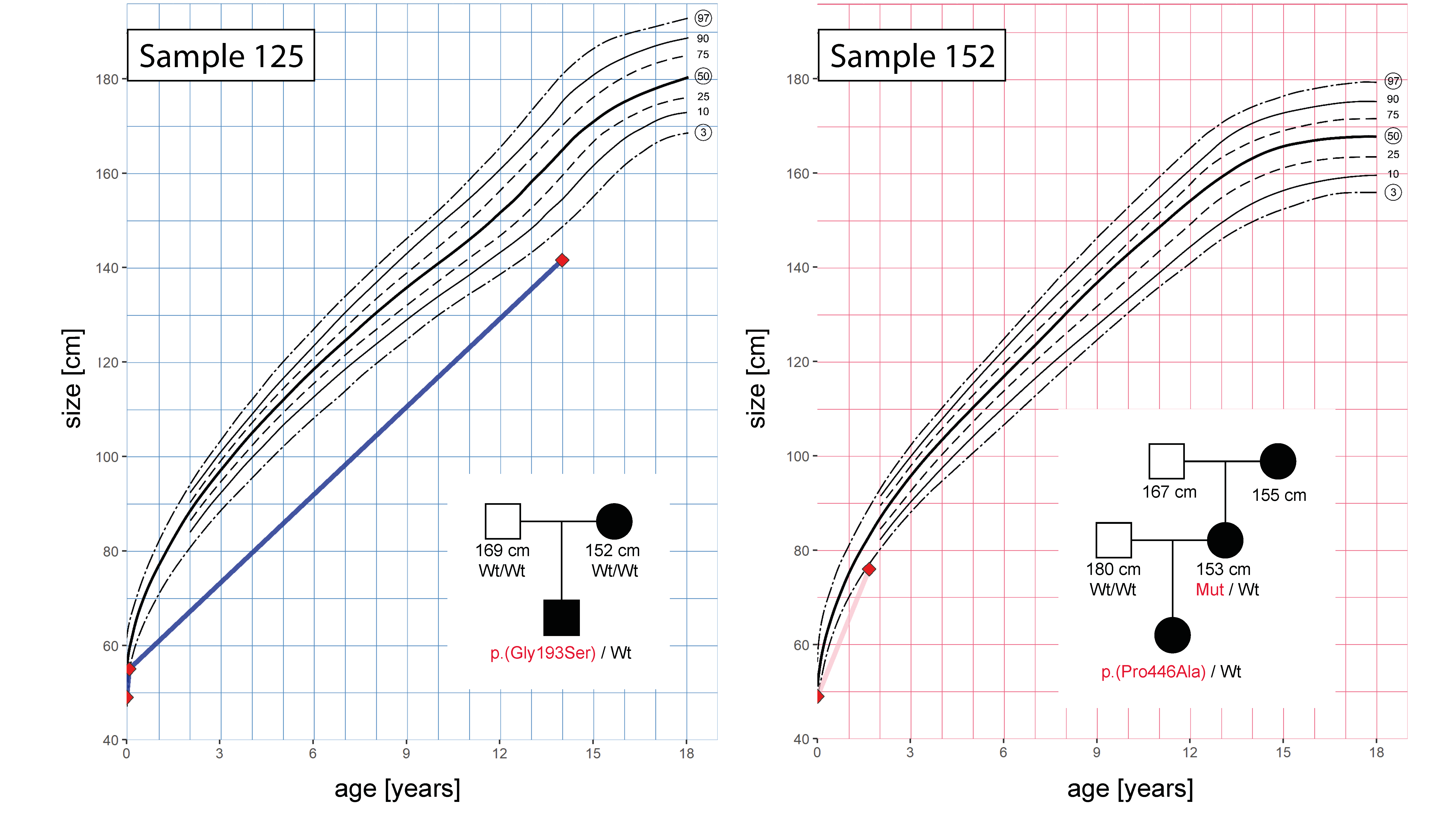


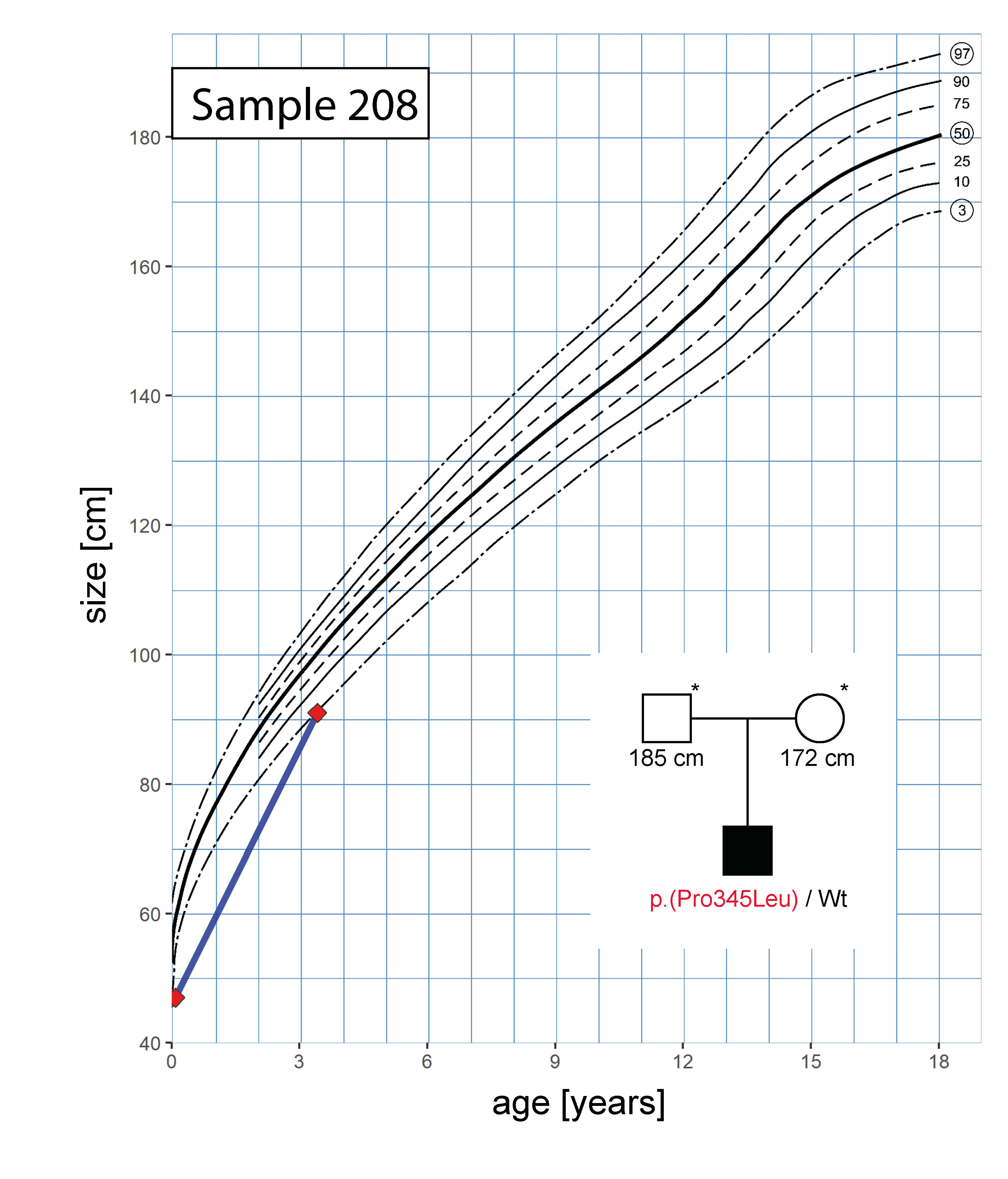


Supplementary Fig. 8. High-confidence candidate gene *FBRS*. Growth charts and pedigrees of affected individuals. Standard growth curves were derived from Reinken et al.^70^ Embedded pedigrees depict the observed mode of inheritance (* parental DNA not available).


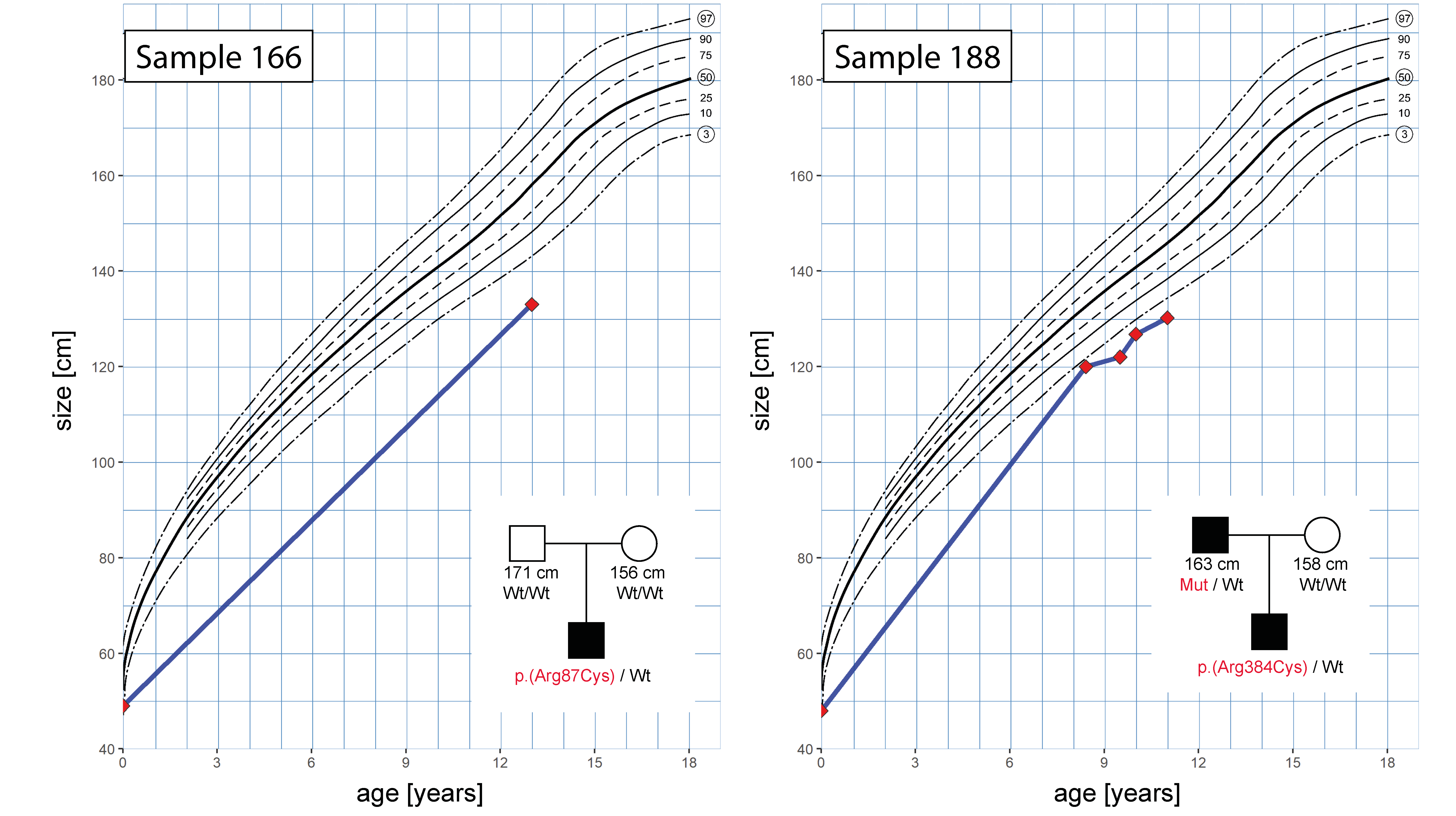


Supplementary Fig. 9. High-confidence candidate gene *IFT81*. Growth charts and pedigrees of affected individuals. Standard growth curves were derived from Reinken et al.^70^ Embedded pedigrees depict the observed mode of inheritance.


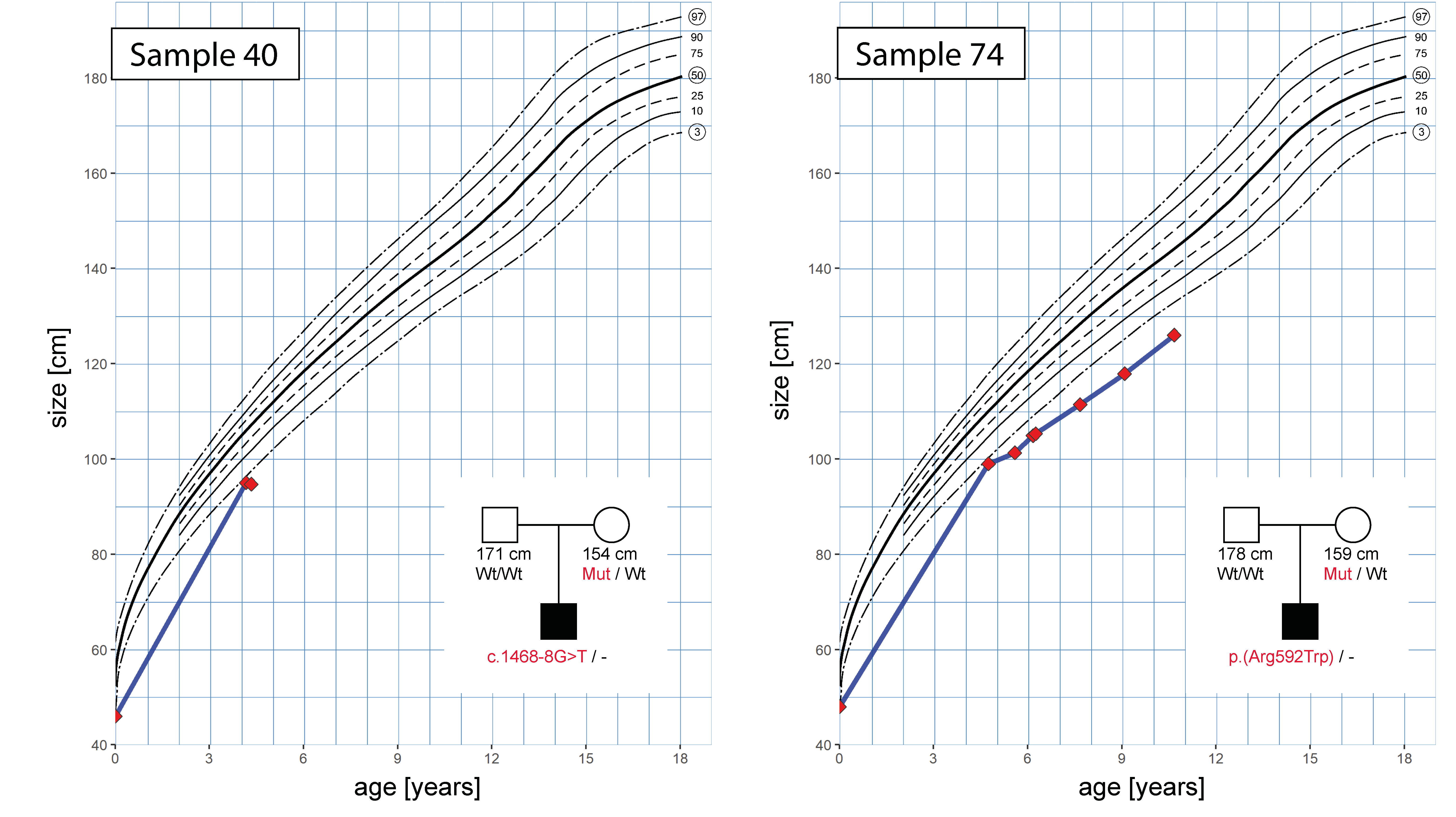


Supplementary Fig. 10. High-confidence candidate gene *KCND1*. (Growth charts and pedigrees of affected individuals. Standard growth curves were derived from Reinken et al.^70^ Embedded pedigrees depict the observed mode of inheritance.


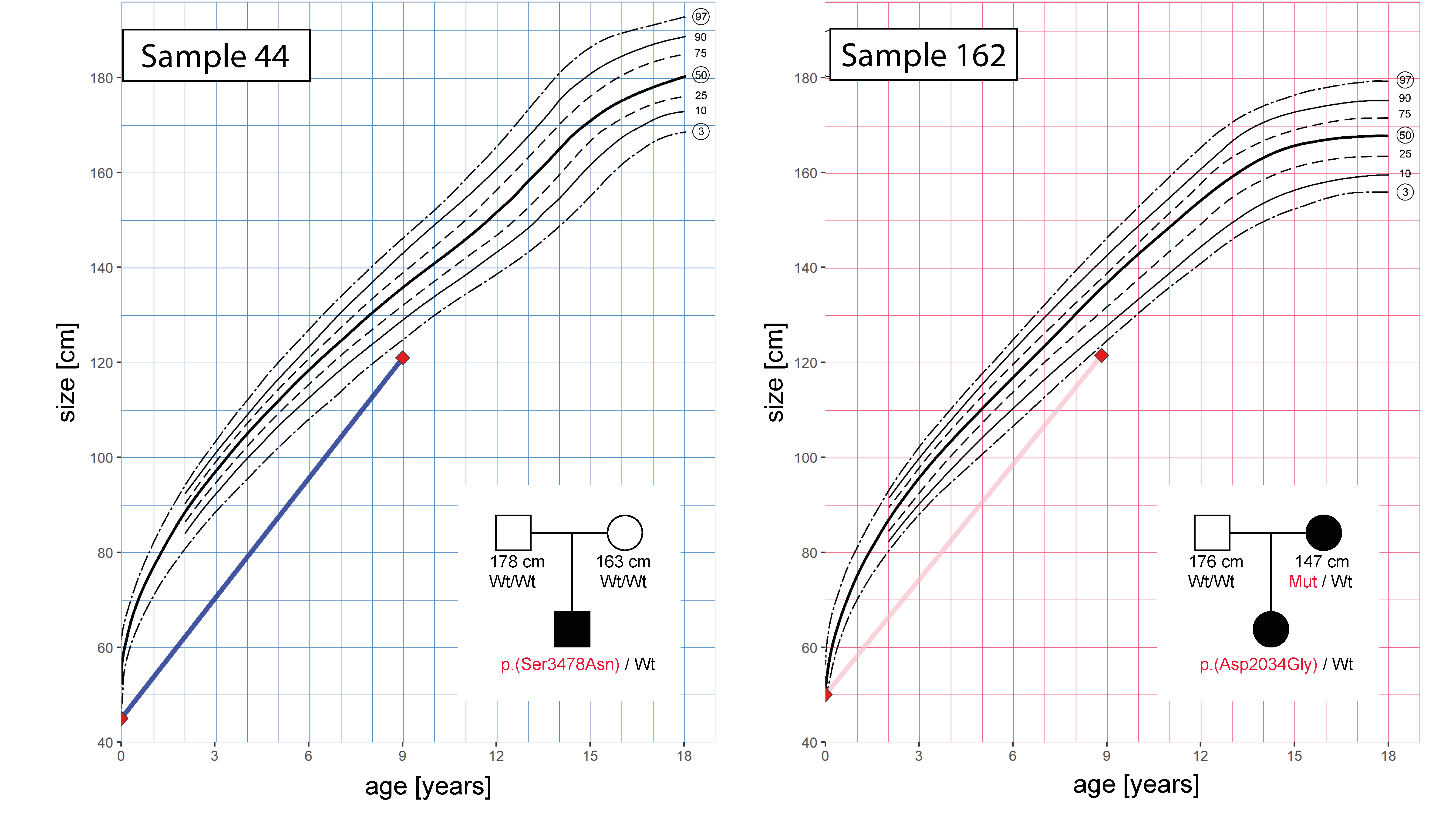


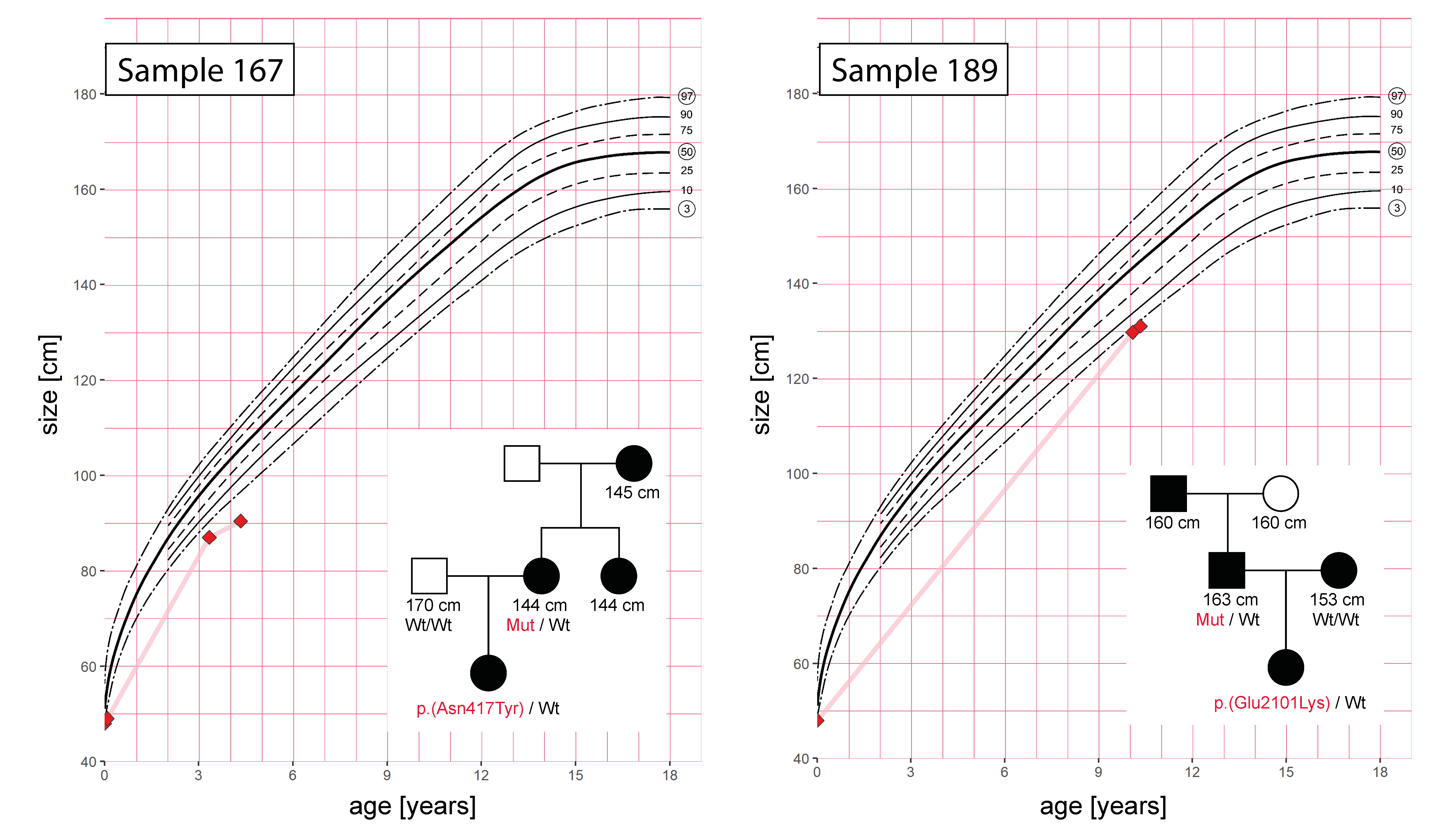


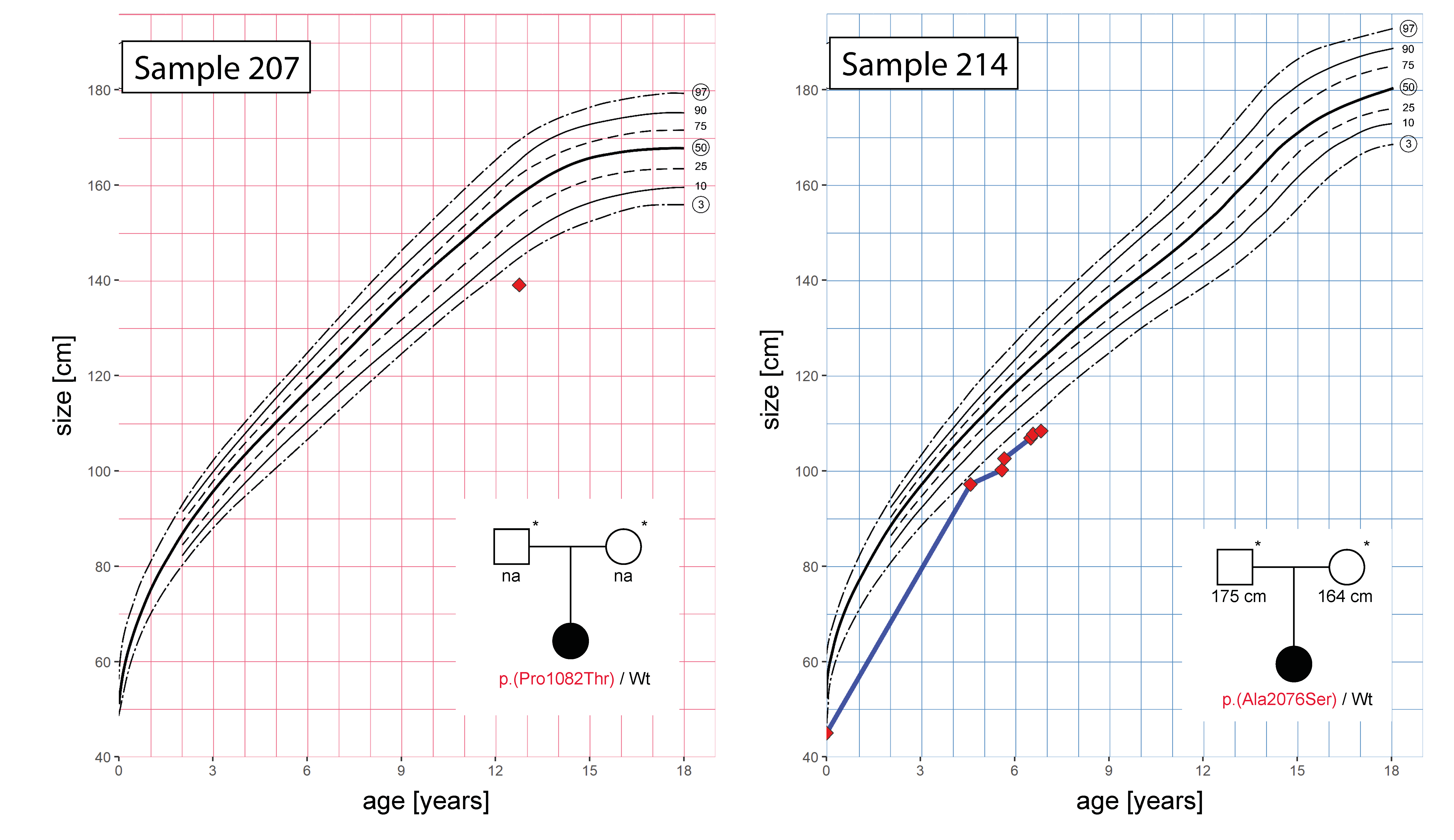


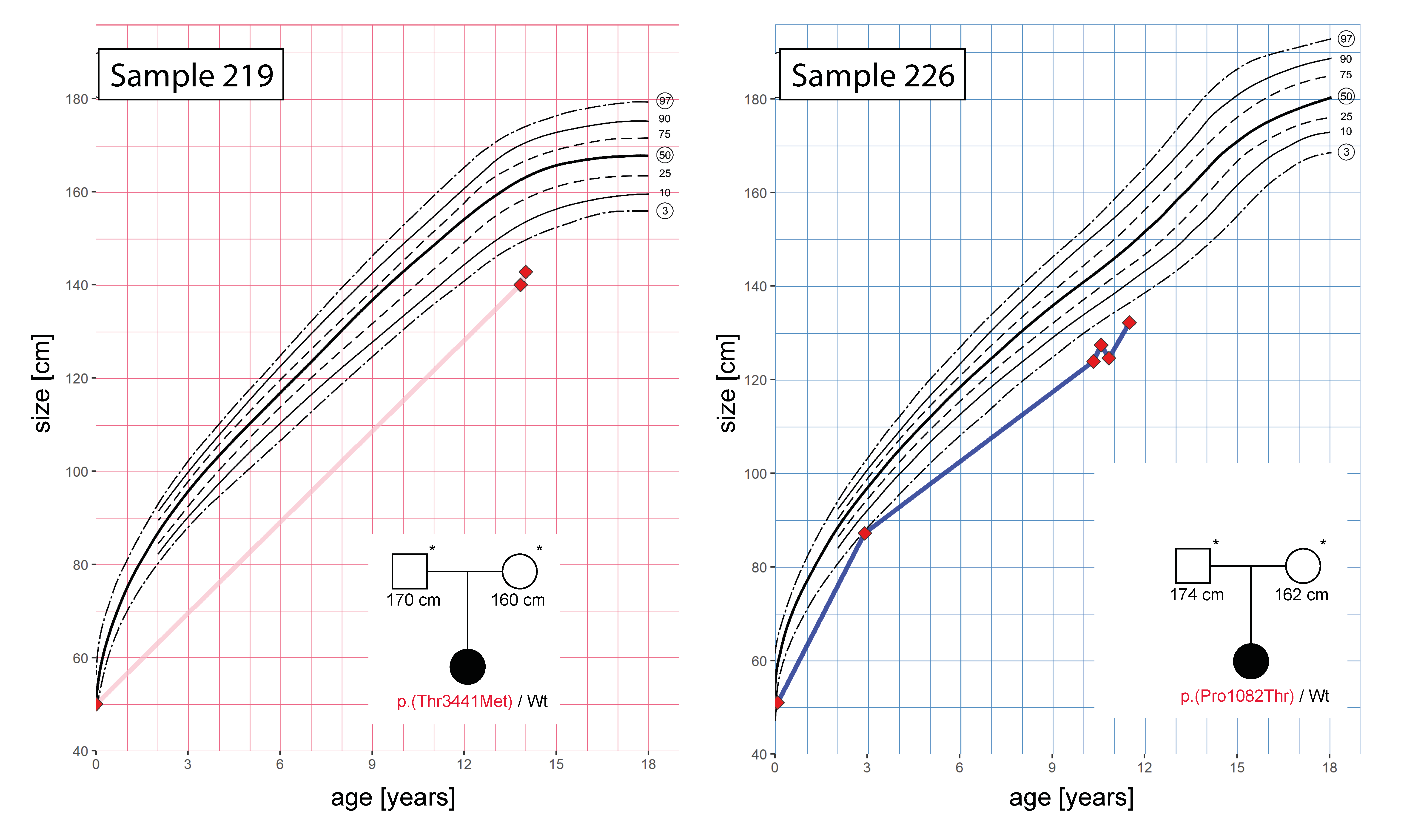


Supplementary Fig. 11. High-confidence candidate gene *LAMA5*. Growth charts and pedigrees of affected individuals. Standard growth curves were derived from Reinken et al.^70^ Embedded pedigrees depict the observed mode of inheritance (* parental DNA not available).


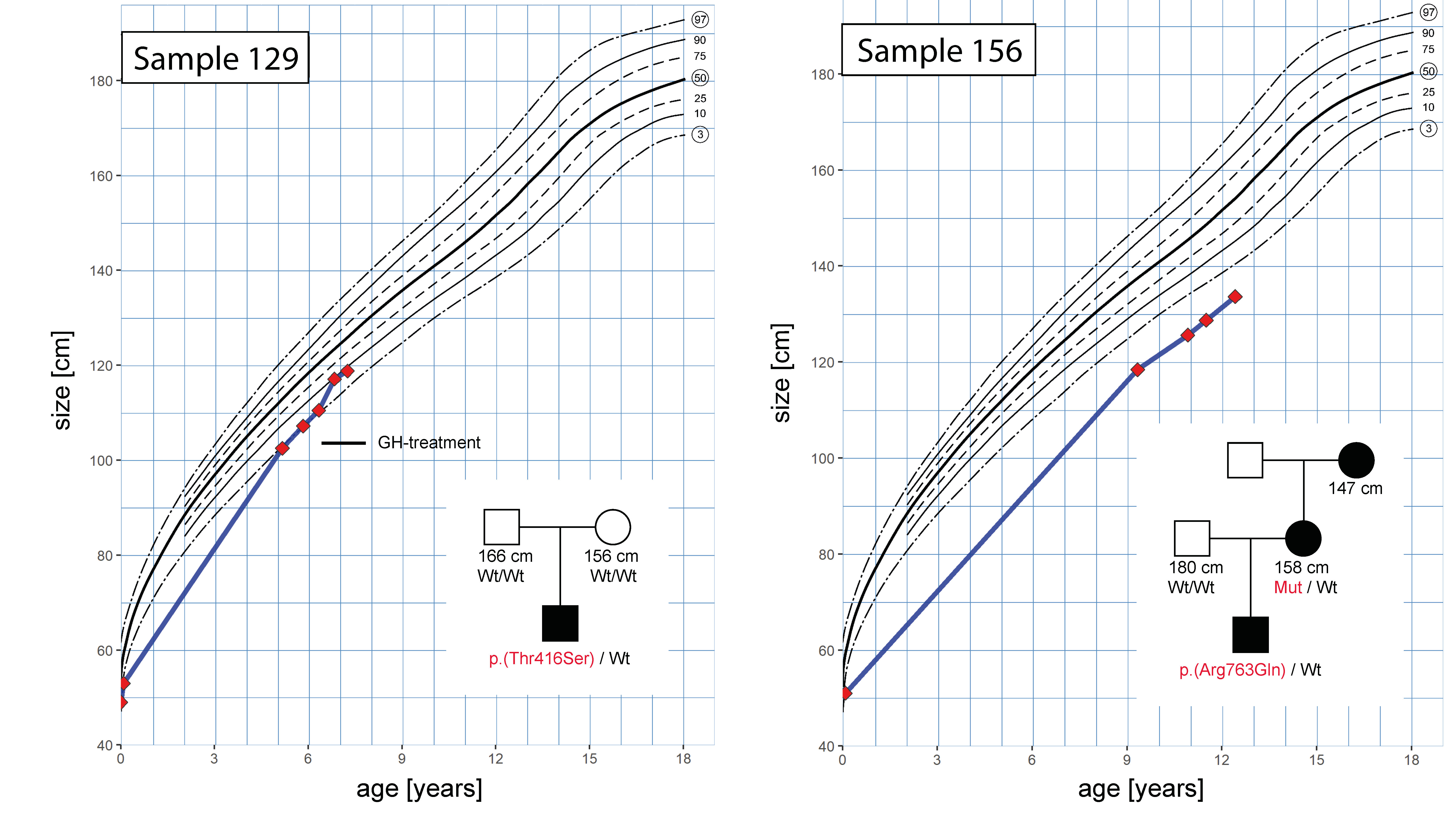


Supplementary Fig. 12. High-confidence candidate gene *MED24*. Growth charts and pedigrees of affected individuals. Standard growth curves were derived from Reinken et al.^70^ Embedded pedigrees depict the observed mode of inheritance. Growth hormone treatment in individual 129 resulted in increased growth velocity.


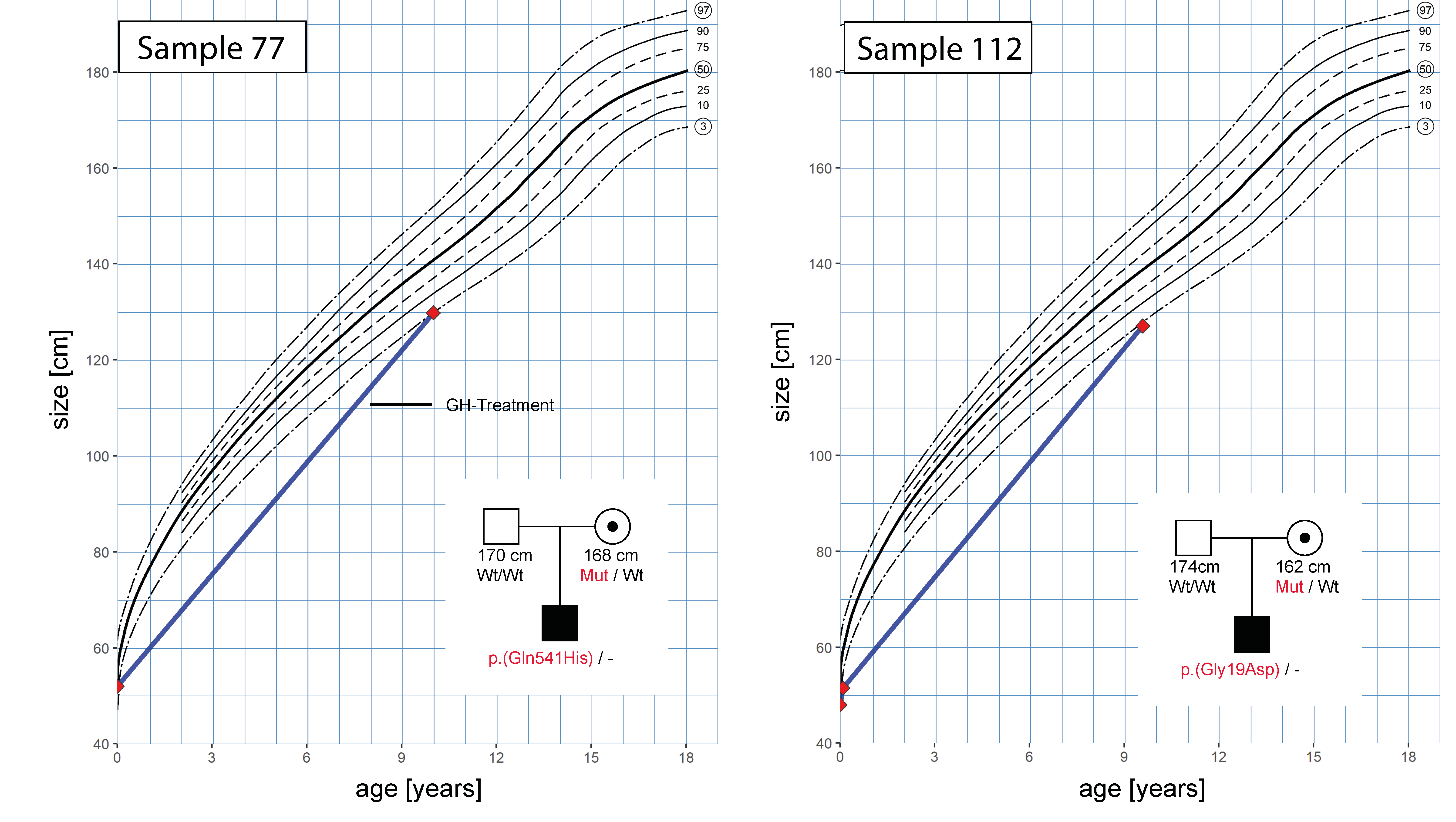


Supplementary Fig. 13. High-confidence candidate gene *PLXNA3*. Growth charts and pedigrees of affected individuals. Standard growth curves were derived from Reinken et al.^70^ Embedded pedigrees depict the observed mode of inheritance. Individual 77 received growth hormone treatment without increased growth velocity.


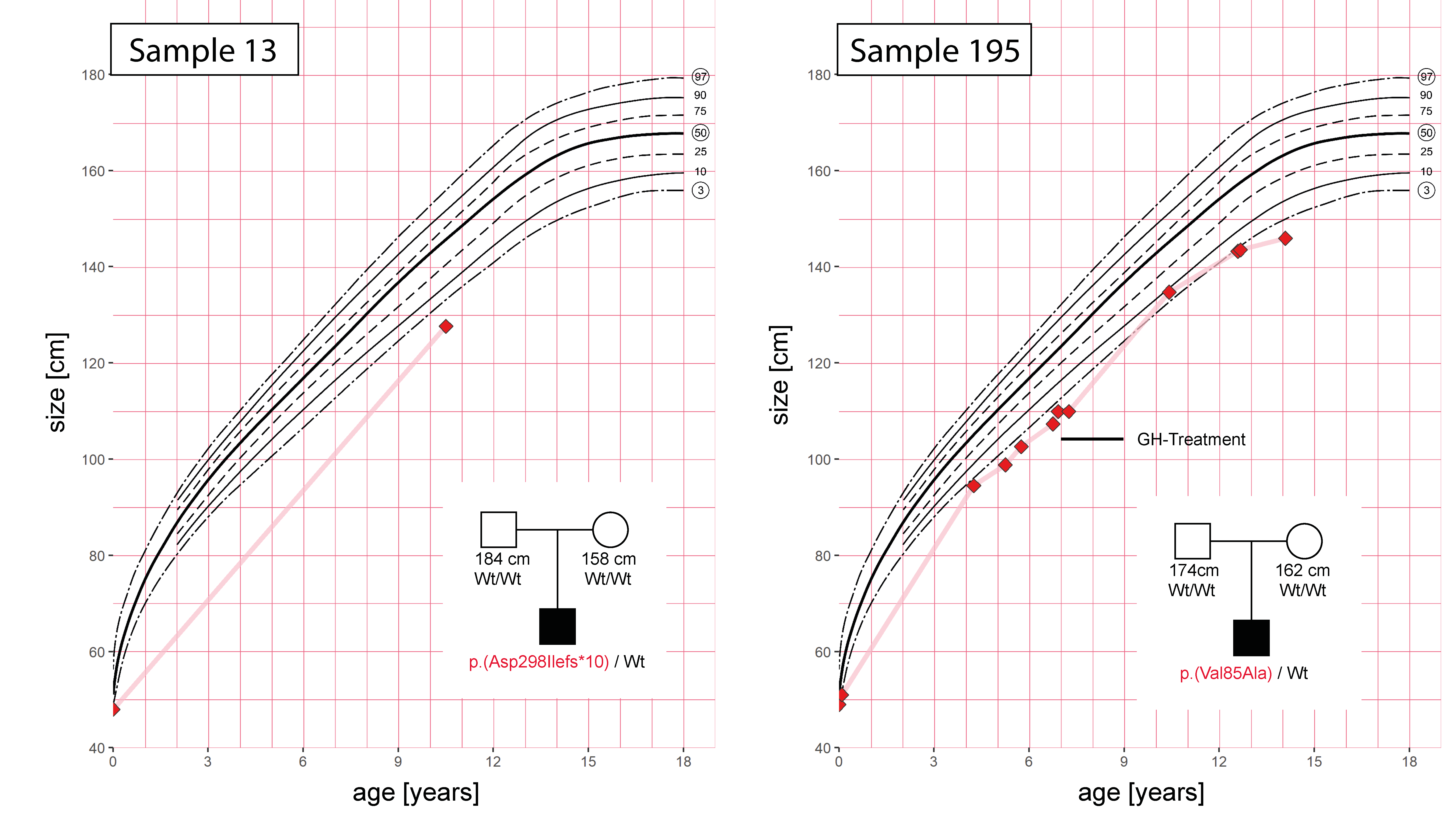


Supplementary Fig. 14. High-confidence candidate gene *RASA3*. Growth charts and pedigrees of affected individuals. Standard growth curves were derived from Reinken et al.^70^ Embedded pedigrees depict the observed mode of inheritance. Growth hormone treatment in individual 13 resulted in increased growth velocity.


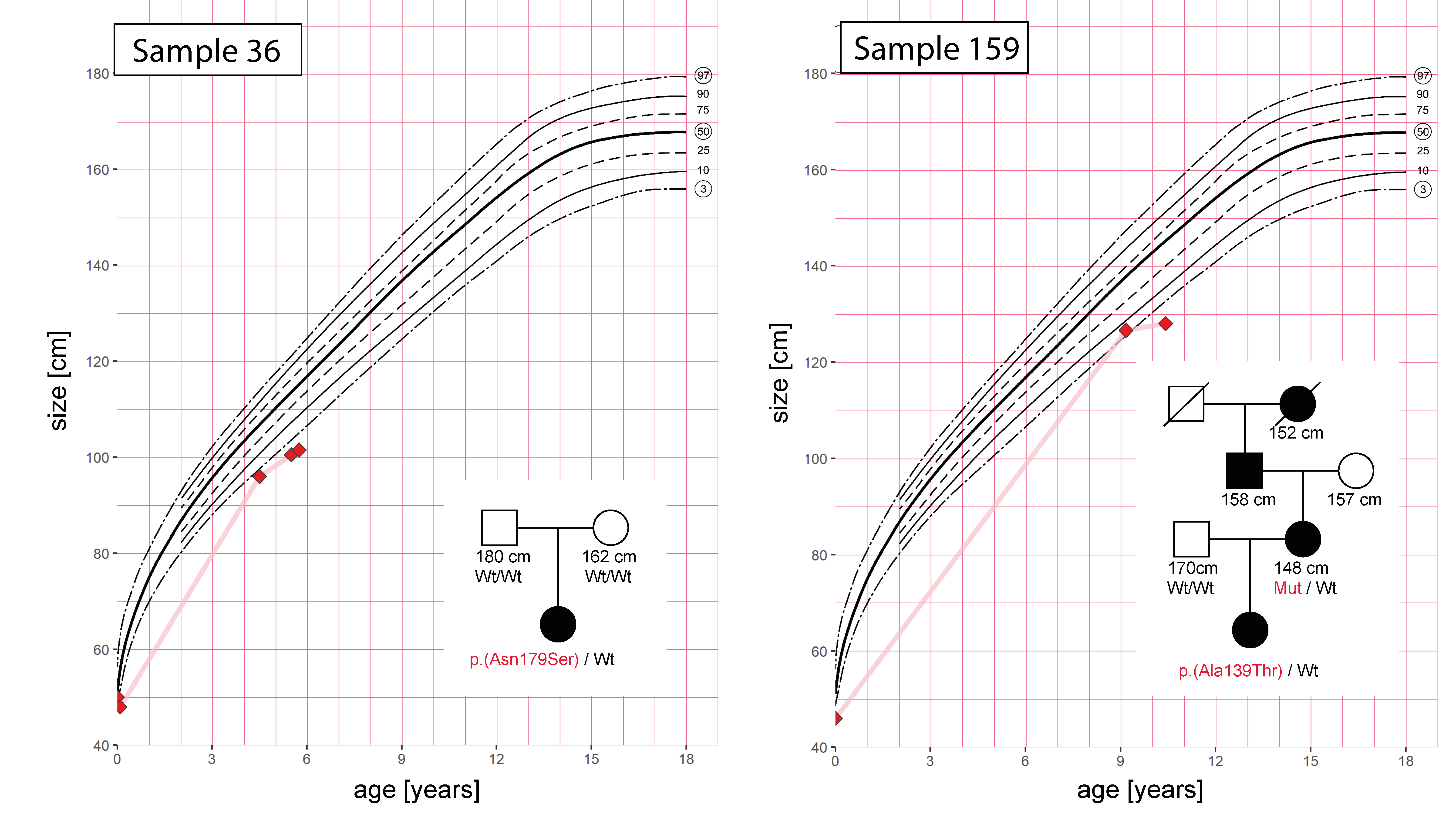


Supplementary Fig. 15. High-confidence candidate gene *SLC7A8*. Growth charts and pedigrees of affected individuals. Standard growth curves were derived from Reinken et al.^70^ Embedded pedigrees depict the observed mode of inheritance.


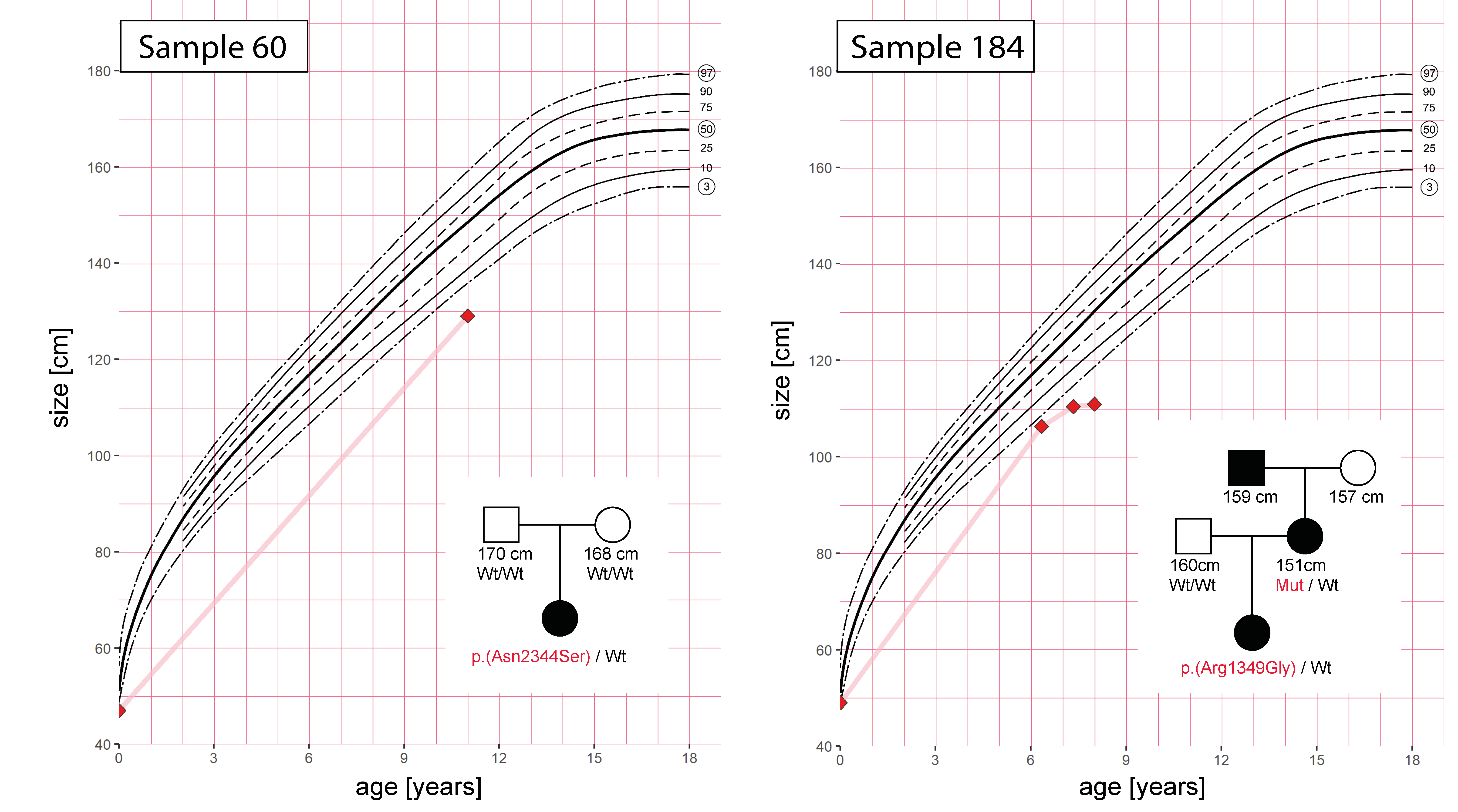


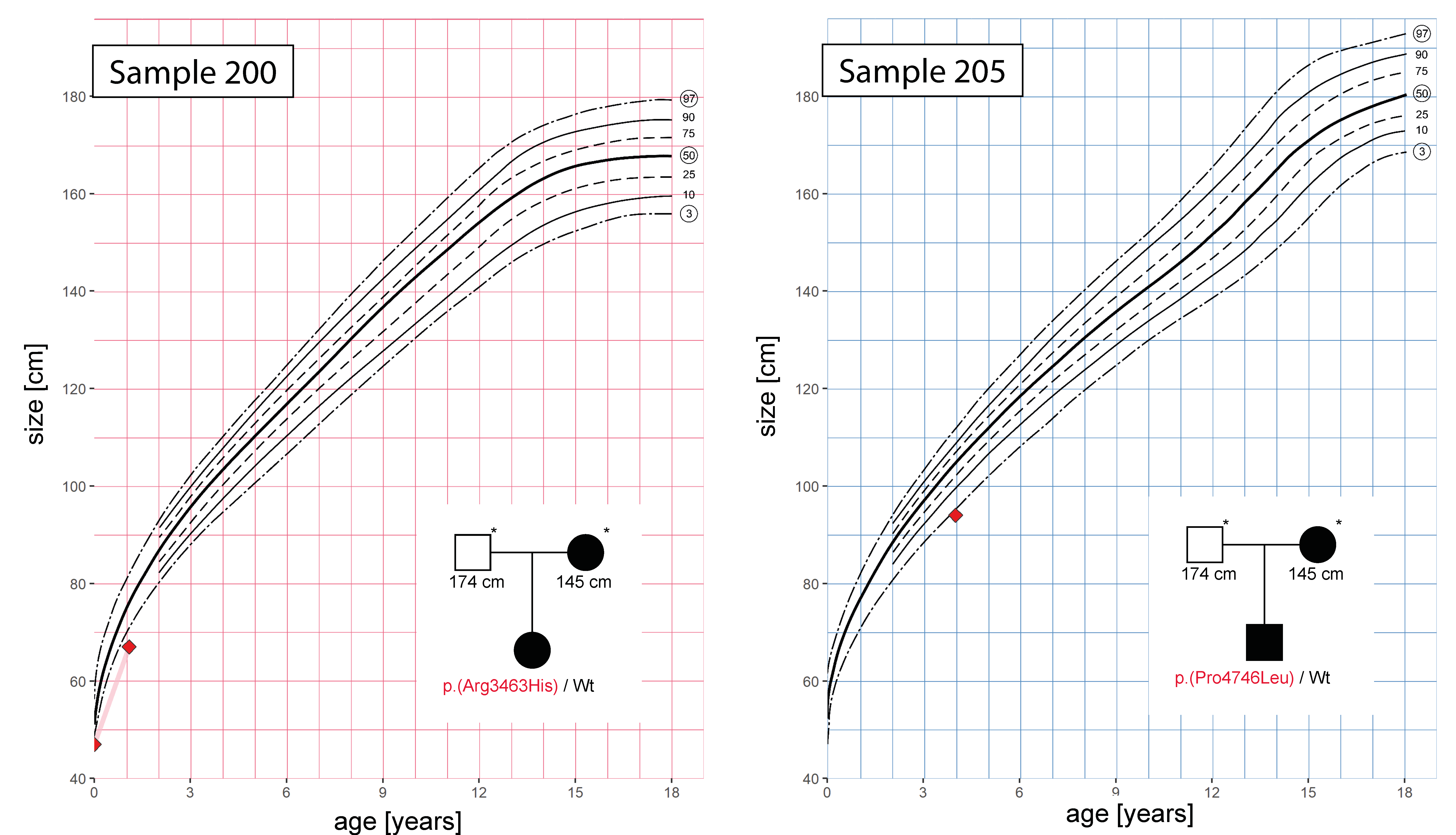


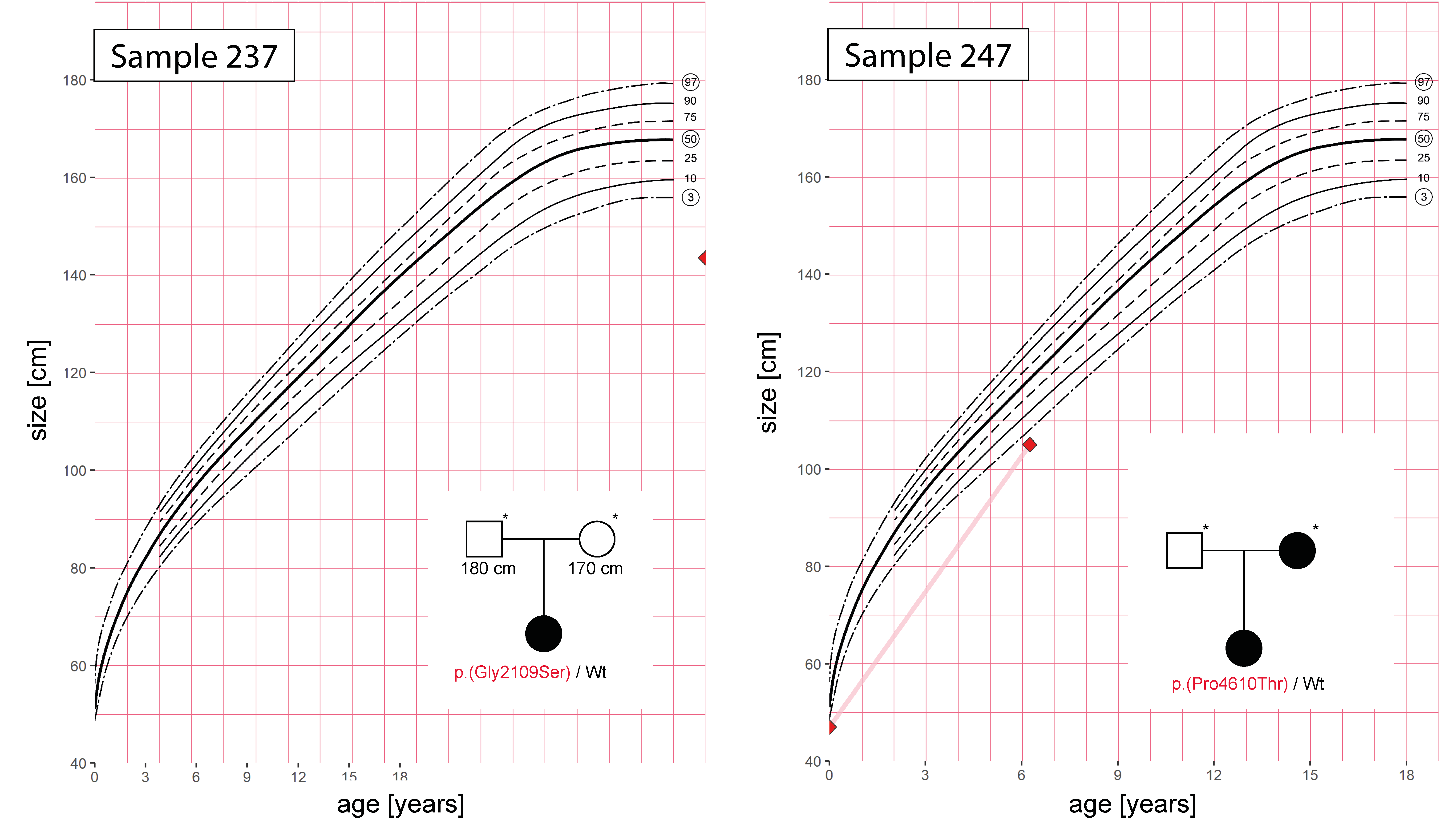


Supplementary Fig. 16. High-confidence candidate gene *UBR4*. Growth charts and pedigrees of affected individuals. Standard growth curves were derived from Reinken et al.^70^ Embedded pedigrees depict the observed mode of inheritance (* parental DNA not available).


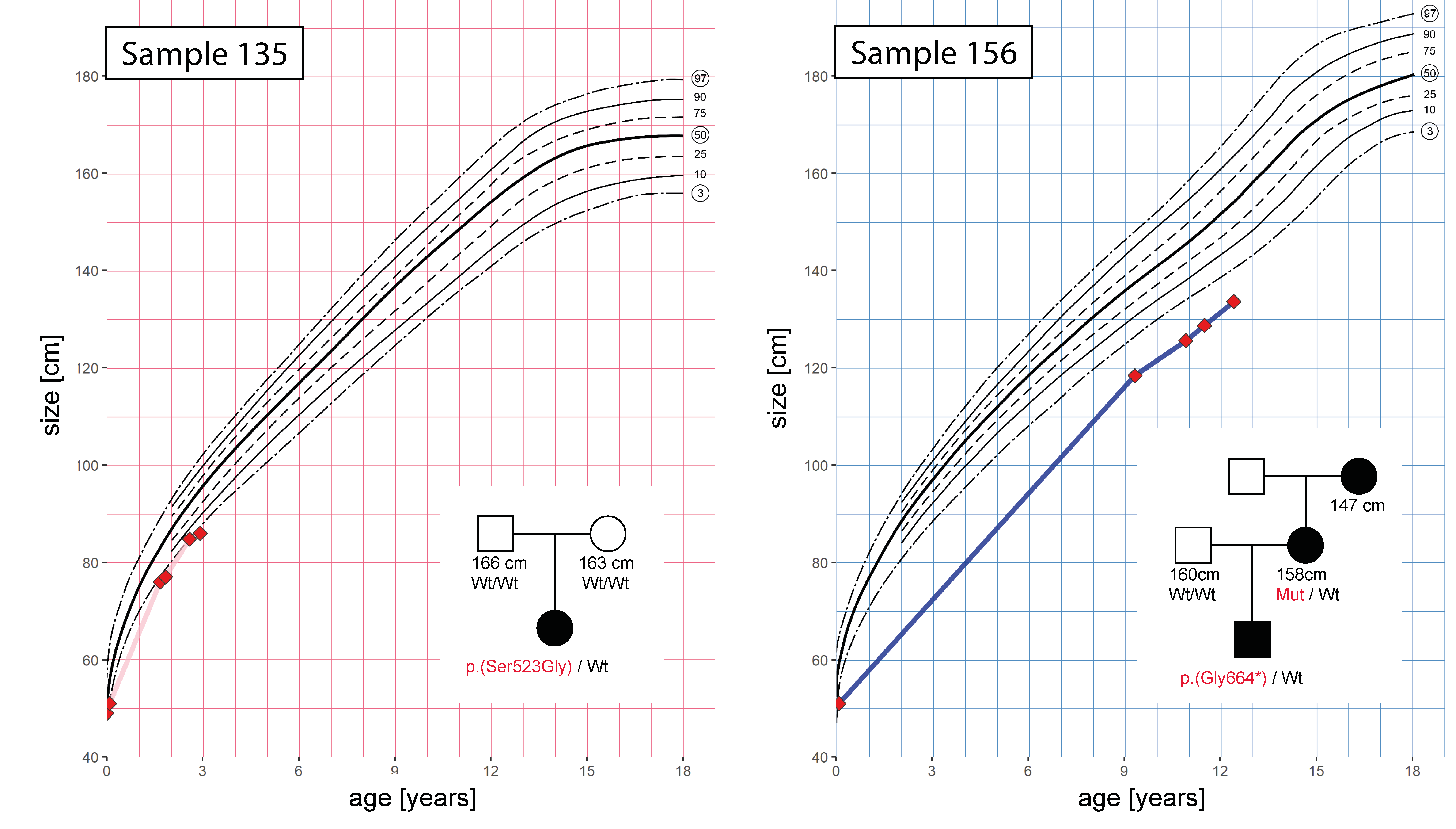


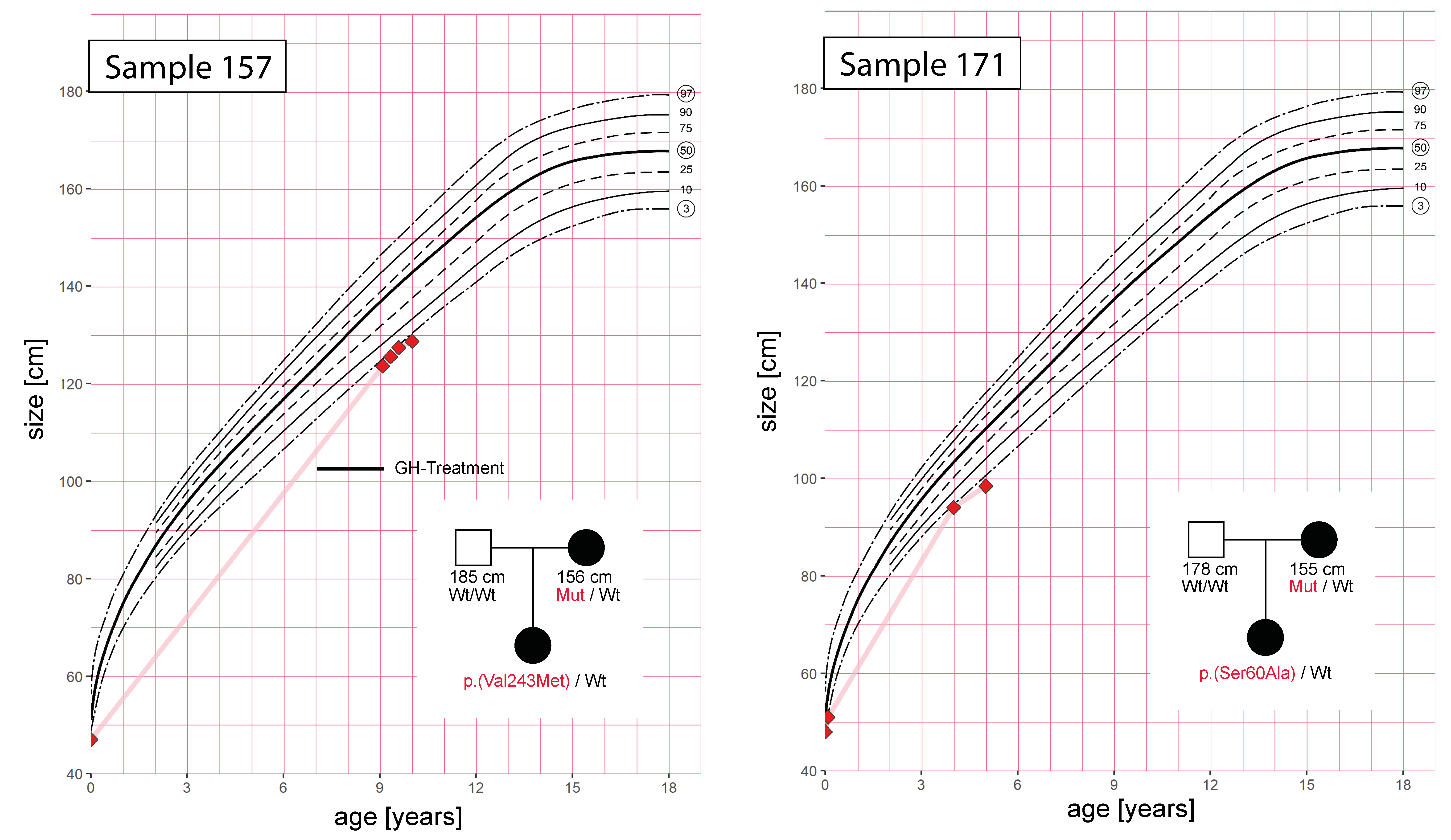
Supplementary Fig. 17. High-confidence candidate gene *USP45*. Growth charts and pedigrees of affected individuals. Standard growth curves were derived from Reinken et al.^70^ Embedded pedigrees depict the observed mode of inheritance. Growth hormone treatment in individual 158 resulted in increased growth velocity.


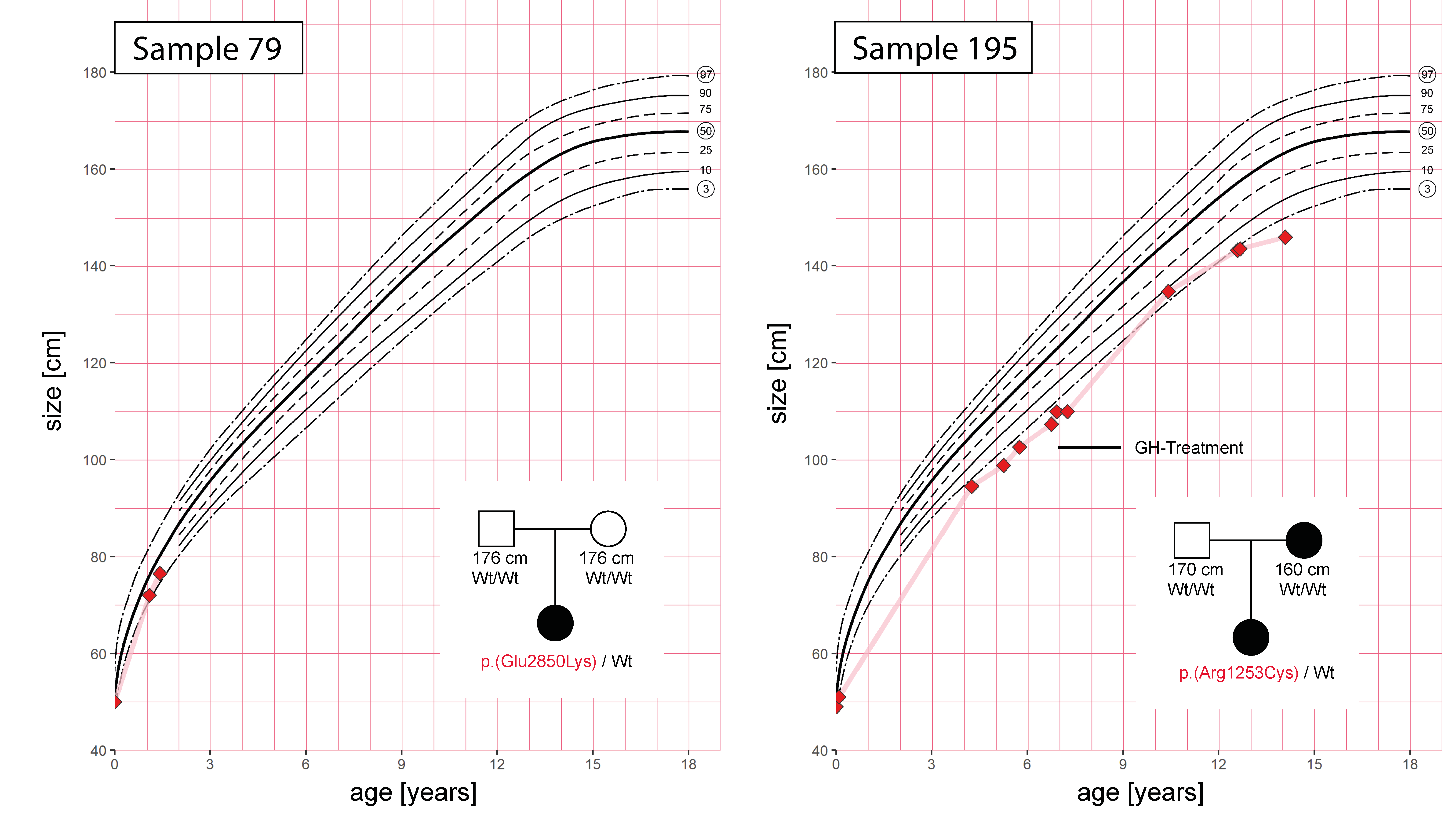


Supplementary Fig. 18. High-confidence candidate gene *ZFHX3*. Growth charts and pedigrees of affected individuals. Standard growth curves were derived from Reinken et al.^70^ Embedded pedigrees depict the observed mode of inheritance. Growth hormone treatment in individual 196 resulted in increased growth velocity.

Supplementary Tables

| Supplementary Table 1. Criteria for candidate gene variant scoring of dominant inherited variants | | | | |
| --- | --- | --- | --- | --- |
|  |  | ExAC frequency | | |
| Mutation type | CADD-score | **<10^-4^** | **10^-3^ –10^-4^** | **>10^-3^** |
| **missense** | **>15** | Likely Pathogenic | Unknown Significance | Benign |
|  | **10-15** | Unknown Significance | Likely Benign | Benign |
|  | **<10** | Likely Benign | Benign | Benign |
| **nonsense^1^** |  | Pathogenic | Pathogenic | Benign |
| **Inframe InDel (>1 AA)** |  | Likely Pathogenic | Unknown Significance | Benign |
| **Inframe InDel (1 AA)** |  | Unknown Significance | Likely Benign | Benign |
| **^1^ STOP-mutation, frameshift-InDel** | | | | |

| Supplementary Table 2. Criteria for candidate gene variant scoring of recessive inherited variants | | | |
| --- | --- | --- | --- |
|  |  | ExAC frequency | |
| Mutation type | CADD-score | **<10^-3^** | **>10^-3^** |
| **missense** | **>15** | Likely Pathogenic | Benign |
|  | **10-15** | Unknown Significance | Benign |
|  | **<10** | Likely Benign | Benign |
| **nonsense^1^** |  | Pathogenic | Benign |
| **Inframe InDel (>1 AA)** |  | Likely Pathogenic | Benign |
| **Inframe InDel (1 AA)** |  | Unknown Significance | Benign |
| **^1^ STOP-mutation, frameshift-InDel** | | | |

| Supplementary Table 3. Criteria for candidate gene variant scoring of splice site variants | | | | | |
| --- | --- | --- | --- | --- | --- |
|  |  | dbscSNV_ada_score^10^ | | dbscSNV_rf_score^10^ | |
| Position | Spidex^11^ | **<0.6** | **>0.6** | **<0.6** | **>0.6** |
| **-12 to -5 or -3 or +3 to +5** | **<-2.0 or >2.0** | Likely Pathogenic | | | |
|  | **na** | Unknown Significance | | | |
|  | **-2,0 to 2,0** | Likely Benign^1^ | Unknown Significance^2^ | Likely Benign^1^ | Unknown Significance^2^ |
| **-2,-1,+1,+2, or confirmed effect on splicing by rtPCR** |  | Pathogenic | | | |
| **InDel** |  | Unknown Significance | | | |

^1^ Variant is classified as Likely Benign if both, dbscSNV_ada_score and dbscSNV_rf_score are <0.6.

^2^ Variant is classified as variant of Unknown Significance if either dbscSNV_ada_score or dbscSNV_rf_score is >0.6.

| Supplementary Table 4. Gene level evaluation of candidate genes | | | | | | | | | | |
| --- | --- | --- | --- | --- | --- | --- | --- | --- | --- | --- |
| Gene | GWAS | Exome studies | CNVs  (No. in Decipher) | Mouse | Zebrafish | Gene Interaction | Chondrocyte expression | GO terms | ∑ gene level classes | |
| ***ABCF3*** | - | - | X(1) | - | - | - | X | X | 3 | |
| ***ABP1*** | - | - | X(4) | - | - | X | - | X | 3 | |
| ***ACSS3*** | - | - | - | - | - | X | - | X | 2 | |
| ***AHNAK*** | - | - | - | X | - | - | X | - | 2 | |
| ***AMMECR1*** | - | - | X(5) | - | - | - | - | - | 1 | |
| ***ANKS3*** | - | - | - | - | - | - | X | - | 1 | |
| ***ARHGAP29*** | - | - | X(2) | - | - | X | X | - | 3 | |
| ***ARHGEF40^+^*** | - | - | X(2) | - | - | - | X | - | 2 | |
| ***ARMCX6*** | - | - | X(5) | - | - | - | X | - | 2 | |
| ***ATP1B2*** | - | - | X(3) | - | - | - | X | X | 3 | |
| ***ATP2C2*** | - | - | X(1) | - | - | - | - | X | 2 | |
| ***ATP8B2*** | - | - | X(2) | - | - | - | X | X | 3 | |
| ***BAI3^#^*** | - | - | - | - | - | - | - | - | 0 | |
| ***BEX2*** | - | - | X(6) | - | - | - | X | - | 2 | |
| ***BRD4*** | - | - | X(2) | X | - | X | X | X | 5 | |
| ***CACNA2D2*** | - | - | X(1) | X | - | - | - | X | 3 | |
| ***CCDC120^#^*** | - | - | X(6) | - | - | - | X | - | 2 | |
| ***CCDC176^+^*** | - | - | X(1) | - | - | - | - | X | 2 | |
| ***CCDC73*** | - | - | - | - | - | - | - | - | 0 | |
| ***CCDC91*** | X | - | X(5) | - | - | - | X | X | 4 | |
| ***CCNB3*** | - | - | X(5) | - | - | X | - | - | 2 | |
| ***CD99L2*** | - | - | X(6) | - | - | - | X | - | 2 | |
| ***CDK5RAP3*** | - | - | - | - | - | - | X | X | 2 | |
| ***CEP76^#^*** | - | - | X(3) | - | - | X | - | - | 2 | |
| ***CERS4*** | - | - | - | - | - | X | X | X | 3 | |
| ***CHD1L*** | X | - | X(23) | - | - | X | X | X | 5 | |
| ***CLGN^+^*** | - | - | X(5) | - | - | - | - | - | 1 | |
| ***CLIC4*** | X | - | - | X | - | X | X | X | 5 | |
| ***CLIP1*** | - | - | X(1) | - | - | X | X | X | 4 | |
| ***CLTCL1*** | - | - | X(14) | - | - | - | - | - | 1 | |
| ***CNGA2*** | - | - | X(6) | X | - | - | - | X | 3 | |
| ***CNTROB*** | - | - | X(2) | - | - | - | X | - | 2 | |
| ***COL15A1*** | X | - | - | - | - | X | X | X | 4 | |
| ***CPSF1*** | - | - | - | - | - | - | X | - | 1 | |
| ***CPZ^#^*** | X | - | - | - | - | - | - | - | 1 | |
| ***CRNN*** | - | - | X(3) | - | - | - | - | X | 2 | |
| ***CSMD3*** | - | - | X(1) | - | - | X | - | - | 2 | |
| ***CUX2*** | - | - | - | - | - | X | - | X | 2 | |
| ***CXorf27*** | - | - | X(5) | - | - | - | - | - | 1 | |
| ***DAAM2*** | - | - | - | X | - | - | X | - | 2 | |
| ***DDX54*** | - | - | - | - | - | X | X | X | 3 | |
| ***DENND4B^#^*** | - | - | X(2) | - | - | - | X | - | 2 | |
| ***DHRS7*** | - | - | X(1) | - | - | - | X | - | 2 | |
| ***DIAPH2*** | - | - | X(7) | - | - | - | X | - | 2 | |
| ***DIO3*** | - | - | X(1) | X | - | X | - | X | 4 | |
| ***DNAH17^#^*** | - | - | X(1) | - | - | X | - | X | 3 | |
| ***DOCK11*** | - | - | X(5) | - | - | X | X | - | 3 | |
| ***DPF3*** | - | - | - | - | - | X | - | - | 1 | |
| ***DPRX*** | - | - | X(3) | - | - | - | - | X | 2 | |
| ***DRP2*** | - | - | X(5) | - | - | - | - | - | 1 | |
| ***EDEM3^#^*** | - | - | X(1) | - | - | - | X | X | 3 | |
| ***EXOC8*** | - | - | X(3) | - | - | - | X | - | 2 | |
| ***FAM124B*** | - | - | - | - | - | - | - | - | 0 | |
| ***FAM129B*** | - | - | - | - | - | X | X | - | 2 | |
| ***FAM135B^+^*** | - | - | - | - | - | - | - | - | 0 | |
| ***FAM160A2*** | - | - | - | - | - | - | X | - | 1 | |
| ***FAM3A*** | - | - | X(9) | - | - | - | X | - | 2 | |
| ***FBRS^#^*** | - | - | X(1) | - | - | - | X | X | 3 | |
| ***FBXO17*** | - | - | - | - | - | - | X | - | 1 | |
| ***FGF18*** | X | - | X(1) | X | X | X | - | X | 6 | |
| ***FZD2^#^*** | - | - | X(2) | - | X | X | - | X | 4 | |
| ***GABRE*** | - | - | X(6) | - | - | - | - | - | 1 | |
| ***GALNT1*** | - | - | X(1) | - | - | - | X | - | 2 | |
| ***GNG13*** | - | - | - | - | - | X | - | X | 2 | |
| ***GOLT1A*** | - | - | X(1) | - | - | - | X | - | 2 | |
| ***GOSR1*** | - | - | - | - | - | X | X | - | 2 | |
| ***GREB1L^+^*** | - | - | - | - | X | - | - | - | 1 | |
| ***GRIN3A*** | - | - | X(1) | - | - | - | - | X | 2 | |
| ***GXYLT2*** | - | - | X(1) | - | - | - | X | - | 2 | |
| ***HERC2*** | - | X | X(6) | - | - | - | X | X | 4 | |
| ***HIVEP1*** | - | - | X | - | - | X | X | X | 4 | |
| ***HKDC1^+^*** | - | - | - | - | - | X | - | X | 2 | |
| ***HNRNPA0*** | - | - | X(1) | - | - | X | X | X | 4 | |
| ***HOOK3^+^*** | - | - | X(1) | - | - | - | X | X | 3 | |
| ***HSP90AB1*** | - | X | - | - | - | X | X | X | 4 | |
| ***HSPA6*** | - | - | X(2) | - | - | X | - | X | 3 | |
| ***IBTK*** | - | - | - | - | - | X | X | X | 3 | |
| ***IFT81^#^*** | - | - | - | - | - | X | - | X | 2 | |
| ***IGDCC4*** | - | - | X(1) | - | - | - | X | - | 2 | |
| ***IGSF1*** | - | - | X(5) | - | - | X | - | - | 2 | |
| ***IQCA1*** | - | - | X(1) | - | - | - | X | X | 3 | |
| ***IQUB*** | - | - | X(5) | - | - | - | - | X | 2 | |
| ***ITIH6^#^*** | - | - | X(5) | - | - | - | X | - | 2 | |
| ***ITM2A*** | - | - | X(5) | - | - | - | X | - | 2 | |
| ***JAM2*** | - | - | - | - | - | - | X | X | 2 | |
| ***KATNAL2*** | - | - | - | - | - | - | - | X | 1 | |
| ***KCND1^#^*** | - | - | X(6) | - | - | - | - | - | 1 | |
| ***KIAA1524*** | - | - | - | - | - | - | - | - | 0 | |
| ***KIF18A^+^*** | - | - | X(1) | - | - | X | - | X | 3 | |
| ***KLHDC3*** | - | - | - | - | - | - | X | X | 2 | |
| ***LAMA5^#^*** | - | - | X(1) | X | - | X | - | X | 4 | |
| ***LDLRAD4*** | - | - | X(3) | - | - | - | - | - | 1 | |
| ***LRIG3*** | X | - | X(2) | - | - | X | X | - | 4 | |
| ***LTBP1*** | X | - | X(1) | X | X | X | X | X | 7 | |
| ***LZTR1^+^*** | - | - | X(18) | - | - | X | X | - | 3 | |
| ***MACF1^#^*** | - | - | - | X | - | - | X | X | 3 | |
| ***MAD1L1*** | - | - | X(2) | - | - | X | X | X | 4 | |
| ***MAGEB2*** | - | - | X(4) | - | - | X | - | - | 2 | |
| ***MB21D2*** | - | - | - | - | - | - | - | - | 0 | |
| ***MDGA2^+^*** | - | - | X(1) | - | - | - | - | X | 2 | |
| ***MED24^#^*** | - | - | X(1) | X | - | X | X | X | 5 | |
| ***MFSD7*** | - | - | X(10) | - | - | - | X | - | 2 | |
| ***MPRIP*** | - | - | X(10) | - | - | - | X | - | 2 | |
| ***MTA3*** | - | - | X(1) | - | X | X | X | X | 5 | |
| ***MTMR1*** | - | - | X(6) | - | - | - | X | - | 2 | |
| ***MVP*** | - | - | X(17) | - | - | X | X | X | 4 | |
| ***MXRA5^#^*** | - | - | X(12) | - | - | X | X | - | 3 | |
| ***MYCBPAP*** | - | - | - | - | - | - | - | - | 0 | |
| ***MYH7B^#^*** | - | - | X(1) | - | - | X | - | X | 3 | |
| ***MYL6*** | - | - | - | - | - | X | X | X | 3 | |
| ***MYO3B*** | - | - | X(4) | - | - | - | - | X | 2 | |
| ***N4BP2L2*** | X | - | X(2) | - | - | - | X | X | 4 | |
| ***NADSYN1*** | - | - | - | - | - | X | X | X | 3 | |
| ***NAP1L2*** | - | - | X(5) | - | - | - | - | X | 2 | |
| ***NAT10*** | - | - | - | - | - | X | X | X | 3 | |
| ***NCAPG*** | X | - | - | - | - | X | X | X | 4 | |
| ***NCOR2*** | - | - | X(3) | X | - | X | X | X | 5 | |
| ***NFXL1*** | - | - | - | X | - | - | X | - | 2 | |
| ***NISCH*** | - | - | X(2) | - | - | - | X | X | 3 | |
| ***NKX6-1*** | - | - | X(3) | - | - | X | - | X | 3 | |
| ***NLRP5*** | - | - | X(2) | - | - | - | - | X | 2 | |
| ***NRP2*** | - | - | X(1) | X | X | X | X | X | 6 | |
| ***NUP214*** | - | - | X(2) | - | - | X | X | X | 4 | |
| ***OBSCN^#^*** | - | - | X(3) | - | - | X | - | X | 3 | |
| ***OSBP*** | - | - | X(1) | - | - | - | X | - | 2 | |
| ***PABPN1L*** | - | - | X(5) | - | - | - | - | X | 2 | |
| ***PAICS*** | - | - | - | - | - | X | X | X | 3 | |
| ***PANK4*** | - | - | X(10) | - | - | X | X | X | 4 | |
| ***PDCD11*** | - | - | X(1) | - | - | X | X | - | 3 | |
| ***PDDC1*** | - | - | X(1) | - | - | - | X | - | 2 | |
| ***PDZRN3^+^*** | - | - | X(1) | - | - | - | X | - | 2 | |
| ***PGM5*** | - | - | X(4) | - | - | X | - | - | 2 | |
| ***PHF20^+^*** | X | - | X(2) | - | - | X | X | - | 4 | |
| ***PIK3C2A*** | - | - | - | X | - | X | X | X | 4 | |
| ***PLXNA3^#^*** | - | - | X(9) | - | X | X | X | X | 5 | |
| ***PLXNB3*** | - | - | X(7) | - | - | - | X | X | 3 | |
| ***POLR2E*** | - | - | - | - | - | X | X | X | 3 | |
| ***PPDPF*** | - | - | X(3) | - | - | - | X | - | 2 | |
| ***PSMD11*** | - | - | X(2) | - | - | X | X | X | 4 | |
| ***PTH2R*** | - | - | - | - | - | X | - | - | 1 | |
| ***PTK2B*** | - | - | X(2) | - | X | X | - | X | 4 | |
| ***PTPLAD1*** | - | - | X(2) | - | - | - | X | X | 3 | |
| ***PTPRD*** | - | - | X(6) | - | - | X | X | X | 4 | |
| ***PTPRU^+^*** | - | - | X(2) | - | X | X | X | X | 5 | |
| ***PXN*** | - | - | - | X | - | X | X | X | 4 | |
| ***PYGB*** | - | X | X(1) | - | - | X | X | X | 5 | |
| ***RAB41*** | - | - | X(4) | - | - | X | - | X | 3 | |
| ***RAI2*** | - | - | X(5) | - | - | - | X | - | 2 | |
| ***RASA3^#^*** | - | - | X(3) | X | - | X | X | - | 4 | |
| ***RBM3*** | - | - | X(5) | - | - | - | X | X | 3 | |
| ***RGAG4*** | - | - | X(5) | - | - | - | X | - | 2 | |
| ***RHOF*** | - | - | - | - | - | X | X | X | 3 | |
| ***RIMBP2*** | - | - | X(3) | - | - | - | - | - | 1 | |
| ***RNF144A*** | - | - | X(2) | - | - | - | X | - | 2 | |
| ***RNF31^+^*** | - | - | X(1) | - | - | X | X | X | 4 | |
| ***ROS1^+^*** | - | - | X(2) | - | - | - | - | X | 2 | |
| ***RUNX1T1*** | - | - | - | X | - | X | X | X | 4 | |
| ***SEPT7*** | - | - | X(1) | - | - | X | X | X | 4 | |
| ***SFI1^+^*** | - | - | - | X | - | X | X | - | 3 | |
| ***SHROOM4*** | - | - | X(5) | - | - | - | - | X | 2 | |
| ***SIPA1L2*** | - | - | X(3) | - | - | - | X | - | 2 | |
| ***SLC16A14*** | - | - | X(1) | - | - | - | - | - | 1 | |
| ***SLC25A23*** | - | - | X(1) | X | - | - | X | - | 3 | |
| ***SLC38A3^#^*** | - | - | X(1) | - | - | - | X | - | 2 | |
| ***SLC47A1*** | - | - | X(9) | - | - | - | - | - | 1 | |
| ***SLC7A8^#^*** | - | - | X(1) | - | - | - | X | X | 3 | |
| ***SMARCA5*** | X | - | X(2) | X | - | X | X | X | 6 | |
| ***SMOX*** | X | - | - | X | X | X | X | - | 5 | |
| ***SPEF2*** | - | - | X(1) | - | - | - | - | X | 2 | |
| ***SRCRB4D*** | - | - | - | - | - | - | - | - | 0 | |
| ***SRPX^#^*** | - | - | X(5) | - | - | - | X | X | 3 | |
| ***SSC5D*** | - | - | X(3) | - | - | - | X | - | 2 | |
| ***STAP1*** | - | - | X(3) | - | - | X | - | X | 3 | |
| ***STARD8*** | - | - | X(4) | - | - | X | - | - | 2 | |
| ***SVEP1*** | - | - | X(1) | - | - | - | X | X | 3 | |
| ***TBC1D25*** | - | - | X(5) | - | - | - | X | - | 2 | |
| ***THADA*** | X | - | X(1) | - | - | X | X | - | 4 | |
| ***TKTL1*** | - | - | X(8) | - | - | X | - | - | 2 | |
| ***TMEM59*** | - | - | - | - | - | - | X | - | 1 | |
| ***TNK2*** | - | - | X(1) | - | - | X | X | X | 4 | |
| ***TNRC6A*** | - | - | X(2) | - | - | X | X | X | 4 | |
| ***TOX3*** | - | - | - | - | - | X | - | X | 2 | |
| ***TRA2A*** | - | - | X(2) | - | - | - | X | X | 3 | |
| ***TTBK1*** | - | - | - | - | - | - | - | X | 1 | |
| ***TULP3*** | - | - | - | - | - | - | X | X | 2 | |
| ***TXNDC2*** | - | - | X(3) | - | - | - | - | X | 2 | |
| ***UBL4A*** | - | - | X(8) | - | - | X | X | - | 3 | |
| ***UBR4^#^*** | - | - | X(2) | X | - | X | X | - | 4 | |
| ***USP24^#^*** | - | - | - | X | - | - | X | - | 2 | |
| ***USP45^#^*** | - | - | X(1) | - | - | X | - | - | 2 | |
| ***USP51*** | - | - | X(5) | - | - | - | - | - | 1 | |
| ***VWCE*** | - | - | - | - | - | X | - | - | 1 | |
| ***WDR6*** | - | X | X(1) | - | - | X | X | X | 5 | |
| ***WIPF2*** | - | - | X(1) | - | - | X | X | - | 3 | |
| ***WNT9B*** | - | X | - | - | - | X | - | X | 3 | |
| ***WTAP*** | - | - | - | - | - | X | X | - | 2 | |
| ***ZBED4*** | - | X | X(1) | - | - | - | X | - | 3 | |
| ***ZBTB34*** | - | - | - | - | - | - | - | - | 0 | |
| ***ZFC3H1^+^*** | - | - | - | - | - | - | X | - | 1 | |
| ***ZFHX3^#^*** | - | - | X(1) | X | - | X | X | X | 5 | |
| ***ZMYM3*** | - | - | X(5) | - | - | - | X | X | 3 | |
| ***ZNF284*** | - | - | X(1) | - | - | - | - | - | 1 | |
| ***ZNF446^#^*** | - | - | X(2) | - | - | - | X | X | 3 | |
| ***ZNF449^#^*** | - | - | X(6) | - | - | - | X | - | 2 | |
| ***ZNF502*** | - | - | X(1) | - | - | - | - | - | 1 | |
| ***ZNF691*** | - | - | X(1) | - | - | - | X | - | 2 | |
| ***ZSCAN18*** | - | - | X(2) | - | - | - | X | X | 3 | |
| ^#^: Candidate gene with variants in at least 2 affected individuals with validated segregation, ^+^: Candidate gene with variants in at least 2 affected individuals, whereby segregation could be validated in only one of them | | | | | | | | | |  |

| Supplementary Table 5. Candidate gene combined score calculation | | | | | | | | | |
| --- | --- | --- | --- | --- | --- | --- | --- | --- | --- |
| **Combined score** | **Applying gene categories (No.)** | | | | | | | | |
| **Variant Score** | **0** | **1** | **2** | **3** | **4** | **5** | **6** | **7** | **8** |
| **Pathogenic** | **3** | **4** | | | | **5** | | | |
| **Likely Pathogenic** | **2** | **3** | | | | **4** | | | |
| **Unknown Significance** | **1** | | **2** | | | **3** | | | |
| **Likely Benign** | **0** | | | | | | | | |
| **Benign** | **0** | | | | | | | | |

| Supplementary Table 6. Candidate gene ranking approach | | | | | | |
| --- | --- | --- | --- | --- | --- | --- |
|  | | | | | | |
| **No. of affected individuals**  **(for each candidate gene)*** | **≥ 2^1^** | | **≥ 2^2^** | | **1** | |
| **Combined score** | **≥ 3** | **< 3** | **≥ 3** | **< 3** | **≥ 4** | **< 4** |
| **High-confidence candidate gene** | X |  |  |  |  |  |
| **Medium-confidence candidate gene** |  | X | X |  | X |  |
| **Likely candidate gene** |  |  |  | X |  | X |
| When a candidate gene is present in ≥ 2 individuals the remaining variants where ranked accordingly. ^1^: segregation of variants in the same gene shown in at least two affected individuals; ^2^segregation of variants in the same gene shown in one affected individual and without proven segregation in at least one further affected individual | | | | | | |

| Supplementary Table 7. High-confidence candidate genes | | | | | | | | |
| --- | --- | --- | --- | --- | --- | --- | --- | --- |
| Gene | Sample ID | Genomic position (GRCh37) | RefSeq number | cDNA level | Protein level | Variant Level Classification | Gene Level Classification | Combined Score |
| ***autosomal dominant*** | | | | | | | | |
| ***CPZ*** | 57 | chr4:g.8603221C>T | NM_003652.3 | c.460C>T | p.(Arg154Trp) | Likely Pathogenic | 1 | **3** |
|  | 154 | chr4:g.8603221C>T | NM_003652.3 | c.460C>T | p.(Arg154Trp) | Likely Pathogenic | 1 | **3** |
|  | 230* | chr4:g.8613847G>T | NM_003652.3 | c.1288G>T | p.(Gly430Trp) | Likely Pathogenic | 1 | **3** |
| ***EDEM3*** | 165 | chr1:g.184686715A>C | NM_025191.3 | c.1204T>G | p.(Leu402Val) | Likely Pathogenic | 3 | **3** |
|  | 192 | chr1:g.184663565C>A | NM_025191.3 | c.2431G>T | p.(Asp811Tyr) | Likely Pathogenic | 3 | **3** |
| ***FBRS*** | 125 | chr16:g.30679943G>A | NM_001105079.1 | c.577G>A | p.(Gly193Ser) | Likely Pathogenic | 3 | **3** |
|  | 152 | chr16:g.30680919C>G | NM_001105079.1 | c.1336C>G | p.(Pro446Ala) | Likely Pathogenic | 3 | **3** |
|  | 208* | chr16:g.30680617C>T | NM_001105079.1 | c.1034C>T | p.(Pro345Leu) | Unknown Significance | 3 | **2** |
| ***IFT81*** | 166 | chr12:g.110566765C>T | NM_014055.3 | c.259C>T | p.(Arg87Cys) | Likely Pathogenic | 2 | **3** |
|  | 188 | chr12:g.110600832C>T | NM_014055.3 | c.1150C>T | p.(Arg384Cys) | Likely Pathogenic | 2 | **3** |
| ***LAMA5*** | 44 | chr20:g.60885734C>T | NM_005560.4 | c.10433G>A | p.(Ser3478Asn) | Unknown Significance | 4 | **2** |
|  | 162 | chr20:g.60897778T>C | NM_005560.4 | c.6101A>G | p.(Asp2034Gly) | Likely Pathogenic | 4 | **3** |
|  | 167 | chr20:g.60921595T>A | NM_005560.4 | c.1249A>T | p.(Asn417Tyr) | Unknown Significance | 4 | **2** |
|  | 189 | chr20:g.60897370C>T | NM_005560.4 | c.6301G>A | p.(Glu2101Lys) | Likely Pathogenic | 4 | **3** |
|  | 207* | chr20:g.60908184G>T | NM_005560.4 | c.3244C>A | p.(Pro1082Thr) | Likely Pathogenic | 4 | **3** |
|  | 214* | chr20:g.60897445C>A | NM_005560.4 | c.6226G>T | p.(Ala2076Ser) | Unknown Significance | 4 | **2** |
|  | 219* | chr20:g.60885845G>A | NM_005560.4 | c.10322C>T | p.(Thr3441Met) | Likely Pathogenic° | 4 | **3°** |
|  | 226* | chr20:g.60908184G>T | NM_005560.4 | c.3244C>A | p.(Pro1082Thr) | Likely Pathogenic | 4 | **3** |
| ***MED24*** | 129 | chr17:g.38186020G>C | NM_014815.3 | c.1247C>G | p.(Thr416Ser) | Likely Pathogenic | 5 | **4** |
|  | 156 | chr17:g.38179042C>T | NM_014815.3 | c.2288G>A | p.(Arg763Gln) | Likely Pathogenic° | 5 | **4°** |
| ***RASA3*** | 13 | chr13:g.114784286_114784289del | NM_007368.2 | c.892_895del | p.(Asp298Ilefs*10) | Pathogenic | 4 | **4** |
|  | 195 | chr13:g.114817550A>G | NM_007368.2 | c.254T>C | p.(Val85Ala) | Likely Pathogenic | 4 | **3** |
| ***SLC7A8*** | 36 | chr14:g.23608694T>C | NM_001267036.1 | c.536A>G | p.(Asn179Ser) | Likely Pathogenic | 3 | **3** |
|  | 159 | chr14:g.23609738C>T | NM_001267036.1 | c.415G>A | p.(Ala139Thr) | Likely Pathogenic | 3 | **3** |
| ***UBR4*** | 60 | chr1:g.19478319T>C | NM_020765.2 | c.7031A>G | p.(Asn2344Ser) | Likely Pathogenic | 4 | **3** |
|  | 184 | chr1:g.19493580G>C | NM_020765.2 | c.4045C>G | p.(Arg1349Gly) | Likely Pathogenic | 4 | **3** |
|  | 200* | chr1:g.19446118C>T | NM_020765.2 | c.10388G>A | p.(Arg3463His) | Likely Pathogenic | 4 | **3** |
|  | 205* | chr1:g.19415446G>A | NM_020765.2 | c.14237C>T | p.(Pro4746Leu) | Unknown Significance | 4 | **2** |
|  | 237* | chr1:g.19481545C>T | NM_020765.2 | c.6325G>A | p.(Gly2109Ser) | Likely Pathogenic | 4 | **3** |
|  | 247* | chr1:g.19420552G>T | NM_020765.2 | c.13828C>A | p.(Pro4610Thr) | Likely Pathogenic | 4 | **3** |
| ***USP45*** | 135 | chr6:g.99894081T>C | NM_001080481.1 | c.1567A>G | p.(Ser523Gly) | Likely Pathogenic° | 2 | **3°** |
|  | 156 | chr6:g.99891524C>A | NM_001080481.1 | c.1990G>T | p.(Gly664*) | Pathogenic | 2 | **4** |
|  | 157 | chr6:g.99930747C>T | NM_001080481.1 | c.727G>A | p.(Val243Met) | Likely Pathogenic | 2 | **3** |
|  | 171 | chr6:g.99956581A>C | NM_001080481.1 | c.178T>G | p.(Ser60Ala) | Likely Pathogenic | 2 | **3** |
| ***ZFHX3*** | 79 | chr16:g.72828033C>T | NM_006885.3 | c.8548G>A | p.(Glu2850Lys) | Unknown Significance | 5 | **3** |
|  | 195 | chr16:g.72845583G>A | NM_006885.3 | c.3757C>T | p.(Arg1253Cys) | Likely Pathogenic | 5 | **4** |
|  | | | | | | | | |
| ***X-linked*** | | | | | | | | |
| ***KCND1*** | 40 | chrX:g.48822720C>A | NM_004979.4 | c.1468-8G>T |  | Likely Pathogenic | 1 | **3** |
|  | 74 | chrX:g.48820012G>A | NM_004979.4 | c.1774C>T | p.(Arg592Trp) | Likely Pathogenic | 1 | **3** |
| ***PLXNA3*** | 77 | chrX:g.153692369G>C | NM_017514.3 | c.1623G>C | p.(Gln541His) | Likely Pathogenic° | 5 | **4°** |
|  | 112 | chrX:g.153688579G>A | NM_017514.3 | c.56G>A | p.(Gly19Asp) | Likely Pathogenic° | 5 | **4°** |
| *: variant or its segregation could not be validated by Sanger Sequencing; °variant was reclassified from Unknown Significance to Likely Pathogenic based on *in silico* structural analysis. | | | | | | | | |

| Supplementary Table 8. Overview of mutations in high-confidence candidate genes and affected individuals' phenotypes | | | | | | | | | | | | | | |
| --- | --- | --- | --- | --- | --- | --- | --- | --- | --- | --- | --- | --- | --- | --- |
| Sample | | 57 | | 154 | | 230 | | 165 | | 192 | | 125 | 152 | 208 |
| Gene | | ***CPZ* (NM_003652.3)** | | | | | | ***EDEM3*** **(NM_025191.3)** | | | | ***FBRS* (NM_001105079.1)** | | |
| Mutation(s) | | c.460C>T, p.(Arg154Trp) | | c.460C>T, p.(Arg154Trp) | | c.1288G>T, p.(Gly430Trp) | | c.1204T>G,  p.(Leu402Val) | | c.2431G>T,  p.(Asp811Tyr) | | c.577G>A,  p.(Gly193Ser) | c.1336C>G,  p.(Pro446Ala) | c.1034C>T,  p.(Pro345Leu) |
| Inheritance | | autosomal dominant | | | | | | autosomal dominant | | | | autosomal dominant | | |
|  | | *de novo* | | maternal | | dominant^1*^ | | *de novo* | | maternal | | *de novo* | maternal | dominant^3^ |
| Age | | 9 mo | | 13 mo | | 4 y | | 10 y 2 mo | | 4 y 1 mo | | 14 y | 20 mo | 3 y 5 mo |
| Gender | | male | | female | | male | | Male | | female | | male | female | male |
| SGA | | no | | yes | | no | | No | | no | | no | no | yes |
| Height (SDS) | |  | |  | |  | |  | |  | |  |  |  |
| Affected individual | | -3.0 | | -4.0 | | -1.7 | | -2.0 | | -2.0 | | -2.6 | -2.1 | -1.6 |
| Mother | | -1.0 | | -3.7 | | -1.2 | | -1.0 | | -2.1 | | -2.1 | -2.0 | 1.3 |
| Father | | -1.2 | | 1.3 | | -0.7 | | 0.3 | | 0.4 | | -1.3 | 0.3 | 1.2 |
| Weight (SDS) | | -2.7 | | -1.9 | | -2.3 | | -0.6 | | -1.2 | | -3.7 | 0.1 | -1.2 |
| OFC (SDS) | | -1.9 | | -1.1 | | -3.6 | | -0.7 | | -2.5 | | -3.2 | -2.23 | -1.5 |
| Dysproportionate SOS | | no | | no | | no | | no | | no | | no | no | no |
| Syndromic SOS | | no | | no | | no | | no | | no | | no | no | no |
| Bone age | | na | | na | | na | | delayed | | na | | na | na | na |
| IGF-1 (SDS) | | -1.1 | | na | | na | | 0.5 | | na | | na | na | na |
| IGFBP3 (SDS) | | -0.7 | | na | | na | | -0.5 | | na | | na | na | na |
| IQ | | normal | | normal | | normal | | normal | | normal | | normal | normal | normal |
| Clinical features | | | | | | | | | | | | | | |
| Head | | Wide nasal bridge, short neck | | short neck, midfacial hypoplasia | |  | |  | |  | |  | Thin hair | Thin hair |
| Eyes | | Hypertelorism | | Hypertelorism | |  | | High arched eye brows | | High arched eye brows | |  |  |  |
| Ears | | Low set ears | | Low set ears | | Low set ears, preauricular pits | |  | |  | |  | Prominent ears | Prominent ears |
| Mouth | | Thin upper lip | |  | | Thin upper lip | | Thin upper lip | | Thin upper lip | | Thin upper lip | Retrognathia | Thin lips |
| Thorax | | wide intermammillary distance | | wide intermammillary distance | |  | |  | |  | |  |  |  |
| Hand/Feet | | Brachydactyly | | broad thumb | | Brachydactyly | |  | | Limited supination of forearm | | Brachydactyly |  | Brachydactyly |
| Genitalia | |  | |  | |  | |  | |  | |  |  |  |
| Other | | Feeding difficulties | |  | |  | |  | |  | |  |  |  |
| ^#^ after GH treatment, *variant could not be validated by Sanger sequencing ^1^ no parental DNA available, ^2^ no maternal DNA available, ^3^ no paternal DNA available | | | | | | | | | | | | | | |
| **Supplementary Table 8 cont.** | | | | | | | | | |  |  |  |  |  |
| Sample | | 166 | | 188 | | 40 | | 74 | |  |  |  |  |  |
| Gene | | ***IFT81* (NM_014055.3)** | | | | ***KCND1*** **(NM_004979.4)** | | | |  |  |  |  |  |
| Mutation(s) | | c.259C>T,  p.(Arg87Cys) | | c.1150C>T,  p.(Arg384Cys) | | c.1468-8G>T | | c.1774C>T,  p.(Arg592Trp) | |  |  |  |  |  |
| Inheritance | | autosomal dominant | | | | X-linked | | | |  |  |  |  |  |
|  | | *de novo* | | paternal | | maternal | | maternal | |  |  |  |  |  |
| Age | | 13 y | | 11 y | | 4 y 4 mo | |  | |  |  |  |  |  |
| Gender | | male | | male | | male | | male | |  |  |  |  |  |
| SGA | | no | | yes | | yes | | no | |  |  |  |  |  |
| Height (SDS) | |  | |  | |  | |  | |  |  |  |  |  |
| Affected individual | | -2.9 | | -2.6 | | -2.4 | | -2.6 | |  |  |  |  |  |
| Mother | | -1.9 | | -1.1 | | -1.7 | | -1.0 | |  |  |  |  |  |
| Father | | -1.0 | | -2.2 | | -1.0 | | 0.0 | |  |  |  |  |  |
| Weight (SDS) | | -4.0 | | -4.5 | | -2.7 | | -1.3 | |  |  |  |  |  |
| OFC (SDS) | | -4.1 | | -2.1 | | -3.4 | | -1.3 | |  |  |  |  |  |
| Dysproportionate SOS | | no | | no | | no | | no | |  |  |  |  |  |
| Syndromic SOS | | no | | no | | no | | no | |  |  |  |  |  |
| Bone age | | na | | normal | | delayed | | normal | |  |  |  |  |  |
| IGF-1 (SDS) | | na | | 0.1 | | -0.8 | | -1.2 | |  |  |  |  |  |
| IGFBP3 (SDS) | | na | | 0.1 | | -0.3 | | -0.2 | |  |  |  |  |  |
| IQ | | normal | | normal | | normal | | normal | |  |  |  |  |  |
| Clinical features | | | | | | | | | |  |  |  |  |  |
| Head | | wide nasal bridge | | wide nasal bridge | | Fair hair | | Fair hair | |  |  |  |  |  |
| Eyes | | High arched eye brows | | High arched eye brows | | Sparse eye brows | | Hypertelorism, Sparse eye brows | |  |  |  |  |  |
| Ears | |  | |  | |  | |  | |  |  |  |  |  |
| Mouth | |  | | Cleft palate | | Thin lips | | Thin lips | |  |  |  |  |  |
| Thorax | |  | |  | |  | |  | |  |  |  |  |  |
| Hand/Feet | |  | |  | | Brachydactyly | | Brachydactyly | |  |  |  |  |  |
| Genitalia | |  | |  | |  | |  | |  |  |  |  |  |
| Other | |  | |  | |  | | Anal atresia | |  |  |  |  |  |
| ^#^ after GH treatment, ^1^ no parental DNA available, ^2^ no maternal DNA available, ^3^ no paternal DNA available | | | | | | | | | |  |  |  |  |  |

| **Supplementary Table 8 cont.** | | | | | | | | |
| --- | --- | --- | --- | --- | --- | --- | --- | --- |
| Sample | 44 | 162 | 167 | 189 | 207 | 214 | 219 | 226 |
| Gene | ***LAMA5* (NM_005560.4)** | | | | | | | |
| Mutation(s) | c.10433G>A,  p.(Ser3478Asn) | c.6101A>G,  p.(Asp2034Gly) | c.1249A>T,  p.(Asn417Tyr) | c.6301G>A,  p.(Glu2101Lys) | c.3244C>A,  p.(Pro1082Thr) | c.6226G>T,  p.(Ala2076Ser) | c.10322C>T,  p.(Thr3441Met) | c.3244C>A,  p.(Pro1082Thr) |
| Inheritance | autosomal dominant | | | | | | | |
|  | *de novo* | maternal | maternal | paternal | dominant^3^ | dominant^1^ | dominant^3^ | dominant^3^ |
| Age | 9 y | 8 y 10 mo | 4 y 4 mo | 10 y | 12 y 9 mo | 6 y 10 mo | 14 y | 11 y 6 mo |
| Gender | male | female | female | female | female | male | female | male |
| SGA | yes | no | no | no | no | yes | no | no |
| Height (SDS) |  |  |  |  |  |  |  |  |
| Affected individual | -2.4 | -2.4 | -3.4 | -1.7 | -2.5 | -3.0 | -3.0 | -2.3 |
| Mother | -0.3 | -3.0 | -3.4 | -2.0 | na | -0.1 | -0,8 | -0.4 |
| Father | 0.0 | -0.3 | -1.2 | -2.2 | na | -0.3 | 0.0 | -0.9 |
| Weight (SDS) | -1.5 | 0.3 | -4.3 | 1.6 | -2.6 | -2.6 | -2.1 | -1.7 |
| OFC (SDS) | -0.8 | -1.1 | -3.2 | -0.8 | 0.1 | -1.1 | -0.3 | -2.9 |
| Dysproportionate SOS | no | no | no | no | no | no | no | no |
| Syndromic SOS | no | no | no | no | yes | yes | no | no |
| Bone age | na | normal | na | accelerated | na | delayed | delayed | delayed |
| IGF-1 (SDS) | na | 2.1 | -1.0 | 0.7 | na | -1.5 | -0.1 | -2.0 |
| IGFBP3 (SDS) | na | 0.9 | -1.2 | -0.7 | na | -0.8 | -0.8 | 0.1 |
| IQ | normal | normal | normal | normal | 70-85 | 70-85 | normal | normal |
|  | | | | | | | | |
| Head |  | Short neck |  |  |  |  |  |  |
| Eyes |  |  |  | Hypertelorism |  |  |  |  |
| Ears |  |  | Posterior rotated |  |  |  |  |  |
| Mouth | Thin lips | Thin upper lip |  | Thin upper lip |  | Thin upper lip |  |  |
| Thorax |  | Barrel chest | Barrel chest | Barrel chest |  | Barrel chest |  | Barrel chest |
| Hand/Feet | Sandal gap | Sandal gap | Sandal gap | Sandal gap |  | Sandal gap |  | Sandal gap |
| Genitalia |  |  |  |  |  |  |  |  |
| Other |  |  |  | Obesity |  |  |  |  |
| ^#^ after GH treatment, ^1^ no parental DNA available, ^2^ no maternal DNA available, ^3^ no paternal DNA available | | | | | | | | |

| **Supplementary Table 8 cont.** | | | | | | |
| --- | --- | --- | --- | --- | --- | --- |
| Sample | 129 | 156 | 77 | 112 | 13 | 195 |
| Gene | ***MED24* (NM_014815.3)** | | ***PLXNA3* (NM_017514.3)** | | ***RASA3* (NM_007368.2)** | |
| Mutation(s) | c.1247C>G,  p.(Thr416Ser) | c.2288G>A,  p.(Arg763Gln) | c.1623G>C,  p.(Gln541His) | c.56G>A,  p.(Gly19Asp) | c.892_895del,  p.(Asp298Ilefs*10) | c.254T>C,  p.(Val85Ala) |
| Inheritance | autosomal dominant | | X-linked | | autosomal dominant | |
|  | *de novo* | maternal | maternal | maternal | *de novo* | *de novo* |
| Age | 5 y 10 mo | 12 y 5 mo | 10 y | 9 y 7 mo | 10 y 6 mo | 14 y 1 mo |
| Gender | male | male | male | male | female | female |
| SGA | no | no | no | yes | no | no |
| Height (SDS) |  |  |  |  |  |  |
| Affected individual | -2.1 | -2.3 | -1.7^#^ | -2.1 | -2.2 | -2.4 |
| Mother | -1.5 | -1.1 | 0.6 | -0.4 | -1.1 | -0.8 |
| Father | -1.7 | 0.8 | -0.6 | -0.6 | 0.9 | -1.2 |
| Weight (SDS) | 0.1 | -1.9 | 0.0 | -1.3 | -1.8 | -1.0 |
| OFC (SDS) | -1.2 | -1.8 | 0.2 | -1.5 | -2.0 | -2.0 |
| Dysproportionate SOS | no | no | no | no | no | no |
| Syndromic SOS | no | no | no | no | no | no |
| Bone age | delayed | delayed | na | normal | na | accelerated |
| IGF-1 (SDS) | -0.4 | -1.0 | na | -2.4 | na | 0.8 |
| IGFBP3 (SDS) | -0.1 | -1.2 | na | -1.9 | na | 1.0 |
| IQ | normal | normal | normal | normal | normal | normal |
|  | | | | | | |
| Head |  |  | Cow-lick |  | Short neck, broad nasal tip | broad nasal tip |
| Eyes |  |  | Lateral hypoplasia of brows | Lateral hypoplasia of brows |  |  |
| Ears |  |  |  |  |  |  |
| Mouth | Thick lips | Thin lips |  |  | Thin upper lip | Thin upper lip |
| Thorax |  |  |  |  | Barrel chest | Barrel chest |
| Hand/Feet | Brachydactyly | Brachydactyly | Broad thumbs, sandal gap | Broad thumbs |  | Sandal gap, brachydactyly |
| Genitalia |  |  |  |  |  |  |
| Other |  |  | Scoliosis, hypoplastic patella |  | Café-au-lait spot |  |
| ^#^ after GH treatment, ^1^ no parental DNA available, ^2^ no maternal DNA available, ^3^ no paternal DNA available | | | | | | |

| **Supplementary Table 8 cont.** | | | | | | | | |
| --- | --- | --- | --- | --- | --- | --- | --- | --- |
| Sample | 36 | 159 | 60 | 184 | 200 | 205 | 237 | 247 |
| Gene | ***SLC7A8* (NM_001267036.1)** | | ***UBR4* (NM_020765.2)** | | | | | |
| Mutation(s) | c.536A>G,  p.(Asn179Ser) | c.415G>A,  p.(Ala139Thr) | c.7031A>G,  p.(Asn2344Ser) | c.4045C>G,  p.(Arg1349Gly) | c.10388G>A,  p.(Arg3463His) | c.14237C>T,  p.(Pro4746Leu) | c.6325G>A,  p.(Gly2109Ser) | c.13828C>A,  p.(Pro4610Thr) |
| Inheritance | autosomal dominant | | autosomal dominant | | | | | |
|  | *de novo* | maternal | *de novo* | maternal | dominant^3^ | dominant^3^ | dominant^1^ | dominant^1^ |
| Age | 5 y 9 mo | 10 y 5 mo | 11 y | 8 y | 13 mo | 13 y 8 mo | 36 y | 6 y |
| Gender | female | female | female | female | female | male | female | female |
| SGA | no | no | no | no | yes | no | na | no |
| Height (SDS) |  |  |  |  |  |  |  |  |
| Affected individual | -3.4 | -2.2 | -2.6 | -3.6 | -3.0 | -2.6 | -3.6 | -2.3 |
| Mother | -0.4 | -2.7 | 0.6 | -2.2 | -3.3 | -0.1 | 0.9 | na |
| Father | 0.3 | -1.2 | -1.2 | -2.6 | -0.8 | -1.6 | 0.2 | na |
| Weight (SDS) | -1.6 | -0.8 | -0.6 | -2.1 | -2.4 | -2.1 | -2.4 | -2.5 |
| OFC (SDS) | 0.3 | -0.4 | 0.5 | -0.1 | -0.9 | -2.5 | 0.1 | -1.6 |
| Dysproportionate SOS | no | no | no | no | no | no | no | no |
| Syndromic SOS | no | no | no | no | yes | yes | no | yes |
| Bone age | na | accelerated | delayed | delayed | Normal | na | na | na |
| IGF-1 (SDS) | 0.0 | na | na | -1.2 | na | na | na | na |
| IGFBP3 (SDS) | 0.7 | na | na | -1.6 | na | na | na | na |
| IQ | normal | normal | normal | normal | 70-85 | 70-85 | normal | 70-85 |
|  | | | | | | | | |
| Head |  | Flat nasal bridge |  |  | Frontal bossing |  |  |  |
| Eyes |  |  | Lateral sparse brows | Lateral sparse brows | Lateral sparse brows |  | Lateral sparse brows |  |
| Ears | Posterior rotated ears |  |  |  |  |  |  |  |
| Mouth |  |  |  |  |  |  |  |  |
| Thorax |  |  | Barrel chest | Barrel chest | Barrel chest |  | Barrel chest |  |
| Hand/Feet | Brachydactyly | Brachydactyly, Syndactyly of three fingers | Brachydactyly | Brachydactyly |  | Brachydactyly | Brachydactyly | Brachydactyly, sandal gap |
| Genitalia |  |  |  |  |  |  |  |  |
| Other |  |  |  |  |  |  |  |  |
| ^#^ after GH treatment, ^1^ no parental DNA available, ^2^ no maternal DNA available, ^3^ no paternal DNA available | | | | | | | | |

| **Supplementary Table 8 cont.** | | | | | | |
| --- | --- | --- | --- | --- | --- | --- |
| Sample | 135 | 156 | 157 | 171 | 79 | 195 |
| Gene | ***USP45* (NM_001080481.1)** | | | | ***ZFHX3* (NM_006885.3)** | |
| Mutation(s) | c.1567A>G,  p.(Ser523Gly) | c.1990G>T,  p.(Gly664*) | c.727G>A,  p.(Val243Met) | c.178T>G,  p.(Ser60Ala) | c.8548G>A,  p.(Glu2850Lys) | c.3757C>T,  p.(Arg1253Cys) |
| Inheritance | autosomal dominant | | | | autosomal dominant | |
|  | *de novo* | maternal | maternal | maternal | *de novo* | *de novo* |
| Age | 2 y 7 mo | 12 y 5 mo | 2 y 11 mo | 5 y | 18 mo | 14 y 1 mo |
| Gender | female | male | female | female | female | female |
| SGA | no | no | no | no | no | no |
| Height (SDS) |  |  |  |  |  |  |
| Affected individual | -3.0 | -2.3 | -1.7^#^ | -2.8 | -1.8 | -2.4 |
| Mother | -0.2 | -1.1 | -1.5 | -1.6 | 1.9 | -0.8 |
| Father | -1.8 | 0.8 | 1.0 | 0.0 | -0.3 | -1.2 |
| Weight (SDS) | -1.9 | -1.9 | -0.6 | -2.3 | -1.5 | -1.0 |
| OFC (SDS) | -0.8 | -1.8 | -0.5 | -3.3 | -2.0 | -2.0 |
| Dysproportionate SOS | no | no | no | no | no | no |
| Syndromic SOS | no | no | no | no | no | no |
| Bone age | na | delayed | accelerated | delayed | na | accelerated |
| IGF-1 (SDS) | -1.1 | -1.0 | 3.0 | -1.8 | -0.3 | 0.8 |
| IGFBP3 (SDS) | -0.3 | -1.2 | 0.6 | 0.4 | -0.2 | 1.0 |
| IQ | normal | normal | normal | normal | normal | normal |
|  | | | | | | |
| Head |  |  |  |  | Fine hair | broad nasal tip |
| Eyes |  |  |  |  | Sparse brows |  |
| Ears |  |  |  |  |  |  |
| Mouth |  | Thin lips |  |  | Thin upper lip | Thin upper lip |
| Thorax |  |  |  |  | Barrel chest | Barrel chest |
| Hand/Feet | Brachydactyly | Brachydactyly | Brachydactyly | Brachydactyly | Brachydactyly | Sandal gap, brachydactyly |
| Genitalia |  |  |  |  | Fused labia |  |
| Other |  |  |  |  | Patchy spots, hypotonia |  |
| ^#^ after GH treatment, ^1^ no parental DNA available, ^2^ no maternal DNA available, ^3^ no paternal DNA available | | | | | | |

| Supplementary Table 9. Brief description of high-confidence candidate genes with respect to short stature | |
| --- | --- |
| ***CPZ* (carboxypeptidase Z)**.  *CPZ* encodes a metallocarboxypeptidase.^71^ It was reported that induction of CPZ by thyroid hormones modulates Wnt signaling pathways by modification of the activity of Wnt-4 and thereby regulates the terminal differentiation of growth plate chondrocytes.^72^ Moreover, *CPZ* is expressed in the somites of early chicken embryo and subsequently in the sclerotome.^73^ Mutations in genes acting in the Wnt signaling pathway have been reported to cause short stature in human.^74^ A SNP (rs2302580) in LD to *CPZ* was reported to be one of the 697 SNPs significantly associated with adult human height.^33^  ***EDEM3* (ER degradation enhancing alpha-mannosidase like protein 3)**.  *EDEM3* was reported to be involved in endoplasmic reticulum-associated degradation of glycoproteins by acting in the second step of mannose trimming.^75; 76^ In a model for prostate cancer (LNCaP cells), it was reported that *EDEM3* expression was regulated by androgen.^77^ Moreover, EDEM3 was reported to be upregulated in prostate cancer. ^77^ By siRNA depletion, it was further shown that *EDEM3* expression plays a role in viability and growth of prostate cancer cells. ^77^ Its action in cancer growth might imply a possible contribution to cellular growth regulation. Furthermore, a 20.95 Mb deletion with unknown pathogenic contribution encompassing *EDEM3* in an affected individual presenting with short stature was reported in decipher.^39^  ***FBRS* (fibrosin)**.  *FBRS* encodes a lymphokine.^78^ Only little is known about the function of fibrosin. Until now, research was focused on its activities in wound healing.^79^ In decipher, an affected individual with a 3.3 Mb deletion including *FBRS* presents with short stature and sparse hair.^39^  ***IFT81* (intraflagellar transport protein 81)**.  *IFT81* encodes a member of the IFT complex B core.^80^ Together with IFT74, IFT81 is required for ciliogenesis.^81^ Recessive mutations in *IFT81* have been reported to cause ciliopathies, especially short rib polydactyly syndrome, which includes severe short stature reported by a reduced birth length (38cm and 42.5 cm).^82; 83^ Parental height was not particularly reported in the articles. Duran et al. analyzed the effect of disrupted IFT81 and found out that IFT-B complex members are destabilized at protein level, acetylated tubulin is increased (indicating impaired turnover of tubulin in the cilium), cilia length is increased, Hedgehog signaling is dysregulated and the growth plate is histologically altered.^83^ In contrast, Perrault et al. reported a reduced abundance of cilia and a tendency towards reduced cilia length in the affected individual. ^82^  ***KCND1* (potassium voltage-gated channel subfamily D member 1)**  *KCND1* or *Kv4.1* encodes a member of the voltage-gated K^+^ channel family.^84-86^ 6 affected individuals from decipher with CNVs encompassing *KCND1* (2 of which are reported to be definitely pathogenic) were reported to be affected from short stature.^39^ Moreover, *KCND1* is expressed in the thyroid gland which might imply a function in the hormonal regulation of growth.^87^  ***LAMA5* (laminin subunit alpha 5).**  *LAMA5* encodes the laminin subunit alpha 5 and is an extracellular matrix molecule, or specifically one of the main α-chains in the epithelial basement membrane.^88^ It was reported to play a crucial role in development.^88^ Moreover, it regulates Wnt- and PI3K signaling^89^ and was reported to be upregulated during osteoblast differentiation in MC3T3-E1 cells.^90^ Although some groups report about its involvement in syndromes including disruption of physiological kidney function^91^ or a multisystem syndrome including osteoarthritis and hypothyroidism^92^, no information on height of the affected individuals is reported. But de Luca et al. could find *LAMA5* to be associated with shorter stature in elderly individuals from Southern Italy.^93; 94^ In line with that, apart from other defects, Lama5^neo/neo^ mice are smaller than controls.^95^ A 3.63 Mb duplication with unknown pathogenic contribution encompassing *LAMA5* in an affected individual presenting with short stature was reported in decipher.^39^  ***MED24* (mediator of RNA polymerase II transcription subunit 24).**  *MED24*, also known as *TRAP100*, encodes one of the thyroid hormone receptor-associated proteins, that forms a complex with the thyroid receptor via TRAP220.^96^ Thereby it enhances transcription during TRAP-mediated coactivation.^96^ Thus, it is involved in thyroid hormone signaling.^96; 97^ In zebrafish, it was reported to be relevant for ENS development potentially by influencing proliferation of ENS precursors^98^ whereas in drosophila, it was shown to act in control of cell death^99^ In decipher, a 2.39 Mb deletion with likely pathogenic contribution encompassing *MED24* was reported in an affected individual presenting with short stature. ^39^ In Trap100^-/-^ mouse model, a small embryo size was reported. ^100^ | **PLXNA3 (plexin A3).**  *PLXNA3* encodes a semaphorin receptor.^101^ Until now, studies were mostly focused on its function in the nervous system indicating its role in synapse development, neuronal cell death or growth of primary motor axons.^102-105^. Nonetheless, it is encompassed by 9 CNVs in affected individuals with short stature according to decipher database, 3 of which were assigned to be definitely pathogenic.^39^ In zebrafish, plxna3 is expressed in skeletal precursor cells and knockdown of plxna3 causes short segments and reduced fin length, taken together pointing to a potential contribution to joint formation.^106^ According to STRING DB, PLXNA3 interacts with Semaphorin-3A and was further reported to mediate signaling of SEMA3A.^42; 103^ Biallelic mutations in SEMA3A were shown to be causative for syndromes including short stature^107; 108^.  **RASA3 (ras GTPase-activating protein 3).**  *RASA3* encodes a Ras-GTPase activating protein and thus part of the RAS MAPK pathway. ^109^ This pathway is implicated in several syndromes including short stature, for example noonan syndrome and recent publications even report other RASA complex genes to be involved in the Noonan-syndrome spectrum.^110^ Furthermore, three CNVs (two gains and one deletion) overlapping *RASA3* were reported in decipher in affected individuals with short stature and a RASA3 ortholog mouse model showed a growth phenotype.^39; 111^  ***SLC7A8* (large neutral amino acids transporter small subunit 2 isoform c)**  *SLC7A8* encodes a constituent of the multimeric complex mediating neutral and cationic amino acid transport and cysteine/glutamate exchange.^112^ According to KEGG database, the protein is involved in protein digestion and absorption.^113-115^ Moreover, it was reported that it further activates the mTORC1 pathway which is known to influence cell growth and proliferation.^116^ Due to decipher, *SLC7A8* is encompassed by 1 CNV assigned to be definitively pathogenic in an affected individual with short stature.^39^ Heterozygous mutations in *SLC7A8* potentially cause age-related hearing loss, whereby affected individual’s height was not reported in the study.^117^ In contrast, recessive mutations in another glycoprotein-associated amino acid transporter^118^, encoded by *SLC7A7*, were reported to cause lysinuric protein intolerance, a disease including short stature as a common phenotypic feature resulting secondarily from poor feeding, vomiting and diarrhea (reviewed by ^119^). *SLC7A8* shares sequence homology and expression profile with *SLC7A7*.^120^  ***UBR4* (ubiquitin protein ligase E3 component n-recognin 4)**  *UBR4* encodes a N-recognin and contains an UBR box motif.^121^ It has been reported to be involved in growth of cancer cells.^122^ For missense variants, *UBR4* has a Z-score of 5.98 according to ExAC, indicating their potential pathogenic relevance.^7^ Moreover, a 6.62 Mb duplication with unknown pathogenic contribution and a 3.46 Mb deletion with a pathogenic effect encompassing *UBR4* were reported in decipher in two affected individuals presenting with short stature.^39^ In mice, a biallelic knockout of the gene led to embryonic death implying that homozygous mutations in this gene might not be compatible with life.^123^ Despite, until embryonic death, growth retardation was reported in the fetuses of knockout mice.^123^ Moreover, mutations in UBR1, another E3 ubiquitin ligase, were reported to cause Jonathan-Blizzard syndrome, which also includes short stature. ^124^  ***USP45* (ubiquitin carboxyl-terminal hydrolase 45)**  Ubiquitin-specific proteases (USPs) hydrolyse linkages with only modest ubiquitin chain preferences.^125^ A 12.17 Mb deletion with unknown pathogenic contribution encompassing *USP45* in an affected individual presenting with short stature as well as brachydactyly was reported in decipher.^39^ According to STRING DB, USP45 interacts with ERCC1 that acts in the nucleotide excision repair.^42^ Compound heterozygous mutations in *ERCC1* in an affected individual caused cerebro-oculo-facial-skeletal syndrome including failure to thrive (56cm at 14 month).^126^ Another affected individual with a homozygous mutation in *ERCC1* was reported to present with cockayne syndrome type II. ^127^ His height was not reported but the syndrome is known to include growth failure (as reviewed in ^128^). USP45 was reported to deubiquitylate ERCC1 in vitro.^129^  ***ZFHX3* (zinc finger homeobox protein 3).**  *ZFHX3* or *ATBF1* (AT motif binding factor 1) encodes a transcription factor with four homeodomains and 17 zinc fingers.^130-132^ One affected individual with proportionate short stature was reported in decipher to present with a deletion of 1.19 Mb assigned to be definitely pathogenic that encompasses *ZFHX3*.^39^ *Atbf1^wt/Δ7&8^* mice with a heterozygous deletion of exon 7 and 8 of ZFHX3 were much smaller than their wildtype littermates at day 10 after birth.^133^ Furthermore, ZFHX3 was shown to influence STAT3 signaling via its interaction to PIAS3.^134; 135^ Heterozygous gain-of- function mutations in *STAT3* have been reported to affect intracellular growth hormone signaling and thus cause growth failure in affected individuals (reviewed in ^136^). Moreover, ZFHX3 was reported to directly interact with POU1F1 and to be necessary for early POU1F1 transcriptional activation.^137^ A mutation in *POU1F1*, in turn, was reported to cause isolated growth hormone deficiency and short stature in affected individuals.^138^ |

| Supplementary Table 10. KEGG & REACTOME pathways of high-confidence candidate genes | | | |
| --- | --- | --- | --- |
| High-confidence  candidate gene | KEGG pathway  [No. of genes in pathway] | No. of growth related genes^+^ in KEGG pathway [% of all genes in the respective pathway] | Growth related genes^+^ in KEGG pathway |
| *CPZ* | - | - | *-* |
| *EDEM3* | **Protein processing in endoplasmic reticulum - Homo sapiens (human), hsa04141 [165]** | 9 [5.5] | *DNAJC3, EIF2AK3, MBTPS2, SAR1B, SEC23A, SIL1, STT3B, UFD1L, WFS1* |
| *FBRS* | - | - | *-* |
| *IFT81** | **Cilium Assembly, organism-specific biosystem, REACTOME, R-HSA-5617833, [201]** | 54 [26.9] | *ALMS1, ARL6, BBIP1, BBS1, BBS10, BBS12, BBS2, BBS4, BBS5, BBS7, BBS9, CC2D2A, CDK5RAP2, CENPJ, CEP135, CEP152, CEP164, CEP290, CEP57, CEP63, DYNC1H1, DYNC2H1, HDAC6, IFT122, IFT140, IFT172, IFT27, IFT43, IFT80, IQCB1, LZTFL1, MKKS, MKS1, NDE1, NPHP1, NPHP3, NPHP4, OFD1, PCNT, PDE6D, PLK4, RPGRIP1L, SDCCAG8, TCTN3, TMEM216, TMEM67, TRIP11, TTC21B, TTC8, TUBB4A, WDR19, WDR34, WDR35, WDR60* |
|  | Intraflagellar transport, organism-specific biosystem, REACTOME, R-HSA-5620924, [53] | 14 [26.4] | *DYNC2H1, IFT122, IFT140, IFT172, IFT27, IFT43, IFT80, TRIP11, TTC21B, TUBB4A, WDR19, WDR34, WDR35, WDR60* |
|  | Organelle biogenesis and maintenance, organism-specific biosystem, REACTOME R-HSA-1852241 [297] | 59 [19.9] | *ALMS1, ARL6, ATP5A1, BBIP1, BBS1, BBS10, BBS12, BBS2, BBS4, BBS5, BBS7, BBS9, C10orf2, CC2D2A, CDK5RAP2, CENPJ, CEP135, CEP152, CEP164, CEP290, CEP57, CEP63, CREBBP, DYNC1H1, DYNC2H1, HCFC1, HDAC6, IFT122, IFT140, IFT172, IFT27, IFT43, IFT80, IQCB1, LZTFL1, MEF2C, MKKS, MKS1, NDE1, NPHP1, NPHP3, NPHP4, OFD1, PCNT, PDE6D, PLK4, RPGRIP1L, SDCCAG8, TCTN3, TMEM216, TMEM67, TRIP11, TTC21B, TTC8, TUBB4A, WDR19, WDR34, WDR35, WDR60* |
| *KCND1** | **Cardiac conduction, REACTOME, R-HSA-5576891 [140]** | 11 [7.9] | *GATA4, KCNJ11, KCNJ2, NKX2-5, NPR2, RYR1, SCN4A, SCN9A, STIM1, TBX5, WWTR1* |
|  | Muscle contraction, REACTOME, R-HSA-397014 [205] | 16 [7.8] | *GATA4, KCNJ11, KCNJ2, MYH3, MYH8, NKX2-5, NPR2, RYR1, SCN4A, SCN9A, STIM1, TBX5, TNNI2, TNNT3, TPM2, WWTR1* |
|  | Neuronal System, REACTOME, R-HSA-112316 [370] | 17 [4.6] | *ABCC8, BRAF, CASK, CHRNA1, CHRND, CHRNG, COMT, GRIA3, GRM1, HRAS, KCNJ1, KCNJ10, KCNJ11, KCNJ2, RAF1, RPS6KA3, SLC38A1* |
|  | Phase 1 - inactivation of fast Na+ channels, REACTOME, R-HSA-5576894 [7] | 0 [0.0] |  |
|  | Potassium Channels, REACTOME, R-HSA-1296071 [99] | 5 [5.1] | *ABCC8, KCNJ1, KCNJ10, KCNJ11, KCNJ2* |
|  | Voltage gated Potassium channels, REACTOME, R-HSA-1296072 [43] | 0 [0.0] |  |
| *LAMA5* | ECM-receptor interaction,  hsa04512 [83] | 10 [12.0] | *COL1A1, COL1A2, COL2A1, COL6A2, COL9A1, COL9A2, COL9A3, COMP, GP1BB, HSPG2* |
|  | Metabolic pathways, hsa01100 [1220] | 150 [12.3] | *AASS, ACACA, ACACA, ACP5, ADSL, AGK, AGK, AGL, AGPS, ALDH18A1, ALDH3A2, ALDOA, ALG1, ALOX12B, ALPL, AMPD2, ARG1, ARSB, ASAH1, ASL, ATP5A1, ATP6V0A2, ATP6V0A4, ATP6V1B1, AUH, B3GALT6, B4GALT7, CERS3, CHSY1, COMT, COX10, COX15, COX6B1, COX7B, CYP11B1, CYP19A1, CYP21A2, CYP21A2, CYP21A2, CYP21A2, CYP21A2, CYP21A2, CYP2R1, DGUOK, DHCR24, DHCR7, DHODH, DNMT3B, DOLK, DPYD, DPYS, EBP, EXT1, EXT2, G6PC, G6PC3, G6PD, GALNS, GBA, GBA, GCK, GK, GLB1, GLYCTK, GMPPA, GUSB, GYS2, HADH, HMOX1, HPRT1, HYAL1, IDH1, IDS, IDUA, IMPAD1, LARGE, LIAS, MLYCD, MOCS2, MTR, MVK, NDST1, NDUFA1, NDUFA10, NDUFA10, NDUFA11, NDUFA12, NDUFA2, NDUFA9, NDUFB3, NDUFB9, NDUFS1, NDUFS1, NDUFS2, NDUFS3, NDUFS3, NDUFS4, NDUFS6, NDUFS7, NDUFS8, NDUFV1, NDUFV2, NSDHL, NT5C2, OCRL, OGDH, PAPSS2, PCCA, PCCB, PCYT1A, PGK1, PGM1, PGM3, PIGL, PIGO, PKLR, PKLR, PMM2, PNPLA2, POLE, POLG, POLR3A, POLR3B, PPOX, PRPS1, PSAT1, PSPH, PTDSS1, PYCR1, PYGL, RDH11, SAT1, SC5D, SDHA, SDHB, SDHC, SDHD, SMPD1, SMS, SPR, SUCLA2, SUCLG1, TAT, TPO, TPO, UROC1, UROD, UROS, XYLT1, XYLT1* |
|  | PI3K-Akt signaling pathway, hsa04151 [352] | 46 [13.1] | *AKT1, BDNF, BRCA1, COL1A1, COL1A2, COL2A1, COL6A2, COL9A1, COL9A2, COL9A3, COMP, ERBB2, FGF10, FGF17, FGF23, FGF8, FGFR1, FGFR2, FGFR3, G6PC, G6PC3, GH1, GHR, GYS2, HRAS, IGF1, IGF1R, IGF2, IKBKG, IL2RG, INS, INSR, KIT, KRAS, MAP2K1, MAP2K2, NRAS, PDGFB, PDGFRB, PIK3CA, PIK3R1, PTEN, RAF1, SOS1, STK11, TP53* |
|  | Focal adhesion, hsa04510 [199] | 28 [14.0] | *ACTB, ACTG1, AKT1, BRAF, CAV1, COL1A1, COL1A2, COL2A1, COL6A2, COL9A1, COL9A2, COL9A3, COMP, CTNNB1, ERBB2, FLNA, FLNB, HRAS, IGF1, IGF1R, MAP2K1, PDGFB, PDGFRB, PIK3CA, PIK3R1, PTEN, RAF1, SOS1* |
|  | Toxoplasmosis, hsa05145 [113] | 9 [8.0] | *AKT1, CASP8, HLA-DQB1, IKBKG, STAT1, STAT3, TAB2, TGFB1, TGFB3* |
|  | Amoebiasis, hsa05146 [96] | 10 [10.4] | *ARG1, COL1A1, COL1A2, COL3A1, GNA11, GNAS, PIK3CA, PIK3R1, TGFB1, TGFB3* |
|  | **Human papillomavirus infection, hsa05165 [319]** | 46 [14.4] | *AKT1, ATM, ATP6V0A2, ATP6V0A4, ATR, CASP8, COL1A1, COL1A2, COL2A1, COL6A2, COL9A1, COL9A2, COL9A3, COMP, CREBBP, CTNNB1, EP300, GNAS, HDAC4, HDAC6, HDAC8, HES7, HRAS, IKBKG, JAG1, KRAS, LFNG, MAP2K1, MAP2K2, NOTCH1, NOTCH2, NOTCH3, NRAS, PDGFRB, PIK3CA, PIK3R1, PTEN, RAF1, SCRIB, SOS1, STAT1, TERT, TP53, WNT1, WNT5A, WNT7A* |
|  | Pathways in cancer, hsa05200 [526] | 67 [12.7] | *AKT1, BMP2, BRAF, BRCA2, CASP8, CBL, CREBBP, CTNNB1, DLL3, EDNRB, EP300, ERBB2, FGF10, FGF17, FGF23, FGF8, FGFR1, FGFR2, FGFR3, GLI2, GLI3, GNA11, GNAS, HMOX1, HRAS, IGF1, IGF1R, IGF2, IKBKG, IL2RG, JAG1, KIF7, KIT, KRAS, LRP5, MAP2K1, MAP2K2, MMP2, MMP9, NOTCH1, NOTCH2, NOTCH3, NRAS, PAX8, PDGFB, PDGFRB, PIK3CA, PIK3R1, PPARG, PTEN, RAF1, RARB, RET, SHH, SMAD4, SOS1, STAT1, STAT3, STAT5B, TERT, TGFB1, TGFB3, TP53, WNT1, WNT5A, WNT7A, ZBTB16* |
|  | Small cell lung cancer, hsa05222 [93] | 7 [7.5] | *AKT1, IKBKG, PIK3CA, PIK3R1, PTEN, RARB, TP53* |
| *MED24* | **Thyroid hormone signaling pathway - Homo sapiens (human), hsa04919 [116]** | 23 [19.8] | *ACTB, ACTG1, AKT1, CREBBP, CTNNB1, EP300, GATA4, HRAS, KRAS, MAP2K1, MAP2K2, MED12, NOTCH1, NOTCH2, NOTCH3, NRAS, PIK3CA, PIK3R1, RAF1, STAT1, THRA, THRB, TP53* |
| *PLXNA3* | **Axon guidance - Homo sapiens (human), hsa04360 [175]** | 13 [7.4] | *BMPR1B, EFNB1, HRAS, KRAS, L1CAM, NRAS, PIK3CA, PIK3R1, PTPN11, RAF1, SEMA3E, SHH, WNT5A* |
| *RASA3* | **Ras signaling pathway - Homo sapiens (human),**  **hsa04014 [232]** | 30 [12.9] | *AKT1, BDNF, FGF10, FGF17, FGF23, FGF8, FGFR1, FGFR2, FGFR3, HRAS, IGF1, IGF1R, IGF2, IKBKG, INS, INSR, KIT, KRAS, MAP2K1, MAP2K2, NF1, NRAS, PDGFB, PDGFRB, PIK3CA, PIK3R1, PTPN11, RAF1, SHOC2, SOS1* |
| *SLC7A8* | **Protein digestion and absorption - Homo sapiens (human),**  **hsa04974 [90]** | 18 [20.0] | *COL10A1, COL11A1, COL11A2, COL1A1, COL1A2, COL27A1, COL2A1, COL3A1, COL5A1, COL5A2, COL6A2, COL7A1, COL9A1, COL9A2, COL9A3, ELN, SLC6A19, SLC7A7* |
| *UBR4* | Viral carcinogenesis,  hsa05203 [201] | 16 [8.0] | *CASP8, CREBBP, EP300, HDAC4, HDAC6, HDAC8, HRAS, IKBKG, KRAS, NRAS, PIK3CA, PIK3R1, SCRIB, STAT3, STAT5B, TP53* |
|  | **Human papillomavirus infection, hsa05165 [319]** | 46 [14.4] | *AKT1, ATM, ATP6V0A2, ATP6V0A4, ATR, CASP8, COL1A1, COL1A2, COL2A1, COL6A2, COL9A1, COL9A2, COL9A3, COMP, CREBBP, CTNNB1, EP300, GNAS, HDAC4, HDAC6, HDAC8, HES7, HRAS, IKBKG, JAG1, KRAS, LFNG, MAP2K1, MAP2K2, NOTCH1, NOTCH2, NOTCH3, NRAS, PDGFRB, PIK3CA, PIK3R1, PTEN, RAF1, SCRIB, SOS1, STAT1, TERT, TP53, WNT1, WNT5A, WNT7A* |
| *USP45** | **DNA Repair, REACTOME, R-HSA-73894 [295]** | 50 [16.9] | *ACTB, ATM, ATR, ATRIP, BLM, BRCA1, BRCA2, BRCC3, BRIP1, CUL4B, DNA2, EP300, ERCC1, ERCC2, ERCC3, ERCC4, ERCC5, ERCC6, ERCC8, EYA1, FANCA, FANCB, FANCC, FANCD2, FANCE, FANCF, FANCG, FANCI, FANCL, FANCM, FTO, GTF2H5, HERC2, NBN, PALB2, PCNA, PNKP, POLE, PRKDC, RAD50, RAD51C, RBBP8, RNF168, RTEL1, SLX4, SPRTN, TP53, UFD1L, WHSC1, WRN* |
|  | Formation of Incision Complex in GG-NER, REACTOME, R-HSA-5696395 [43] | 7 [16.3] | *CUL4B, ERCC1, ERCC2, ERCC3, ERCC4, ERCC5, GTF2H5* |
|  | Global Genome Nucleotide Excision Repair (GG-NER), REACTOME, R-HSA-5696399 [84] | 10 [11.9] | *ACTB, CUL4B, ERCC1, ERCC2, ERCC3, ERCC4, ERCC5, GTF2H5, PCNA, POLE* |
|  | Nucleotide Excision Repair, REACTOME, R-HSA-5696398 [110] | 13 [11.8] | *ACTB, CUL4B, EP300, ERCC1, ERCC2, ERCC3, ERCC4, ERCC5, ERCC6, ERCC8, GTF2H5, PCNA, POLE* |
| *ZFHX3* | **Signaling pathways regulating pluripotency of stem cells - Homo sapiens (human),**  **hsa04550 [139]** | 29 [20.9] | *AKT1, BMPR1A, BMPR1B, CTNNB1, DLX5, FGFR1, FGFR2, FGFR3, HESX1, HRAS, IGF1, IGF1R, KRAS, LIFR, MAP2K1, MAP2K2, NODAL, NRAS, PAX6, PIK3CA, PIK3R1, RAF1, SMAD4, SOX2, STAT3, TBX3, WNT1, WNT5A, WNT7A* |
| * No KEGG pathway encompassing this gene; pathways from REACTOME were analyzed instead  Bold: main pathway, i.e. the pathway with the highest frequency of growth-related genes for each high-confidence candidate gene  ^+^: Growth related genes from the list in the Supplementary Tables | | | |

| Supplementary Table 11. Medium-confidence candidate genes | | | | | | | | |
| --- | --- | --- | --- | --- | --- | --- | --- | --- |
| Gene | Sample ID | Genomic position (GRCh37) | RefSeq number | cDNA level | Protein level | Variant Level Classification | Gene Level Classification | Combined Score |
| ***autosomal dominant*** | | | | | | | | |
| ***ANKS3*** | 145 | chr16:g.4747040dup | NM_133450.2 | c.1960dup | p.(Arg654Profs*25) | Pathogenic | 1 | **4** |
| ***BAI3^#^*** | 181 | chr6:g.70092745G>A | NM_001704.2 | c.4298G>A | p.(Arg1433Lys) | Likely Pathogenic | 0 | **2** |
|  | 189 | chr6:g.69759199T>C | NM_001704.2 | c.2294T>C | p.(Val765Ala) | Likely Pathogenic | 0 | **2** |
| ***BRD4*** | 88 | chr19:g.15366299C>A | NM_058243.2 | c.1856G>T | p.(Ser619Ile) | Likely Pathogenic | 5 | **4** |
| ***CEP76^#^*** | 28 | chr18:g.12673470C>T | NM_024899.3 | c.1874G>A | p.(Arg625His) | Likely Pathogenic | 2 | **3** |
|  | 184 | chr18:g.12701068T>C | NM_024899.3 | c.108A>G | p.(Ile36Met) | Unknown Significance | 2 | **2** |
|  | 239* | chr18:g.12686398C>T | NM_024899.3 | c.985G>A | p.(Ala329Thr) | Unknown Significance | 2 | **2** |
| ***CLGN^+^*** | 166 | chr4:g.141317285T>C | NM_004362.2 | c.959A>G | p.(Lys320Arg) | Likely Pathogenic | 1 | **3** |
|  | 225* | chr4:g.141320158T>C | NM_004362.2 | c.731A>G | p.(Asp244Gly) | Likely Pathogenic | 1 | **3** |
|  | 244* | chr4:g.141327129G>T | NM_004362.2 | c.386C>A | p.(Pro129Gln) | Likely Pathogenic | 1 | **3** |
| ***CLIC4*** | 140 | chr1:g.25140622C>T | NM_013943.2 | c.220C>T | p.(His74Tyr) | Likely Pathogenic | 5 | **4** |
| ***CLIP1*** | 139 | chr12:g.122862163G>A | NM_002956.2 | c.430C>T | p.(Arg144*) | Pathogenic | 4 | **4** |
| ***DENND4B^#^*** | 92 | chr1:g.153911516G>A | NM_014856.2 | c.1825C>T | p.(Leu609Phe) | Likely Pathogenic | 2 | **3** |
|  | 167 | chr1:g.153906724C>G | NM_014856.2 | c.2828G>C | p.(Trp943Ser) | Unknown Significance | 2 | **2** |
| ***DNAH17^#^*** | 108 | chr17:g.76521189C>T | NM_173628.3 | c.3766G>A | p.(Gly1256Arg) | Likely Pathogenic | 3 | **3** |
|  | 185 | chr17:g.76567414G>A | NM_173628.3 | c.779C>T | p.(Ala260Val) | Unknown Significance | 3 | **2** |
|  | 216* | chr17:g.76487631C>T | NM_173628.3 | c.6563G>A | p.(Arg2188Gln) | Likely Pathogenic | 3 | **3** |
| ***FGF18*** | 179 | chr5:g.170863238C>T | NM_003862.2 | c.211C>T | p.(Arg71Cys) | Likely Pathogenic | 6 | **4** |
| ***FZD2^#^*** | 166 | chr17:g.42635711C>T | NM_001466.3 | c.655C>T | p.(Arg219Cys) | Unknown Significance | 4 | **2** |
|  | 181 | chr17:g.42636459T>G | NM_001466.3 | c.1403T>G | p.(Leu468Arg) | Likely Pathogenic | 4 | **3** |
| ***GREB1L^+^*** | 5 | chr18:g.19020268C>T | NM_001142966.1 | c.988C>T | p.(Arg330Trp) | Likely Pathogenic | 1 | **3** |
|  | 254* | chr18:g.18963590T>G | NM_001142966.1 | c.111T>G | p.(Phe37Leu) | Likely Pathogenic | 1 | **3** |
| ***HKDC1^+^*** | 97 | chr10:g.70992594G>T | NM_025130.3 | c.301G>T | p.(Gly101Trp) | Likely Pathogenic | 2 | **3** |
|  | 207* | chr10:g.71005968C>T | NM_025130.3 | c.1009C>T | p.(Arg337Trp) | Likely Pathogenic | 2 | **3** |
|  | 239* | chr10:g.71007172C>T | NM_025130.3 | c.1088C>T | p.(Pro363Leu) | Likely Pathogenic | 2 | **3** |
| ***HOOK3^+^*** | 132 | chr8:g.42828459G>A | NM_032410.3 | c.1150G>A | p.(Glu384Lys) | Likely Pathogenic | 3 | **3** |
|  | 232* | chr8:g.42785273T>G | NM_032410.3 | c.225T>G | p.(Asn75Lys) | Likely Pathogenic | 3 | **3** |
| ***LZTR1^+^*** | 37 | chr22:g.21336722G>C | NM_006767.3 | c.62G>C | p.(Arg21Pro) | Likely Pathogenic | 3 | **3** |
|  | 243* | chr22:g.21346593C>T | NM_006767.3 | c.1084C>T | p.(Arg362*) | Pathogenic | 3 | **4** |
| ***MACF1^#^*** | 98 | chr1:g.39827060A>G | NM_012090.5 | c.6296A>G | p.(Lys2099Arg) | Unknown Significance | 3 | **2** |
|  | 167 | chr1:g.39784169G>A | NM_012090.5 | c.3842G>A | p.(Arg1281Gln) | Likely Pathogenic | 3 | **3** |
|  | 249* | chr1:g.39951231G>A | NM_012090.5 | c.16058G>A | p.(Arg5353His) | Likely Pathogenic | 3 | **3** |
|  | 252* | chr1:g.39903438C>T | NM_012090.5 | c.11812-11C>T |  | Unknown Significance | 3 | **2** |
|  | 254* | chr1:g.39907638G>T | NM_012090.5 | c.12510G>T | p.(Trp4170Cys) | Unknown Significance | 3 | **2** |
| ***MTA3*** | 92 | chr2:g.42886935G>A | NM_020744.2 | c.635G>A | p.(Cys212Tyr) | Likely Pathogenic | 5 | **4** |
| ***N4BP2L2*** | 118 | chr13:g.33017223dup | NM_001278432.1 | c.1406dup | p.(Asn469Lysfs*2) | Pathogenic | 4 | **4** |
| ***OSBP*** | 149 | chr11:g.59361521G>A | NM_002556.2 | c.1519C>T | p.(Arg507*) | Pathogenic | 2 | **4** |
| ***PDZRN3^+^*** | 7 | chr3:g.73433616T>G | NM_015009.1 | c.2101A>C | p.(Ser701Arg) | Likely Pathogenic | 2 | **3** |
|  | 211* | chr3:g.73437230A>G | NM_015009.1 | c.1417-10T>C |  | Likely Pathogenic | 2 | **3** |
| ***PHF20^+^*** | 75 | chr20:g.34526911G>A | NM_016436.4 | c.2593G>A | p.(Asp865Asn) | Likely Pathogenic | 4 | **3** |
|  | 246* | chr20:g.34457441C>T | NM_016436.4 | c.890C>T | p.(Pro297Leu) | Likely Pathogenic | 4 | **3** |
| ***POLR2E*** | 152 | chr19:g.1091908T>C | NM_002695.3 | c.233-2A>G |  | Pathogenic | 3 | **4** |
| ***PSMD11*** | 6 | chr17:g.30804536_30804539delCAGA | NM_002815.3 | c.850-3_850delCAGA |  | Pathogenic | 4 | **4** |
| ***PTPRU^+^*** | 150 | chr1:g.29630421C>T | NM_001195001.1 | c.2531C>T | p.(Pro844Leu) | Likely Pathogenic | 5 | **4** |
|  | 246* | chr1:g.29651755A>C | NM_001195001.1 | c.4156A>C | p.(Met1386Leu) | Likely Pathogenic | 5 | **4** |
|  | 250* | chr1:g.29639127G>A | NM_001195001.1 | c.3127G>A | p.(Val1043Ile) | Unknown Significance | 5 | **3** |
| ***RNF31^+^*** | 24 | chr14:g.24624651A>G | NM_017999.4 | c.2333A>G | p.(Tyr778Cys) | Likely Pathogenic | 4 | **3** |
|  | 240* | chr14:g.24624888G>A | NM_017999.4 | c.2480G>A | p.(Arg827His) | Likely Pathogenic | 4 | **3** |
| ***ROS1^+^*** | 27 | chr6:g.117663631del | NM_002944.2 | c.4601del | p.(Asn1534Ilefs*69) | Pathogenic | 2 | **4** |
|  | 253* | chr6:g.117686253T>C | NM_002944.2 | c.3088A>G | p.(Thr1030Ala) | Unknown Significance | 2 | **2** |
| ***RUNX1T1*** | 63 | chr8:g.93029502_93029503insA | NM_004349.3 | c.96_97insT | p.(Pro33Serfs*19) | Pathogenic | 4 | **4** |
| ***SLC38A3^#^*** | 175 | chr3:g.50256096C>A | NM_006841.4 | c.1108C>A | p.(Arg370Ser) | Unknown Significance | 2 | **2** |
|  | 185 | chr3:g.50254901T>C | NM_006841.4 | c.686T>C | p.(Ile229Thr) | Unknown Significance | 2 | **2** |
|  | 197* | chr3:g.50257535C>A | NM_006841.4 | c.1441C>A | p.(Leu481Met) | Unknown Significance | 2 | **2** |
| ***SMARCA5*** | 118 | chr4:g.144449159A>C | NM_003601.3 | c.940A>C | p.(Lys314Gln) | Likely Pathogenic | 6 | **4** |
| ***USP24^#^*** | 152 | chr1:g.55539586G>A | NM_015306.2 | c.7448-7C>T |  | Likely Pathogenic | 2 | **3** |
|  | 178 | chr1:g.55607292T>A | NM_015306.2 | c.2747A>T | p.(Tyr916Phe) | Unknown Significance | 2 | **2** |
|  | 236* | chr1:g.55608741T>C | NM_015306.2 | c.2491A>G | p.(Met831Val) | Unknown Significance | 2 | **2** |
| ***WDR6*** | 163 | chr3:g.49051510G>A | NM_018031.3 | c.2633G>A | p.(Arg878His) | Likely Pathogenic | 5 | **4** |
| ***ZBED4*** | 52 | chr22:g.50277482C>T | NM_014838.2 | c.172C>T | p.(Arg58*) | Pathogenic | 3 | **4** |
| ***ZFC3H1^+^*** | 136 | chr12:g.72004293A>G | NM_144982.4 | c.5885T>C | p.(Leu1962Pro) | Likely Pathogenic | 1 | **3** |
|  | 232* | chr12:g.72050733C>T | NM_144982.4 | c.947G>A | p.(Arg316His) | Likely Pathogenic | 1 | **3** |
| ***ZNF446^#^*** | 99 | chr19:g.58991593G>T | NM_017908.2 | c.853G>T | p.(Gly285Cys) | Unknown Significance | 3 | **2** |
|  | 164 | chr19:g.58991768G>A | NM_017908.2 | c.1028G>A | p.(Trp343*) | Pathogenic | 3 | **4** |
|  | 165 | chr19:g.58991980C>T | NM_017908.2 | c.1240C>T | p.(Arg414Cys) | Unknown Significance | 3 | **2** |
|  | | | | | | | | |
| ***autosomal recessive*** | | | | | | | | |
| ***CHD1L*** | 87 | chr1:g.146757075del | NM_004284 | c.1929del | p.(Arg643Serfs*16) | Pathogenic | 5 | **5** |
| ***DPRX*** | 134 | chr19:g.54140132C>T | NM_001012728 | c.466C>T | p.(Arg156*) | Pathogenic | 2 | **4** |
| ***MYH7B^#^*** | 87 | chr20:g.33588263C>G | NM_020884.3 | c.5075C>G | p.(Thr1692Arg) | Unknown Significance | 3 | **2** |
|  | 89 | chr20:g.33576057_33576059del | NM_020884.3 | c.1705_1707del | p.(Ile569del) | Unknown Significance | 3 | **2** |
|  |  | chr20:g.33584191G>A | NM_020884.3 | c.3112G>A | p.(Val1038Met) | Likely Pathogenic | 3 | **3** |
|  | 230* | chr20:g.33586723G>C | NM_020884.3 | c.4316+5G>C |  | Unknown Significance | 3 | **2** |
|  |  | chr20:g.33575087_33575088del | NM_020884.3 | c.1270_1271del | p.(Ser425Cysfs*2) | Pathogenic | 3 | **4** |
| ***OBSCN^#^*** | 12 | chr1:g.228467044C>T | NM_001098623.2 | c.7295C>T | p.(Thr2432Met) | Likely Pathogenic | 3 | **3** |
|  | 196 | chr1:g.228550402G>C | NM_001098623.2 | c.18787G>C | p.(Ala6263Pro) | Unknown Significance | 3 | **2** |
|  |  | chr1:g.228464400C>T | NM_001098623.2 | c.6470C>T | p.(Ala2157Val) | Likely Pathogenic | 3 | **3** |
| ***PIK3C2A*** | 134 | chr11:g.17167409C>A | NM_002645.2 | c.1640+1G>T |  | Pathogenic | 4 | **4** |
| ***PYGB*** | 87 | chr20:g.25273119A>G | NM_002862.3 | c.2047A>G | p.(Met683Val) | Likely Pathogenic | 5 | **4** |
| ***VWCE*** | 70 | chr11:g.61040737G>A | NM_152718.2 | c.1633C>T | p.(Arg545*) | Pathogenic | 1 | **4** |
|  | | | | | | | | |
| ***X-linked*** | | | | | | | | |
| ***AMMECR1*** | 139 | chrX:g.109560846del | NM_001025580.1 | c.454del | p.(Arg152Aspfs*17) | Pathogenic | 1 | **4** |
| ***CCDC120^#^*** | 112 | chrX:g.48924885C>T | NM_033626.3 | c.1130C>T | p.(Pro377Leu) | Unknown Significance | 2 | **2** |
|  | 137 | chrX:g.48924885C>T | NM_033626.3 | c.1130C>T | p.(Pro377Leu) | Unknown Significance | 2 | **2** |
| ***CXorf27*** | 29 | chrX:g.37850413_37850414del | NM_012274.1 | c.321_322del | p.(Phe107Leufs*2) | Pathogenic | 1 | **4** |
| ***GABRE*** | 102 | chrX:g.151131059G>T | NM_004961.3 | c.399C>A | p.(Tyr133*) | Pathogenic | 1 | **4** |
| ***ITIH6^#^*** | 26 | chrX:g.54784130G>A | NM_198510.2 | c.2377C>T | p.(Gln793*) | Pathogenic | 2 | **4** |
|  | 38 | chrX:g.54785020T>A | NM_198510.2 | c.1487A>T | p.(Asn496Ile) | Unknown Significance | 2 | **2** |
| ***MXRA5^#^*** | 18 | chrX:g.3241943C>T | NM_015419.3 | c.1783G>A | p.(Val595Met) | Unknown Significance | 3 | **2** |
|  | 58 | chrX:g.3241943C>T | NM_015419.3 | c.1783G>A | p.(Val595Met) | Unknown Significance | 3 | **2** |
|  | 252* | chrX:g.3240035G>A | NM_015419.3 | c.3691C>T | p.(Arg1231Trp) | Unknown Significance | 3 | **2** |
| ***SRPX^#^*** | 45 | chrX:g.38031152G>A | NM_006307.4 | c.508C>T | p.(Arg170Trp) | Unknown Significance | 3 | **2** |
|  | 160 | chrX:g.38020230C>G | NM_006307.4 | c.731G>C | p.(Arg244Thr) | Likely Pathogenic | 3 | **3** |
| ***USP51*** | 93 | chrX:g.55515360G>A | NM_201286.3 | c.13C>T | p.(Arg5*) | Pathogenic | 1 | **4** |
| ***ZNF449^#^*** | 29 | chrX:g.134483074G>A | NM_152695.5 | c.394G>A | p.(Val132Met) | Unknown Significance | 2 | **2** |
|  | 166 | chrX:g.134493916T>C | NM_152695.5 | c.659T>C | p.(Leu220Pro) | Unknown Significance | 2 | **2** |
| ^#^: Candidate gene with variants in at least 2 affected individuals with validated segregation, ^+^: Candidate gene with variants in at least 2 affected individuals, whereby segregation could be validated in only one of them, *: variant or its segregation could not be validated by Sanger Sequencing | | | | | | | | |

| Supplementary Table 12. Description of previously reported high-confidence & medium-confidence candidate genes | | | |
| --- | --- | --- | --- |
| **Candidate gene** | **Variant type/ Inheritance mode of previously reported and affected individuals from this study** | **Phenotypical information/ overlap of previously reported and affected individuals from this study** | **Gene function/ further explanation/ conclusion** |
| *IFT81* | Recessive mutations (loss of stop, splice site variant, nonsense variant, missense variant and in frame deletion) in previous studies,^82; 83^ heterozygous mutations in this study | Recessive mutations have been reported to cause ciliopathies, e.g. short rib polydactyly syndrome, which includes severe short stature reported by a reduced birth length (38cm and 42.5 cm).^82; 83^ | Given the fact that in some cases biallelic mutations might lead to a more pronounced, severe and complex phenotype in affected individuals than heterozygous mutations in the same gene, as shown for variants in ACAN^139^, we suggest a similar mechanism for variants in IFT81.^82; 83^ |
| *AMMECR1* | X-linked loss of function variants in an affected individual from this study and previously reported individuals (5 families) ^140; 141^ and missense mutations in one family^142^ | In the family reported by Basel-Vanagaite et al., elliptocytosis, midface hypoplasia, small lips, hearing loss and proportionate short stature were diagnosed.^140^ Affected individuals in the family reported by Andreoletti et al. present with nephrocalcinosis, developmental delay, midface hypoplasia, deafness, elliptocytosis and short stature. ^142^ Five individuals reported by Moyses-Oliveira et al. showed short stature, cardiac and skeletal abnormalities and hearing loss. ^141^ The affected individual reported here also presents with proportionate short stature of about 3 SDS below the age related average, a broad and flat face and small lips, whereas no indication of hearing loss, heart failure or kidney defects was observed and we are currently examining whether elliptocytosis can be added to his symptomatology. | Knockdown in zebrafish caused a phenotype remniscient of the phenotype reported in the affected individuals by Moyses-Oliveira.^141^ Moreover, a compensatory effect was suggested due to increased expression of AMMECR1L and could be also investigated in the cells of one affected individual from this study.^141^ The suggested effects on cell cycle control remain to be evaluated.  Consequently, this study, in combination with previous reports^140-142^ thus suggests that disrupting variants in *AMMECR1* might be causative for short stature and parts of the classical AMME syndrome (Alport syndrome, mental retardation, midface hypoplasia, and elliptocytosis) which is caused by deletions of a region including *AMMECR1.^143^* |
| *BRD4* | Likely pathogenic de novo missense variant in one of the affected individuals in this study, one family with an autosomal dominant segregated missense variant in BRD4 (c.910C>T, p.His304Tyr) in three affected family members reported by Jin et al. ^144^ | Affected individuals described by Jin. et al. suffered from bilateral congenital cataracts, short stature, macrocephaly, and minor skeletal anomalies. The affected individual reported here does not show cataract, but a similar short stature phenotype. | We assume that variants in BRD4 account for the skeletal phenotype including short stature in the affected individuals reported until now. ^144^ This is also supported by the fact that heterozygous knockout of Brd4 in mice results in postnatal growth defects due to a reduced proliferation rate.^145^ One could assume that a proportion of the affected individuals with mutations in BRD4 present with cataracts as a part of the caused phenotypic spectrum, as also in mouse models only 7 of 34 heterozygous mice deficient of Brd4 present with cataracts. But for sure, in order to determine frequency statistics in human, a larger number of affected individuals with mutations in BRD4 have to be evaluated. |
| *FZD2* | Two affected individuals in this study with missense variants (variant of unknown significance and likely pathogenic, autosomal dominant mode of inheritance), two affected individuals from previous studies with nonsense mutations (autosomal dominant)^146; 147^ | Nonsense mutations in FZD2 were reported to cause Robinow-syndrome like phenotypes (omodysplasia).^146; 147^ Only one of two affected individuals reported by Saal et al. presents with short stature, and also the affected individual reported by Nagasaki et al. was 2.8 SD below the average height. Thus, it could not be clearly stated whether short stature resulted from the reported mutations. Nonetheless, the further phenotype of the reported affected individuals was phenotypically strongly overlapping the phenotypic features of one of the affected individuals reported in this study. In respect thereof, Nagasaki et al. describe their affected individual to display elbow restriction and a mild facial dysmorphism including a similar nasal phenotype, a long philtrum and low-set ears such as the affected individual reported here. As in the other family in his study, a variant of unknown significance tis located in FZD2 and there is no striking phenotypic overlap to the reported cases, it is unclear whether this variant is causative for the affected individual’s phenotype or potentially another variant better explains the observed phenotype. | Considering the significant phenotypical similarities to the previously reported affected individuals, we would assume that also the likely pathogenic missense mutation in one of the families reported here causes omodysplasia based on disrupted FZD2 due to its location in the Frizzled/Smoothened family membrane region. In sum, at least one of the affected individuals with a mutation in FZD2 provides further evidence that modifications of this gene cause a Robinow-syndrome like phenotype. |
| *LZTR1* | Two affected individuals in this study with one de novo missense and one nonsense variant without validated segregation, two affected individuals from previous studies with nonsense mutations, 7 autosomal dominant missense variants (5 of them scored as probably pathogenic) ^148^, biallelic mutations in affected individuals from 12 families^149^ | The previously reported affected individuals with autosomal dominant variants presented with facial features typical for noonan syndrome, cardiac abnormalities and short stature in only one family.^148^ The authors suggest that LZTR1 might be relevant to assign NF1 for degradation and further hyperactivate RAS-signaling. In contrast, a recent article by Johnston et al. reports biallelic mutations as causative for noonan syndrome ranging from lethal to only mildly affected affected individuals.^149^ Their parents with heterozygous mutations are reported to be unaffected. In our study, the affected individual with the *de novo* missense mutation showed proportionate short stature as well as dystrophy and white spots. The other affected individual with the heterozygous stop mutation presented only with proportionate short stature. | Whether heterozygous, homozygous mutations or both are causative for short stature from the noonan syndrome family still needs to be finally evaluated. |
| *ZBED4* | De novo loss of start codon variant in one previously reported^34^ and de novo nonsense variant in one affected individual from this study | In both affected individuals, a MRI was normal and they presented with a delayed bone age. Guo et al. reported that the affected individual from his study presented a robust response to the therapy, whereas we do not yet possess information about the response in the affected individual from this study. | In addition, a low-frequency missense variant in *ZBED4* was reported by Marouli et al. to be significantly associated with height further implying the gene as a valid candidate for growth-associated phenotypes.^150^ |

Supplementary References

1. Delude, C.M. (2015). Deep phenotyping: The details of disease. Nature 527, S14-15.

2. Kohler, S., Vasilevsky, N.A., Engelstad, M., Foster, E., McMurry, J., Ayme, S., Baynam, G., Bello, S.M., Boerkoel, C.F., Boycott, K.M., et al. (2017). The Human Phenotype Ontology in 2017. Nucleic acids research 45, D865-D876.

3. Hauer, N.N., Popp, B., Schoeller, E., Schuhmann, S., Heath, K.E., Hisado-Oliva, A., Klinger, P., Kraus, C., Trautmann, U., Zenker, M., et al. (2017). Clinical relevance of systematic phenotyping and exome sequencing in patients with short stature. GENETICS in MEDICINE.

4. McKenna, A., Hanna, M., Banks, E., Sivachenko, A., Cibulskis, K., Kernytsky, A., Garimella, K., Altshuler, D., Gabriel, S., Daly, M., et al. (2010). The Genome Analysis Toolkit: a MapReduce framework for analyzing next-generation DNA sequencing data. Genome Res 20, 1297-1303.

5. DePristo, M.A., Banks, E., Poplin, R., Garimella, K.V., Maguire, J.R., Hartl, C., Philippakis, A.A., del Angel, G., Rivas, M.A., Hanna, M., et al. (2011). A framework for variation discovery and genotyping using next-generation DNA sequencing data. Nat Genet 43, 491-498.

6. Genomes Project, C., Auton, A., Brooks, L.D., Durbin, R.M., Garrison, E.P., Kang, H.M., Korbel, J.O., Marchini, J.L., McCarthy, S., McVean, G.A., et al. (2015). A global reference for human genetic variation. Nature 526, 68-74.

7. Lek, M., Karczewski, K., Minikel, E., Samocha, K., Banks, E., Fennell, T., O'Donnell-Luria, A., Ware, J., Hill, A., Cummings, B., et al. (2016). Analysis of protein-coding genetic variation in 60,706 humans. bioRxiv.

8. Consortium, E.A., Lek, M., Karczewski, K., Minikel, E., Samocha, K., Banks, E., Fennell, T., O'Donnell-Luria, A., Ware, J., Hill, A., et al. (2016). Analysis of protein-coding genetic variation in 60,706 humans.

9. Kircher, M., Witten, D.M., Jain, P., O'Roak, B.J., Cooper, G.M., and Shendure, J. (2014). A general framework for estimating the relative pathogenicity of human genetic variants. Nat Genet 46, 310-315.

10. Jian, X., Boerwinkle, E., and Liu, X. (2014). In silico prediction of splice-altering single nucleotide variants in the human genome. Nucleic acids research 42, 13534-13544.

11. Xiong, H.Y., Alipanahi, B., Lee, L.J., Bretschneider, H., Merico, D., Yuen, R.K., Hua, Y., Gueroussov, S., Najafabadi, H.S., Hughes, T.R., et al. (2015). RNA splicing. The human splicing code reveals new insights into the genetic determinants of disease. Science 347, 1254806.

12. Cingolani, P., Patel, V.M., Coon, M., Nguyen, T., Land, S.J., Ruden, D.M., and Lu, X. (2012). Using Drosophila melanogaster as a Model for Genotoxic Chemical Mutational Studies with a New Program, SnpSift. Frontiers in genetics 3, 35.

13. Berndt, S.I., Gustafsson, S., Magi, R., Ganna, A., Wheeler, E., Feitosa, M.F., Justice, A.E., Monda, K.L., Croteau-Chonka, D.C., Day, F.R., et al. (2013). Genome-wide meta-analysis identifies 11 new loci for anthropometric traits and provides insights into genetic architecture. Nature genetics 45, 501-512.

14. Carty, C.L., Johnson, N.A., Hutter, C.M., Reiner, A.P., Peters, U., Tang, H., and Kooperberg, C. (2012). Genome-wide association study of body height in African Americans: the Women's Health Initiative SNP Health Association Resource (SHARe). Hum Mol Genet 21, 711-720.

15. Cousminer, D.L., Berry, D.J., Timpson, N.J., Ang, W., Thiering, E., Byrne, E.M., Taal, H.R., Huikari, V., Bradfield, J.P., Kerkhof, M., et al. (2013). Genome-wide association and longitudinal analyses reveal genetic loci linking pubertal height growth, pubertal timing and childhood adiposity. Hum Mol Genet 22, 2735-2747.

16. Estrada, K., Krawczak, M., Schreiber, S., van Duijn, K., Stolk, L., van Meurs, J.B., Liu, F., Penninx, B.W., Smit, J.H., Vogelzangs, N., et al. (2009). A genome-wide association study of northwestern Europeans involves the C-type natriuretic peptide signaling pathway in the etiology of human height variation. Hum Mol Genet 18, 3516-3524.

17. Gudbjartsson, D.F., Walters, G.B., Thorleifsson, G., Stefansson, H., Halldorsson, B.V., Zusmanovich, P., Sulem, P., Thorlacius, S., Gylfason, A., Steinberg, S., et al. (2008). Many sequence variants affecting diversity of adult human height. Nature genetics 40, 609-615.

18. Hao, Y., Liu, X., Lu, X., Yang, X., Wang, L., Chen, S., Li, H., Li, J., Cao, J., Chen, J., et al. (2013). Genome-wide association study in Han Chinese identifies three novel loci for human height. Hum Genet 132, 681-689.

19. He, M., Xu, M., Zhang, B., Liang, J., Chen, P., Lee, J.Y., Johnson, T.A., Li, H., Yang, X., Dai, J., et al. (2015). Meta-analysis of genome-wide association studies of adult height in East Asians identifies 17 novel loci. Human molecular genetics 24, 1791-1800.

20. Johansson, A., Marroni, F., Hayward, C., Franklin, C.S., Kirichenko, A.V., Jonasson, I., Hicks, A.A., Vitart, V., Isaacs, A., Axenovich, T., et al. (2009). Common variants in the JAZF1 gene associated with height identified by linkage and genome-wide association analysis. Hum Mol Genet 18, 373-380.

21. Kim, J.J., Lee, H.I., Park, T., Kim, K., Lee, J.E., Cho, N.H., Shin, C., Cho, Y.S., Lee, J.Y., Han, B.G., et al. (2010). Identification of 15 loci influencing height in a Korean population. J Hum Genet 55, 27-31.

22. Lango Allen, H., Estrada, K., Lettre, G., Berndt, S.I., Weedon, M.N., Rivadeneira, F., Willer, C.J., Jackson, A.U., Vedantam, S., Raychaudhuri, S., et al. (2010). Hundreds of variants clustered in genomic loci and biological pathways affect human height. Nature 467, 832-838.

23. Lei, S.F., Yang, T.L., Tan, L.J., Chen, X.D., Guo, Y., Guo, Y.F., Zhang, L., Liu, X.G., Yan, H., Pan, F., et al. (2009). Genome-wide association scan for stature in Chinese: evidence for ethnic specific loci. Hum Genet 125, 1-9.

24. Lettre, G., Jackson, A.U., Gieger, C., Schumacher, F.R., Berndt, S.I., Sanna, S., Eyheramendy, S., Voight, B.F., Butler, J.L., Guiducci, C., et al. (2008). Identification of ten loci associated with height highlights new biological pathways in human growth. Nature genetics 40, 584-591.

25. Lui, J.C., Nilsson, O., Chan, Y., Palmer, C.D., Andrade, A.C., Hirschhorn, J.N., and Baron, J. (2012). Synthesizing genome-wide association studies and expression microarray reveals novel genes that act in the human growth plate to modulate height. Human molecular genetics 21, 5193-5201.

26. N'Diaye, A., Chen, G.K., Palmer, C.D., Ge, B., Tayo, B., Mathias, R.A., Ding, J., Nalls, M.A., Adeyemo, A., Adoue, V., et al. (2011). Identification, replication, and fine-mapping of Loci associated with adult height in individuals of african ancestry. PLoS genetics 7, e1002298.

27. Okada, Y., Kamatani, Y., Takahashi, A., Matsuda, K., Hosono, N., Ohmiya, H., Daigo, Y., Yamamoto, K., Kubo, M., Nakamura, Y., et al. (2010). A genome-wide association study in 19 633 Japanese subjects identified LHX3-QSOX2 and IGF1 as adult height loci. Hum Mol Genet 19, 2303-2312.

28. Sanna, S., Jackson, A.U., Nagaraja, R., Willer, C.J., Chen, W.M., Bonnycastle, L.L., Shen, H., Timpson, N., Lettre, G., Usala, G., et al. (2008). Common variants in the GDF5-UQCC region are associated with variation in human height. Nature genetics 40, 198-203.

29. Soranzo, N., Rivadeneira, F., Chinappen-Horsley, U., Malkina, I., Richards, J.B., Hammond, N., Stolk, L., Nica, A., Inouye, M., Hofman, A., et al. (2009). Meta-analysis of genome-wide scans for human adult stature identifies novel Loci and associations with measures of skeletal frame size. PLoS Genet 5, e1000445.

30. Tonjes, A., Koriath, M., Schleinitz, D., Dietrich, K., Bottcher, Y., Rayner, N.W., Almgren, P., Enigk, B., Richter, O., Rohm, S., et al. (2009). Genetic variation in GPR133 is associated with height: genome wide association study in the self-contained population of Sorbs. Hum Mol Genet 18, 4662-4668.

31. van der Valk, R.J., Kreiner-Moller, E., Kooijman, M.N., Guxens, M., Stergiakouli, E., Saaf, A., Bradfield, J.P., Geller, F., Hayes, M.G., Cousminer, D.L., et al. (2014). A novel common variant in DCST2 is associated with length in early life and height in adulthood. Human molecular genetics.

32. Weedon, M.N., Lango, H., Lindgren, C.M., Wallace, C., Evans, D.M., Mangino, M., Freathy, R.M., Perry, J.R., Stevens, S., Hall, A.S., et al. (2008). Genome-wide association analysis identifies 20 loci that influence adult height. Nat Genet 40, 575-583.

33. Wood, A.R., Esko, T., Yang, J., Vedantam, S., Pers, T.H., Gustafsson, S., Chu, A.Y., Estrada, K., Luan, J., Kutalik, Z., et al. (2014). Defining the role of common variation in the genomic and biological architecture of adult human height. Nature genetics.

34. Guo, M.H., Shen, Y., Walvoord, E.C., Miller, T.C., Moon, J.E., Hirschhorn, J.N., and Dauber, A. (2014). Whole exome sequencing to identify genetic causes of short stature. Hormone research in paediatrics 82, 44-52.

35. Canton, A.P., Costa, S.S., Rodrigues, T.C., Bertola, D.R., Malaquias, A.C., Correa, F.A., Arnhold, I.J., Rosenberg, C., and Jorge, A.A. (2014). Genome-wide screening of copy number variants in children born small for gestational age reveals several candidate genes involved in growth pathways. Eur J Endocrinol 171, 253-262.

36. van Duyvenvoorde, H.A., Lui, J.C., Kant, S.G., Oostdijk, W., Gijsbers, A.C., Hoffer, M.J., Karperien, M., Walenkamp, M.J., Noordam, C., Voorhoeve, P.G., et al. (2014). Copy number variants in patients with short stature. Eur J Hum Genet 22, 602-609.

37. Zahnleiter, D., Uebe, S., Ekici, A.B., Hoyer, J., Wiesener, A., Wieczorek, D., Kunstmann, E., Reis, A., Doerr, H.G., Rauch, A., et al. (2013). Rare copy number variants are a common cause of short stature. PLoS genetics 9, e1003365.

38. Zhang, Y.P., Deng, F.Y., Yang, T.L., Zhang, F., Chen, X.D., Shen, H., Zhu, X.Z., Tian, Q., and Deng, H.W. (2012). Genome-wide association study identified CNP12587 region underlying height variation in Chinese females. PLoS One 7, e44292.

39. Firth, H.V., Richards, S.M., Bevan, A.P., Clayton, S., Corpas, M., Rajan, D., Van Vooren, S., Moreau, Y., Pettett, R.M., and Carter, N.P. (2009). DECIPHER: Database of Chromosomal Imbalance and Phenotype in Humans Using Ensembl Resources. American journal of human genetics 84, 524-533.

40. Eppig, J.T., Blake, J.A., Bult, C.J., Kadin, J.A., Richardson, J.E., and Mouse Genome Database, G. (2015). The Mouse Genome Database (MGD): facilitating mouse as a model for human biology and disease. Nucleic acids research 43, D726-736.

41. Howe, D.G., Bradford, Y.M., Conlin, T., Eagle, A.E., Fashena, D., Frazer, K., Knight, J., Mani, P., Martin, R., Moxon, S.A., et al. (2013). ZFIN, the Zebrafish Model Organism Database: increased support for mutants and transgenics. Nucleic acids research 41, D854-860.

42. Szklarczyk, D., Franceschini, A., Wyder, S., Forslund, K., Heller, D., Huerta-Cepas, J., Simonovic, M., Roth, A., Santos, A., Tsafou, K.P., et al. (2015). STRING v10: protein-protein interaction networks, integrated over the tree of life. Nucleic acids research 43, D447-452.

43. Lee, H., Pine, P.S., McDaniel, J., Salit, M., and Oliver, B. (2016). External RNA Controls Consortium Beta Version Update. Journal of genomics 4, 19-22.

44. Baker, S.C., Bauer, S.R., Beyer, R.P., Brenton, J.D., Bromley, B., Burrill, J., Causton, H., Conley, M.P., Elespuru, R., Fero, M., et al. (2005). The External RNA Controls Consortium: a progress report. Nature methods 2, 731-734.

45. Hafren, A., Eskelin, K., and Makinen, K. (2013). Ribosomal protein P0 promotes Potato virus A infection and functions in viral translation together with VPg and eIF(iso)4E. J Virol 87, 4302-4312.

46. Dobin, A., Davis, C.A., Schlesinger, F., Drenkow, J., Zaleski, C., Jha, S., Batut, P., Chaisson, M., and Gingeras, T.R. (2013). STAR: ultrafast universal RNA-seq aligner. Bioinformatics 29, 15-21.

47. Liao, Y., Smyth, G.K., and Shi, W. (2014). featureCounts: an efficient general purpose program for assigning sequence reads to genomic features. Bioinformatics 30, 923-930.

48. R Core Team. (2014). R: A language and environment for statistical computing.

49. Anders, S., and Huber, W. (2010). Differential expression analysis for sequence count data. Genome biology 11, R106.

50. Huang da, W., Sherman, B.T., and Lempicki, R.A. (2009). Bioinformatics enrichment tools: paths toward the comprehensive functional analysis of large gene lists. Nucleic acids research 37, 1-13.

51. Huang da, W., Sherman, B.T., and Lempicki, R.A. (2009). Systematic and integrative analysis of large gene lists using DAVID bioinformatics resources. Nature protocols 4, 44-57.

52. Gene Ontology, C. (2015). Gene Ontology Consortium: going forward. Nucleic acids research 43, D1049-1056.

53. Ashburner, M., Ball, C.A., Blake, J.A., Botstein, D., Butler, H., Cherry, J.M., Davis, A.P., Dolinski, K., Dwight, S.S., Eppig, J.T., et al. (2000). Gene ontology: tool for the unification of biology. The Gene Ontology Consortium. Nature genetics 25, 25-29.

54. Jiao, X., Sherman, B.T., Huang da, W., Stephens, R., Baseler, M.W., Lane, H.C., and Lempicki, R.A. (2012). DAVID-WS: a stateful web service to facilitate gene/protein list analysis. Bioinformatics 28, 1805-1806.

55. Benjamini, Y., and Hochberg, Y. (1995). Controlling the False Discovery Rate: A Practical and Powerful Approach to Multiple Testing. Journal of the Royal Statistical Society Series B (Methodological) 57, 289-300.

56. Kinsella, R.J., Kahari, A., Haider, S., Zamora, J., Proctor, G., Spudich, G., Almeida-King, J., Staines, D., Derwent, P., Kerhornou, A., et al. (2011). Ensembl BioMarts: a hub for data retrieval across taxonomic space. Database (Oxford) 2011, bar030.

57. Leonard, T.A., Rozycki, B., Saidi, L.F., Hummer, G., and Hurley, J.H. (2011). Crystal structure and allosteric activation of protein kinase C betaII. Cell 144, 55-66.

58. Hussain, S.A., Carafoli, F., and Hohenester, E. (2011). Determinants of laminin polymerization revealed by the structure of the alpha5 chain amino-terminal region. EMBO reports 12, 276-282.

59. Cordle, J., Johnson, S., Tay, J.Z., Roversi, P., Wilkin, M.B., de Madrid, B.H., Shimizu, H., Jensen, S., Whiteman, P., Jin, B., et al. (2008). A conserved face of the Jagged/Serrate DSL domain is involved in Notch trans-activation and cis-inhibition. Nature structural & molecular biology 15, 849-857.

60. Harrison, D., Hussain, S.A., Combs, A.C., Ervasti, J.M., Yurchenco, P.D., and Hohenester, E. (2007). Crystal structure and cell surface anchorage sites of laminin alpha1LG4-5. The Journal of biological chemistry 282, 11573-11581.

61. Janssen, B.J., Robinson, R.A., Perez-Branguli, F., Bell, C.H., Mitchell, K.J., Siebold, C., and Jones, E.Y. (2010). Structural basis of semaphorin-plexin signalling. Nature 467, 1118-1122.

62. Pieper, U., Webb, B.M., Dong, G.Q., Schneidman-Duhovny, D., Fan, H., Kim, S.J., Khuri, N., Spill, Y.G., Weinkam, P., Hammel, M., et al. (2014). ModBase, a database of annotated comparative protein structure models and associated resources. Nucleic acids research 42, D336-346.

63. Zimmermann, L., Stephens, A., Nam, S.Z., Rau, D., Kubler, J., Lozajic, M., Gabler, F., Soding, J., Lupas, A.N., and Alva, V. (2017). A Completely Reimplemented MPI Bioinformatics Toolkit with a New HHpred Server at its Core. Journal of molecular biology.

64. Webb, B., and Sali, A. (2017). Protein Structure Modeling with MODELLER. Methods in molecular biology 1654, 39-54.

65. Guex, N., and Peitsch, M.C. (1997). SWISS-MODEL and the Swiss-PdbViewer: an environment for comparative protein modeling. Electrophoresis 18, 2714-2723.

66. Sayle, R.A., and Milner-White, E.J. (1995). RASMOL: biomolecular graphics for all. Trends in biochemical sciences 20, 374.

67. Petersen, T.N., Brunak, S., von Heijne, G., and Nielsen, H. (2011). SignalP 4.0: discriminating signal peptides from transmembrane regions. Nature methods 8, 785-786.

68. Hornbeck, P.V., Zhang, B., Murray, B., Kornhauser, J.M., Latham, V., and Skrzypek, E. (2015). PhosphoSitePlus, 2014: mutations, PTMs and recalibrations. Nucleic acids research 43, D512-520.

69. Savkur, R.S., and Burris, T.P. (2004). The coactivator LXXLL nuclear receptor recognition motif. The journal of peptide research : official journal of the American Peptide Society 63, 207-212.

70. Reinken, L., Stolley, H., Droese, W., and van Oost, G. (1980). [Longitudinal data of physical growth of healthy children. II. Height, weight, skinfold thickness of children aged 1.5--16 years (author's transl)]. Klin Padiatr 192, 25-33.

71. Song, L., and Fricker, L.D. (1997). Cloning and expression of human carboxypeptidase Z, a novel metallocarboxypeptidase. The Journal of biological chemistry 272, 10543-10550.

72. Wang, L., Shao, Y.Y., and Ballock, R.T. (2009). Carboxypeptidase Z (CPZ) links thyroid hormone and Wnt signaling pathways in growth plate chondrocytes. Journal of bone and mineral research : the official journal of the American Society for Bone and Mineral Research 24, 265-273.

73. Moeller, C., Swindell, E.C., Kispert, A., and Eichele, G. (2003). Carboxypeptidase Z (CPZ) modulates Wnt signaling and regulates the development of skeletal elements in the chicken. Development 130, 5103-5111.

74. Wit, J.M., Oostdijk, W., Losekoot, M., van Duyvenvoorde, H.A., Ruivenkamp, C.A., and Kant, S.G. (2015). MECHANISMS IN ENDOCRINOLOGY: Novel genetic causes of short stature. European journal of endocrinology / European Federation of Endocrine Societies.

75. Hirao, K., Natsuka, Y., Tamura, T., Wada, I., Morito, D., Natsuka, S., Romero, P., Sleno, B., Tremblay, L.O., Herscovics, A., et al. (2006). EDEM3, a soluble EDEM homolog, enhances glycoprotein endoplasmic reticulum-associated degradation and mannose trimming. The Journal of biological chemistry 281, 9650-9658.

76. Ninagawa, S., Okada, T., Sumitomo, Y., Kamiya, Y., Kato, K., Horimoto, S., Ishikawa, T., Takeda, S., Sakuma, T., Yamamoto, T., et al. (2014). EDEM2 initiates mammalian glycoprotein ERAD by catalyzing the first mannose trimming step. The Journal of cell biology 206, 347-356.

77. Munkley, J., Vodak, D., Livermore, K.E., James, K., Wilson, B.T., Knight, B., McCullagh, P., McGrath, J., Crundwell, M., Harries, L.W., et al. (2016). Glycosylation is an Androgen-Regulated Process Essential for Prostate Cancer Cell Viability. EBioMedicine 8, 103-116.

78. Prakash, S., Robbins, P.W., and Wyler, D.J. (1995). Cloning and analysis of murine cDNA that encodes a fibrogenic lymphokine, fibrosin. Proceedings of the National Academy of Sciences of the United States of America 92, 2154-2158.

79. Prakash, S., Paul, W.E., and Robbins, P.W. (2007). Fibrosin, a novel fibrogenic cytokine, modulates expression of myofibroblasts. Experimental and molecular pathology 82, 42-48.

80. Lucker, B.F., Behal, R.H., Qin, H., Siron, L.C., Taggart, W.D., Rosenbaum, J.L., and Cole, D.G. (2005). Characterization of the intraflagellar transport complex B core: direct interaction of the IFT81 and IFT74/72 subunits. The Journal of biological chemistry 280, 27688-27696.

81. Bhogaraju, S., Cajanek, L., Fort, C., Blisnick, T., Weber, K., Taschner, M., Mizuno, N., Lamla, S., Bastin, P., Nigg, E.A., et al. (2013). Molecular basis of tubulin transport within the cilium by IFT74 and IFT81. Science 341, 1009-1012.

82. Perrault, I., Halbritter, J., Porath, J.D., Gerard, X., Braun, D.A., Gee, H.Y., Fathy, H.M., Saunier, S., Cormier-Daire, V., Thomas, S., et al. (2015). IFT81, encoding an IFT-B core protein, as a very rare cause of a ciliopathy phenotype. Journal of medical genetics 52, 657-665.

83. Duran, I., Taylor, S.P., Zhang, W., Martin, J., Forlenza, K.N., Spiro, R.P., Nickerson, D.A., Bamshad, M., Cohn, D.H., and Krakow, D. (2016). Destabilization of the IFT-B cilia core complex due to mutations in IFT81 causes a Spectrum of Short-Rib Polydactyly Syndrome. Scientific reports 6, 34232.

84. Kim, H.J., Jang, S.H., Jeong, Y.A., Ryu, P.D., Kim, D.Y., and Lee, S.Y. (2010). Involvement of Kv4.1 K(+) channels in gastric cancer cell proliferation. Biological & pharmaceutical bulletin 33, 1754-1757.

85. Serodio, P., and Rudy, B. (1998). Differential expression of Kv4 K+ channel subunits mediating subthreshold transient K+ (A-type) currents in rat brain. Journal of neurophysiology 79, 1081-1091.

86. Pak, M.D., Baker, K., Covarrubias, M., Butler, A., Ratcliffe, A., and Salkoff, L. (1991). mShal, a subfamily of A-type K+ channel cloned from mammalian brain. Proceedings of the National Academy of Sciences of the United States of America 88, 4386-4390.

87. Isbrandt, D., Leicher, T., Waldschutz, R., Zhu, X., Luhmann, U., Michel, U., Sauter, K., and Pongs, O. (2000). Gene structures and expression profiles of three human KCND (Kv4) potassium channels mediating A-type currents I(TO) and I(SA). Genomics 64, 144-154.

88. Spenle, C., Simon-Assmann, P., Orend, G., and Miner, J.H. (2013). Laminin alpha5 guides tissue patterning and organogenesis. Cell adhesion & migration 7, 90-100.

89. Ritie, L., Spenle, C., Lacroute, J., Bolcato-Bellemin, A.L., Lefebvre, O., Bole-Feysot, C., Jost, B., Klein, A., Arnold, C., Kedinger, M., et al. (2012). Abnormal Wnt and PI3Kinase signaling in the malformed intestine of lama5 deficient mice. PloS one 7, e37710.

90. Hong, D., Chen, H.X., Yu, H.Q., Liang, Y., Wang, C., Lian, Q.Q., Deng, H.T., and Ge, R.S. (2010). Morphological and proteomic analysis of early stage of osteoblast differentiation in osteoblastic progenitor cells. Experimental cell research 316, 2291-2300.

91. Braun, D.A., Warejko, J.K., Ashraf, S., Tan, W., Daga, A., Schneider, R., Hermle, T., Jobst-Schwan, T., Widmeier, E., Majmundar, A.J., et al. (2018). Genetic variants in the LAMA5 gene in pediatric nephrotic syndrome. Nephrology, dialysis, transplantation : official publication of the European Dialysis and Transplant Association - European Renal Association.

92. Sampaolo, S., Napolitano, F., Tirozzi, A., Reccia, M.G., Lombardi, L., Farina, O., Barra, A., Cirillo, F., Melone, M.A.B., Gianfrancesco, F., et al. (2017). Identification of the first dominant mutation of LAMA5 gene causing a complex multisystem syndrome due to dysfunction of the extracellular matrix. Journal of medical genetics 54, 710-720.

93. De Luca, M., Crocco, P., De Rango, F., Passarino, G., and Rose, G. (2016). Association of the Laminin, Alpha 5 (LAMA5) rs4925386 with height and longevity in an elderly population from Southern Italy. Mechanisms of ageing and development 155, 55-59.

94. De Luca, M., Crocco, P., Wiener, H., Tiwari, H.K., Passarino, G., and Rose, G. (2011). Association of a common LAMA5 variant with anthropometric and metabolic traits in an Italian cohort of healthy elderly subjects. Experimental gerontology 46, 60-64.

95. Shannon, M.B., Patton, B.L., Harvey, S.J., and Miner, J.H. (2006). A hypomorphic mutation in the mouse laminin alpha5 gene causes polycystic kidney disease. Journal of the American Society of Nephrology : JASN 17, 1913-1922.

96. Zhang, J., and Fondell, J.D. (1999). Identification of mouse TRAP100: a transcriptional coregulatory factor for thyroid hormone and vitamin D receptors. Molecular endocrinology 13, 1130-1140.

97. Durr, K., Holzschuh, J., Filippi, A., Ettl, A.K., Ryu, S., Shepherd, I.T., and Driever, W. (2006). Differential roles of transcriptional mediator complex subunits Crsp34/Med27, Crsp150/Med14 and Trap100/Med24 during zebrafish retinal development. Genetics 174, 693-705.

98. Pietsch, J., Delalande, J.M., Jakaitis, B., Stensby, J.D., Dohle, S., Talbot, W.S., Raible, D.W., and Shepherd, I.T. (2006). lessen encodes a zebrafish trap100 required for enteric nervous system development. Development 133, 395-406.

99. Wang, L., Lam, G., and Thummel, C.S. (2010). Med24 and Mdh2 are required for Drosophila larval salivary gland cell death. Developmental dynamics : an official publication of the American Association of Anatomists 239, 954-964.

100. Ito, M., Okano, H.J., Darnell, R.B., and Roeder, R.G. (2002). The TRAP100 component of the TRAP/Mediator complex is essential in broad transcriptional events and development. The EMBO journal 21, 3464-3475.

101. Tamagnone, L., Artigiani, S., Chen, H., He, Z., Ming, G.I., Song, H., Chedotal, A., Winberg, M.L., Goodman, C.S., Poo, M., et al. (1999). Plexins are a large family of receptors for transmembrane, secreted, and GPI-anchored semaphorins in vertebrates. Cell 99, 71-80.

102. Wang, Q., Chiu, S.L., Koropouli, E., Hong, I., Mitchell, S., Easwaran, T.P., Hamilton, N.R., Gustina, A.S., Zhu, Q., Ginty, D.D., et al. (2017). Neuropilin-2/PlexinA3 Receptors Associate with GluA1 and Mediate Sema3F-Dependent Homeostatic Scaling in Cortical Neurons. Neuron 96, 1084-1098 e1087.

103. Ben-Zvi, A., Manor, O., Schachner, M., Yaron, A., Tessier-Lavigne, M., and Behar, O. (2008). The Semaphorin receptor PlexinA3 mediates neuronal apoptosis during dorsal root ganglia development. The Journal of neuroscience : the official journal of the Society for Neuroscience 28, 12427-12432.

104. Feldner, J., Reimer, M.M., Schweitzer, J., Wendik, B., Meyer, D., Becker, T., and Becker, C.G. (2007). PlexinA3 restricts spinal exit points and branching of trunk motor nerves in embryonic zebrafish. The Journal of neuroscience : the official journal of the Society for Neuroscience 27, 4978-4983.

105. Murakami, Y., Suto, F., Shimizu, M., Shinoda, T., Kameyama, T., and Fujisawa, H. (2001). Differential expression of plexin-A subfamily members in the mouse nervous system. Developmental dynamics : an official publication of the American Association of Anatomists 220, 246-258.

106. Ton, Q.V., and Kathryn Iovine, M. (2012). Semaphorin3d mediates Cx43-dependent phenotypes during fin regeneration. Developmental biology 366, 195-203.

107. Hofmann, K., Zweier, M., Sticht, H., Zweier, C., Wittmann, W., Hoyer, J., Uebe, S., van Haeringen, A., Thiel, C.T., Ekici, A.B., et al. (2013). Biallelic SEMA3A defects cause a novel type of syndromic short stature. American journal of medical genetics Part A 161A, 2880-2889.

108. Baumann, M., Steichen-Gersdorf, E., Krabichler, B., Muller, T., and Janecke, A.R. (2017). A recognizable type of syndromic short stature with arthrogryposis caused by bi-allelic SEMA3A loss-of-function variants. Clinical genetics 92, 86-90.

109. Cullen, P.J., Hsuan, J.J., Truong, O., Letcher, A.J., Jackson, T.R., Dawson, A.P., and Irvine, R.F. (1995). Identification of a specific Ins(1,3,4,5)P4-binding protein as a member of the GAP1 family. Nature 376, 527-530.

110. Aoki, Y., Niihori, T., Inoue, S., and Matsubara, Y. (2016). Recent advances in RASopathies. Journal of human genetics 61, 33-39.

111. Blanc, L., Ciciotte, S.L., Gwynn, B., Hildick-Smith, G.J., Pierce, E.L., Soltis, K.A., Cooney, J.D., Paw, B.H., and Peters, L.L. (2012). Critical function for the Ras-GTPase activating protein RASA3 in vertebrate erythropoiesis and megakaryopoiesis. Proc Natl Acad Sci U S A 109, 12099-12104.

112. Bassi, M.T., Sperandeo, M.P., Incerti, B., Bulfone, A., Pepe, A., Surace, E.M., Gattuso, C., De Grandi, A., Buoninconti, A., Riboni, M., et al. (1999). SLC7A8, a gene mapping within the lysinuric protein intolerance critical region, encodes a new member of the glycoprotein-associated amino acid transporter family. Genomics 62, 297-303.

113. Kanehisa, M., and Goto, S. (2000). KEGG: kyoto encyclopedia of genes and genomes. Nucleic acids research 28, 27-30.

114. Kanehisa, M., Furumichi, M., Tanabe, M., Sato, Y., and Morishima, K. (2017). KEGG: new perspectives on genomes, pathways, diseases and drugs. Nucleic acids research 45, D353-D361.

115. Kanehisa, M., Sato, Y., Kawashima, M., Furumichi, M., and Tanabe, M. (2016). KEGG as a reference resource for gene and protein annotation. Nucleic acids research 44, D457-462.

116. Kurayama, R., Ito, N., Nishibori, Y., Fukuhara, D., Akimoto, Y., Higashihara, E., Ishigaki, Y., Sai, Y., Miyamoto, K., Endou, H., et al. (2011). Role of amino acid transporter LAT2 in the activation of mTORC1 pathway and the pathogenesis of crescentic glomerulonephritis. Laboratory investigation; a journal of technical methods and pathology 91, 992-1006.

117. Espino Guarch, M., Font-Llitjos, M., Murillo-Cuesta, S., Errasti-Murugarren, E., Celaya, A.M., Girotto, G., Vuckovic, D., Mezzavilla, M., Vilches, C., Bodoy, S., et al. (2018). Mutations in L-type amino acid transporter-2 support SLC7A8 as a novel gene involved in age-related hearing loss. eLife 7.

118. Verrey, F., Closs, E.I., Wagner, C.A., Palacin, M., Endou, H., and Kanai, Y. (2004). CATs and HATs: the SLC7 family of amino acid transporters. Pflugers Archiv : European journal of physiology 447, 532-542.

119. Ogier de Baulny, H., Schiff, M., and Dionisi-Vici, C. (2012). Lysinuric protein intolerance (LPI): a multi organ disease by far more complex than a classic urea cycle disorder. Molecular genetics and metabolism 106, 12-17.

120. Borsani, G., Bassi, M.T., Sperandeo, M.P., De Grandi, A., Buoninconti, A., Riboni, M., Manzoni, M., Incerti, B., Pepe, A., Andria, G., et al. (1999). SLC7A7, encoding a putative permease-related protein, is mutated in patients with lysinuric protein intolerance. Nature genetics 21, 297-301.

121. Tasaki, T., Mulder, L.C., Iwamatsu, A., Lee, M.J., Davydov, I.V., Varshavsky, A., Muesing, M., and Kwon, Y.T. (2005). A family of mammalian E3 ubiquitin ligases that contain the UBR box motif and recognize N-degrons. Molecular and cellular biology 25, 7120-7136.

122. Huh, K.W., DeMasi, J., Ogawa, H., Nakatani, Y., Howley, P.M., and Munger, K. (2005). Association of the human papillomavirus type 16 E7 oncoprotein with the 600-kDa retinoblastoma protein-associated factor, p600. Proceedings of the National Academy of Sciences of the United States of America 102, 11492-11497.

123. Nakaya, T., Ishiguro, K., Belzil, C., Rietsch, A.M., Yu, Q., Mizuno, S., Bronson, R.T., Geng, Y., Nguyen, M.D., Akashi, K., et al. (2013). p600 Plays Essential Roles in Fetal Development. PloS one 8, e66269.

124. Sukalo, M., Fiedler, A., Guzman, C., Spranger, S., Addor, M.C., McHeik, J.N., Oltra Benavent, M., Cobben, J.M., Gillis, L.A., Shealy, A.G., et al. (2014). Mutations in the human UBR1 gene and the associated phenotypic spectrum. Human mutation 35, 521-531.

125. Faesen, A.C., Luna-Vargas, M.P., Geurink, P.P., Clerici, M., Merkx, R., van Dijk, W.J., Hameed, D.S., El Oualid, F., Ovaa, H., and Sixma, T.K. (2011). The differential modulation of USP activity by internal regulatory domains, interactors and eight ubiquitin chain types. Chemistry & biology 18, 1550-1561.

126. Jaspers, N.G., Raams, A., Silengo, M.C., Wijgers, N., Niedernhofer, L.J., Robinson, A.R., Giglia-Mari, G., Hoogstraten, D., Kleijer, W.J., Hoeijmakers, J.H., et al. (2007). First reported patient with human ERCC1 deficiency has cerebro-oculo-facio-skeletal syndrome with a mild defect in nucleotide excision repair and severe developmental failure. American journal of human genetics 80, 457-466.

127. Kashiyama, K., Nakazawa, Y., Pilz, D.T., Guo, C., Shimada, M., Sasaki, K., Fawcett, H., Wing, J.F., Lewin, S.O., Carr, L., et al. (2013). Malfunction of nuclease ERCC1-XPF results in diverse clinical manifestations and causes Cockayne syndrome, xeroderma pigmentosum, and Fanconi anemia. American journal of human genetics 92, 807-819.

128. Karikkineth, A.C., Scheibye-Knudsen, M., Fivenson, E., Croteau, D.L., and Bohr, V.A. (2017). Cockayne syndrome: Clinical features, model systems and pathways. Ageing research reviews 33, 3-17.

129. Perez-Oliva, A.B., Lachaud, C., Szyniarowski, P., Munoz, I., Macartney, T., Hickson, I., Rouse, J., and Alessi, D.R. (2015). USP45 deubiquitylase controls ERCC1-XPF endonuclease-mediated DNA damage responses. The EMBO journal 34, 326-343.

130. Morinaga, T., Yasuda, H., Hashimoto, T., Higashio, K., and Tamaoki, T. (1991). A human alpha-fetoprotein enhancer-binding protein, ATBF1, contains four homeodomains and seventeen zinc fingers. Molecular and cellular biology 11, 6041-6049.

131. Yasuda, H., Mizuno, A., Tamaoki, T., and Morinaga, T. (1994). ATBF1, a multiple-homeodomain zinc finger protein, selectively down-regulates AT-rich elements of the human alpha-fetoprotein gene. Molecular and cellular biology 14, 1395-1401.

132. Miura, Y., Tam, T., Ido, A., Morinaga, T., Miki, T., Hashimoto, T., and Tamaoki, T. (1995). Cloning and characterization of an ATBF1 isoform that expresses in a neuronal differentiation-dependent manner. The Journal of biological chemistry 270, 26840-26848.

133. Sun, X., Fu, X., Li, J., Xing, C., Martin, D.W., Zhang, H.H., Chen, Z., and Dong, J.T. (2012). Heterozygous deletion of Atbf1 by the Cre-loxP system in mice causes preweaning mortality. Genesis 50, 819-827.

134. Jiang, Q., Ni, B., Shi, J., Han, Z., Qi, R., Xu, W., Wang, D., Wang, D.W., and Chen, M. (2014). Down-regulation of ATBF1 activates STAT3 signaling via PIAS3 in pacing-induced HL-1 atrial myocytes. Biochemical and biophysical research communications 449, 278-283.

135. Nojiri, S., Joh, T., Miura, Y., Sakata, N., Nomura, T., Nakao, H., Sobue, S., Ohara, H., Asai, K., and Ito, M. (2004). ATBF1 enhances the suppression of STAT3 signaling by interaction with PIAS3. Biochemical and biophysical research communications 314, 97-103.

136. Domene, H.M., and Fierro-Carrion, G. (2018). Genetic disorders of GH action pathway. Growth hormone & IGF research : official journal of the Growth Hormone Research Society and the International IGF Research Society 38, 19-23.

137. Qi, Y., Ranish, J.A., Zhu, X., Krones, A., Zhang, J., Aebersold, R., Rose, D.W., Rosenfeld, M.G., and Carriere, C. (2008). Atbf1 is required for the Pit1 gene early activation. Proceedings of the National Academy of Sciences of the United States of America 105, 2481-2486.

138. Sobrier, M.L., Tsai, Y.C., Perez, C., Leheup, B., Bouceba, T., Duquesnoy, P., Copin, B., Sizova, D., Penzo, A., Stanger, B.Z., et al. (2016). Functional characterization of a human POU1F1 mutation associated with isolated growth hormone deficiency: a novel etiology for IGHD. Human molecular genetics 25, 472-483.

139. Hauer, N.N., Sticht, H., Boppudi, S., Buttner, C., Kraus, C., Trautmann, U., Zenker, M., Zweier, C., Wiesener, A., Jamra, R.A., et al. (2017). Genetic screening confirms heterozygous mutations in ACAN as a major cause of idiopathic short stature. Scientific reports 7, 12225.

140. Basel-Vanagaite, L., Pillar, N., Isakov, O., Smirin-Yosef, P., Lagovsky, I., Orenstein, N., Salmon-Divon, M., Tamary, H., Zaft, T., Bazak, L., et al. (2017). X-linked elliptocytosis with impaired growth is related to mutated AMMECR1. Gene.

141. Moyses-Oliveira, M., Giannuzzi, G., Fish, R.J., Rosenfeld, J.A., Petit, F., Soares, M.F., Kulikowski, L.D., Di-Battista, A., Zamariolli, M., Xia, F., et al. (2018). Inactivation of AMMECR1 is associated with growth, bone, and heart alterations. Hum Mutat 39, 281-291.

142. Andreoletti, G., Seaby, E.G., Dewing, J.M., O'Kelly, I., Lachlan, K., Gilbert, R.D., and Ennis, S. (2016). AMMECR1: a single point mutation causes developmental delay, midface hypoplasia and elliptocytosis. Journal of medical genetics.

143. Vitelli, F., Meloni, I., Fineschi, S., Favara, F., Tiziana Storlazzi, C., Rocchi, M., and Renieri, A. (2000). Identification and characterization of mouse orthologs of the AMMECR1 and FACL4 genes deleted in AMME syndrome: orthology of Xq22.3 and MmuXF1-F3. Cytogenetics and cell genetics 88, 259-263.

144. Jin, H.S., Kim, J., Kwak, W., Jeong, H., Lim, G.B., and Lee, C.G. (2017). Identification of a Novel Mutation in BRD4 that Causes Autosomal Dominant Syndromic Congenital Cataracts Associated with Other Neuro-Skeletal Anomalies. PLoS One 12, e0169226.

145. Houzelstein, D., Bullock, S.L., Lynch, D.E., Grigorieva, E.F., Wilson, V.A., and Beddington, R.S. (2002). Growth and early postimplantation defects in mice deficient for the bromodomain-containing protein Brd4. Mol Cell Biol 22, 3794-3802.

146. Saal, H.M., Prows, C.A., Guerreiro, I., Donlin, M., Knudson, L., Sund, K.L., Chang, C.F., Brugmann, S.A., and Stottmann, R.W. (2015). A mutation in FRIZZLED2 impairs Wnt signaling and causes autosomal dominant omodysplasia. Human molecular genetics 24, 3399-3409.

147. Nagasaki, K., Nishimura, G., Kikuchi, T., Nyuzuki, H., Sasaki, S., Ogawa, Y., and Saitoh, A. (2018). Nonsense mutations in FZD2 cause autosomal-dominant omodysplasia: Robinow syndrome-like phenotypes. American journal of medical genetics Part A 176, 739-742.

148. Yamamoto, G.L., Aguena, M., Gos, M., Hung, C., Pilch, J., Fahiminiya, S., Abramowicz, A., Cristian, I., Buscarilli, M., Naslavsky, M.S., et al. (2015). Rare variants in SOS2 and LZTR1 are associated with Noonan syndrome. Journal of medical genetics 52, 413-421.

149. Johnston, J.J., van der Smagt, J.J., Rosenfeld, J.A., Pagnamenta, A.T., Alswaid, A., Baker, E.H., Blair, E., Borck, G., Brinkmann, J., Craigen, W., et al. (2018). Autosomal recessive Noonan syndrome associated with biallelic LZTR1 variants. Genetics in medicine : official journal of the American College of Medical Genetics.

150. Marouli, E., Graff, M., Medina-Gomez, C., Lo, K.S., Wood, A.R., Kjaer, T.R., Fine, R.S., Lu, Y., Schurmann, C., Highland, H.M., et al. (2017). Rare and low-frequency coding variants alter human adult height. Nature.
